# An In Vitro Assessment of Immunostimulatory Responses to Ten Model Innate Immune Response Modulating Impurities (IIRMIs) and Peptide Drug Product, Teriparatide

**DOI:** 10.3390/molecules26247461

**Published:** 2021-12-09

**Authors:** Claire K. Holley, Edward Cedrone, Duncan Donohue, Barry W. Neun, Daniela Verthelyi, Eric S. Pang, Marina A. Dobrovolskaia

**Affiliations:** 1 Nanotechnology Characterization Laboratory, Cancer Research Technology Program, Frederick National Laboratory for Cancer Research Sponsored by the National Cancer Institute, Frederick, MD 21702, USA; claire.holley@nih.gov (C.K.H.); edward.cedrone@nih.gov (E.C.); neunb@mail.nih.gov (B.W.N.); 2 Statistics Department, Data Management Services Inc., Frederick National Laboratory for Cancer Research Sponsored by the National Cancer Institute, Frederick, MD 21702, USA; duncan.donohue@nih.gov; 3 Laboratory of Innate Immunity, Division of Biotechnology Review and Research-III, Office of Biotechnology Products, Center for Drug Evaluation and Research, U.S. Food and Drug Administration, Silver Spring, MD 20993, USA; daniela.verthelyi@fda.hhs.gov; 4 Division of Therapeutic Performance, Office of Research and Standards, Office of Generic Drugs, Center for Drug Evaluation and Research, U.S. Food and Drug Administration, Silver Spring, MD 20993, USA

**Keywords:** cytokines, innate immunity, immunogenicity, peptides, teriparatide

## Abstract

Understanding, predicting, and minimizing the immunogenicity of peptide-based therapeutics are of paramount importance for ensuring the safety and efficacy of these products. The so-called anti-drug antibodies (ADA) may have various clinical consequences, including but not limited to the alteration in the product’s distribution, biological activity, and clearance profiles. The immunogenicity of biotherapeutics can be influenced by immunostimulation triggered by the presence of innate immune response modulating impurities (IIRMIs) inadvertently introduced during the manufacturing process. Herein, we evaluate the applicability of several in vitro assays (i.e., complement activation, leukocyte proliferation, and cytokine secretion) for the screening of innate immune responses induced by ten common IIRMIs (*Bacillus subtilis* flagellin, FSL-1, zymosan, ODN2006, poly(I:C) HMW, poly(I:C) LMW, CLO75, MDP, ODN2216, and *Escherichia coli* O111:B4 LPS), and a model biotherapeutic Forteo™ (teriparatide). Our study identifies cytokine secretion from healthy human donor peripheral blood mononuclear cells (PBMC) as a sensitive method for the in vitro monitoring of innate immune responses to individual IIRMIs and teriparatide (TP). We identify signature cytokines, evaluate both broad and narrow multiplex cytokine panels, and discuss how the assay logistics influence the performance of this in vitro assay.

## 1. Introduction

Repeated administration of therapeutic drug products was shown to trigger unwanted immune responses and the production of antibodies capable of neutralizing both the therapeutic protein and its endogenous counterparts [1,2,3]. Antibodies to recombinant biotechnology therapeutics come in a variety of isotypes (e.g., IgM vs. IgG vs. IgE), allotypes (e.g., reflecting genetic differences between IgG of biologically unrelated individuals), idiotypes (e.g., reflecting binding to specific epitopes within antibody variable sites), and may ultimately lead to different functional consequences for the host (e.g., binding, PK-altering, neutralizing, hypersensitivity- or anaphylaxis-triggering, and cross-reactive neutralizing). Such anti-drug antibodies (ADA) may lead to severe and, when not timely and properly treated, potentially lethal clinical consequences, loss of treatment efficacy, and the formation of autoimmunity [4,5,6,7,8]. The frequency of different ADA types and their clinical impact have a reverse relationship, in that binding antibodies occur most frequently and have low clinical impact whereas cross-reacting neutralizing antibodies are rare but have the highest clinical significance.

The immunogenic risk of biotherapeutics and ADA response can be influenced by a multitude of factors. One such factor is the presence of innate immune response modulating impurities (IIRMIs) that might be inadvertently introduced during product manufacturing [4,5]. IIRMIs may have little or no impact on the function of the resulting drug product but may influence the host immune response [4,9,10,11,12,13]. While it is nearly impossible to predict the immunogenicity of a specific biotherapeutic without directly assessing the related immune responses in vivo [3], the presence of IIRMIs contributing to the immunogenicity via priming the immune cells could be identified using in vitro methods detecting innate immunostimulatory responses, including the production of inflammatory cytokines (e.g., IL-1, IFNs, IL-8, TNFα, etc.) and activation of the complement system. Therefore, there is an urgent need in understanding the applicability to, and performance of, in vitro assays in detecting IIRMIs presence in drug products.

Herein, we report the results of an in vitro study analyzing the applicability of several in vitro assays (i.e., complement activation, leukocyte proliferation, and cytokine secretion) to the screening of innate immune responses induced by ten common IIRMIs, including *Bacillus subtilis* flagellin, FSL-1, zymosan, ODN2006, poly(I:C) HMW, poly(I:C) LMW, CLO75, MDP, ODN2216, and *Escherichia coli* O111:B4 LPS, as well as model therapeutic Forteo™ (teriparatide or TP). The selected assays were chosen due to the known roles of the complement system, cytokines, and activated leukocytes in the process of immunogenicity [14]. While these immunostimulatory biomarkers do not directly predict immunogenicity, they serve as important prerequisites to it, which, when monitored in vitro, may allow for the detection of biologically active contaminants contributing to the process of immunogenicity by priming the immune cells [14].

## 2. Results

### 2.1. Initial In Vitro Characterization and Assay Selection

Forteo™ is a peptide-based therapeutic formulation where the active peptide, teriparatide (TP), is produced using recombinant DNA technology. To characterize the whole product, we first established that TP and its corresponding formulation buffer (FB) had no detectable endotoxin and β-glucans that could activate innate immune responses [15,16,17] using a commercial turbidity *Limulus* Amebocyte Lysate (LAL) assay and Factor-C depleted LAL (Glucatell) assay respectively (Table 1 and Table S1).

Detection of impurities in cell-based assays requires cells that are sensitive to the presence of IIRMI and can elicit a quantifiable response. Previous studies have shown that very low levels of impurities that trigger pattern recognition receptors (PRRs) can stimulate a local innate immune response at the site of inoculation and suggested that cell-based assays could be used to detect these types of impurities in the products. Since retaining cell viability throughout the assay is critical, we first determined whether TP would alter cell viability and determined the highest concentration of TP that could be used in a PBMC-based study where the cells were in culture for 24 h. As shown in Figure S1, when PBMC were cultured in the presence of TP at concentrations ranging from 0.025 to 25 μg/mL, the viability of the cells was retained, but higher concentrations of the product reduced cell viability to 60% (Figure S1). Based on this data, the highest non-toxic concentration (25 µg/mL) was chosen as the top concentration to be used for subsequent in vitro experiments, including the assessment of TP and/or IIRMI activation of C3a complement, leukocyte proliferation, and cytokine secretion (Table 1).

Next, we determined whether TP, in concentrations that do not interfere with cell viability, can reduce the response to potential impurities. Using an array of purified TLR agonists at concentrations that are close to those shown to elicit a local innate immune response in vivo, we examined whether the presence of TP in the culture would modulate the response to the PRR-agonists. As shown in Figure S2, while PRR-agonist exposure triggered low levels of leukocyte proliferation in a dose-dependent manner, the response was abrogated in TP-treated cultures (Table 1).

In addition to inducing cell proliferation, the activation of innate immune cells could also induce complement activation. Therefore, we next explored whether TP would activate complement. Treatment with TP resulted in an activation of the complement system as evidenced by an increase in detectable C3a split products; this activation was comparable to that detected in Cremophor-EL and Feraheme-treated plasma samples, used as positive controls (Figure S3). Concentrations of IIRMIs capable of inducing detectable complement activation are typically higher than what may potentially be present in drug products as undesirable contaminants. For example, concentrations of zymosan and lipopolysaccharide (LPS) required to produce detectable complement activation are 10 mg/mL or >500 µg/mL, respectively [18,19]. Therefore, this assay was not selected for subsequent experiments (Table 1).

### 2.2. In Vitro Cytokine Responses to Teriparatide

**Figure 1:**
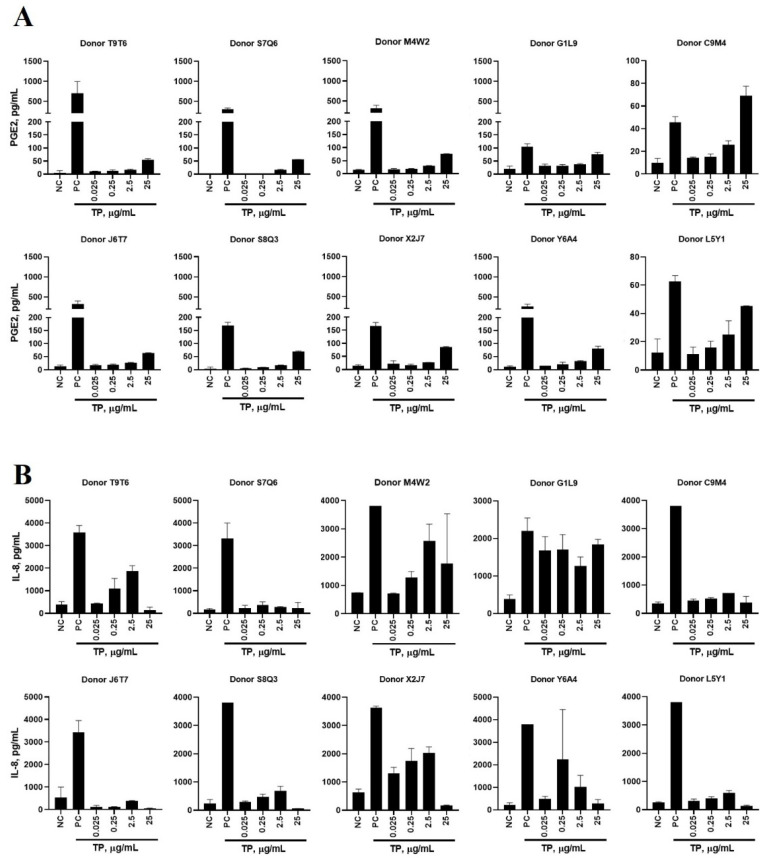
A 16-plex Induction of Prostaglandin-E_2_ and Interleukin-8 by Teriparatide. PBMCs from 10 healthy human donors were treated with 0.025, 0.25, 2.5, and 25 µg/mL teriparatide (TP), compared to a PBS negative control (NC) and LPS/PHA-M/ODN positive control (PC) for 24 h. Supernatants were analyzed for the presence of (**A**) PGE-2 or (**B**) IL-8 by 16-plex multiplex ELISA. Each bar shows mean and standard deviation (N = 2).

PBMCs treated with TP alone noticeably induced PGE-2 and IL-8 production (Figure S4). TP-induced PGE-2 production directly correlated with TP concentrations added to PBMC cultures (Figure 1A). Such correlation for IL-8 induction was only observed in 3 of the 10 tested PBMC cultures (Figure 1B). Cultures from the remaining donors showed increased IL-8 levels at the second-highest concentration (2.5 µg/mL) but not at the highest concentration (25 µg/mL). The reduced levels of IL-8 secreted after incubation with highest concentration of TP (25 µg/mL) suggest a level of PBMC exhaustion resulting from high stimulation over the course of 24 h.

### 2.3. Teriparatide Effects on Cytokine Expression Are Due to the Formulation Buffer (FB)

**Figure 2:**
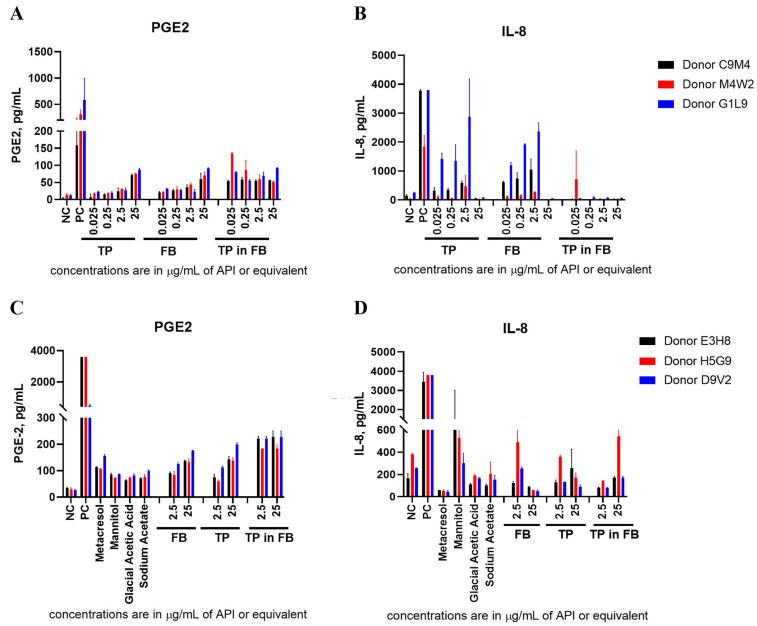
Formulation Buffer is Responsible for Prostaglandin-E_2_ and Interleukin-8 Cytokine Response to Teriparatide. (**A**,**B**) PBMCs from three healthy human donors were used to test teriparatide (TP) at 0.025, 0.25, 2.5, and 25 µg/mL API, diluted in either PBS or Formulation Buffer (FB), compared to complete FB diluted in PBS to achieve the equivalent API concentrations, compared to a PBS negative control (NC) and LPS/PHA-M/ODN positive control (PC). Each bar shows a mean response and a standard deviation (N = 3); (**C**,**D**) PBMCs from another set of three healthy donors were used to test the components of FB (metacresol, mannitol, glacial acetic acid, and sodium acetate) at concentrations equivalent to 25 µg/mL of API in TP, in comparison to complete FB, TP diluted in PBS, and TP diluted in FB. Each bar shows a mean response and a standard deviation (N = 2).

To understand whether the induction of PGE-2 and IL-8 observed in TP-treated cultures (Figure S4) was due to the active pharmaceutical ingredient (API) or FB, we conducted a follow-up experiment in which TP was tested side-by-side with FB at equivalent dilutions that resulted in equivalent concentrations of the FB; these dilutions were performed in PBS. We also performed TP dilutions in the FB and tested them in the same cultures with PBS-diluted FB and TP. The results of this experiment demonstrated that PGE-2 and IL-8 responses to TP were due to the FB (Figure 2A,B and Figure S5).

Next, we hypothesize that metacresol, a preservative of FB, was the cause of the cytokine response to TP, because an earlier study in THP-1 cells reported that this excipient, at a concentration comparable to that present in our cultures (0.2 mg/mL), induced chemokine MCP-1 (but not TNFα, IL-1, or IL-6) [20]. To verify this hypothesis, metacresol and other components of FB (mannitol, glacial acetic acid, and sodium acetate) at concentrations equivalent to that of API in TP were added to PBMC cultures and the supernatants were analyzed for the presence of cytokines (Figure 2C,D and Figure S6). The result of this experiment demonstrated that, in addition to metacresol, all other individual components of the FB contribute to the cytokine response observed with TP. Contrary to our hypothesis about the potential inflammatory nature of metacresol, the highest cytokine response, specifically IL-8, was observed upon application of mannitol (Figure 2D), a response which has previously been reported on in vitro PBMCs and in vivo endothelial cells to deleterious effect [21,22].

### 2.4. In Vitro Cytokine Responses to Individual IIRMIs

**Figure 3:**
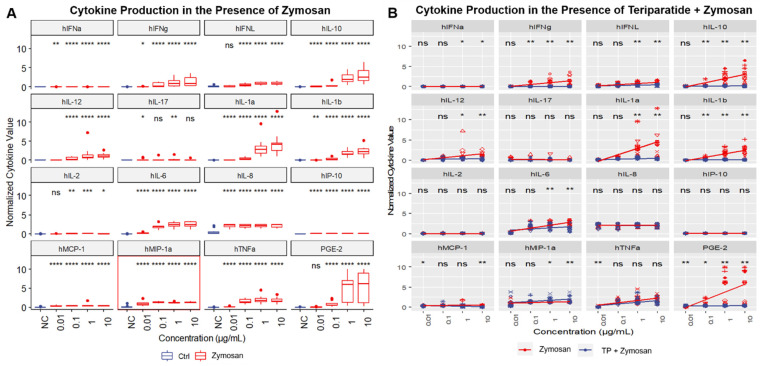
Normalized Cytokine Response to Zymosan and/or Teriparatide and Selection of One Signature Cytokine: PBMCs from 10 healthy human donors were treated with (**A**) zymosan alone or (**B**) zymosan in combination with 25 µg/mL TP for 24 h. Supernatants were analyzed for the presence of cytokines by multiplex ELISA. The signature cytokine (red box) is the one for which the IIRMI concentration, when compared to the PBS negative control (NC), results in a *p* < 0.05. The data for which statistical significance was not observed are marked with ns. Statistical significance is shown with an asterisk as follows: * *p* < 0.05; ** *p* < 0.01; *** *p* < 0.001; and **** *p* < 0.0001. Similar results for the other nine IIRMIs are available in Figures S7 and S8.

**Figure 4:**
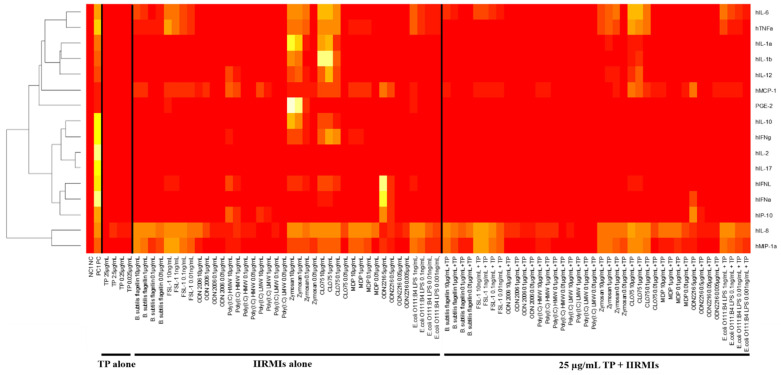
Innate Immune Response Modulating Impurity Treatment and Concentration Patterns via Euclidian Distance and Ward’s Clustering. PBMCs from 10 healthy human donors were treated with various concentrations of IIRMIs, alone and in combination with 25 µg/mL Teriparatide (TP), compared to a PBS negative control (NC) and LPS/PHA-M/ODN positive control (PC), for 24 h. Supernatants were analyzed for the presence of cytokines by multiplex ELISA. Shown is the mean response of normalized values averaged across all donors, clustered based on cytokine response. Dendrograms were created using complete linkage clustering on the Euclidian distance matrices. Similar results for IIRMI clustering available in Figure S9.

Since IIRMIs activated a broad and often overlapping spectrum of cytokines (Figure 3, Figures S7 and S8), we next performed a global analysis using Euclidian distance and Ward’s clustering for the dendrogram and constructed a heatmap of normalized values averaged across all donors and replicates (Figure 4 and Figure S9). This normalization included scaling each cytokine reading across all collected values by dividing each value by that cytokine’s standard deviation obtained across all donors, which brought all cytokines onto roughly the same scale. The benefit of using this approach is that one can compare cytokines directly across all 10 donors, while keeping cytokines with very large values from swamping the comparative global analyses. These analyses revealed a pure red band representing the negative control and a bright yellow vertical band representing the positive control. These analyses also identified groupings of cytokines with similar response patterns across all IIRMI treatments (Figure 4). For example, chemokines IL-8 and MIP-1α showed very similar patterns; PGE-2 showed such a high response to the two higher concentrations of zymosan that it overshadowed the positive control; IL-2 and IL-17 were very similar in that they did not appear to be strongly induced by any IIRMI (Figure 4). Alternatively, these analyses also gave us insight on how various IIRMIs, and their concentrations, clustered with respect to the cytokine response patterns that they induced (Figure S9). These analyses demonstrated that the highest concentrations of each IIRMI often clustered together. For example, zymosan and CLO75 clustered together at the bottom of the heatmap. The analyses also highlighted a group of IIRMIs that seemed to have virtually no cytokine response, including the lowest concentrations of poly(I:C) LMW, ODN2006, poly(I:C) HMW, and ODN2216. Finally, these analyses also highlighted a group of IIRMIs, and their concentrations located in the center of the heat map, that predominantly activated IL-8 (Figure S9).

Further Pearson’s correlation analysis allowed for clustering the cytokine responses based on how well cytokine values correlated across all treatment groups (Figure S10). This analysis revealed that the strongest correlations were between IL-6 and TNFα, IL-8 and MIP-1α, as well as IL-1α, IL-1β, and IL-12 (Figure S10A). We also observed that different concentrations of the same IIRMI tended to correlate well, especially at the higher concentration ranges. For example, higher concentrations of ODN2216, poly(I:C) LMW, and poly(I:C) HMW showed a distinct cluster which was anti-correlated with the higher concentrations of zymosan) and to a lesser degree CLO75 (Figure S10B).

### 2.5. Identification of Signature Cytokines

A two-sided paired Wilcoxon test was used to compare cytokines induced by individual concentrations of IIRMIs with negative control samples pooled across all donors (Figure 3A). For each IIRMI, a signature cytokine was identified by determining the lowest IIRMI concentration which, when compared to the baseline, resulted in both an elevation of the cytokine and the lowest p-value (i.e., at least *p* < 0.05) (Figure 3A, red box). For each IIRMI concentration, if two cytokines achieved a level of significance, the lower (more significant) *p*-value won. Since many test samples, especially negative controls, resulted in cytokine levels below the assay lower limit of detection, we used a non-parametric test for statistical analysis. This approach ranks cytokine significance values rather than the magnitude of cytokine difference. Therefore, the “winning” cytokine was not always the one that appeared the best with regards to mean differences, but rather the one that both had the fewest overlaps between treated samples and controls and was consistent between individual donors (Table 2; Figure S7). Other cytokines with statistically significant elevation above the baseline, at the same IIRMI concentration as the signature cytokine, were also observed (Table 2; Figure S7).

### 2.6. Selection of the Cytokine Panel Specific to Teriparatide and Individual IIRMIs

In order to understand whether the 16-cytokine panel could be narrowed down to three or four cytokines that would be representative of all 10 IIRMIs, we performed additional analysis using the same approach as described above but focused on the top three “winning” cytokines for each IIRMI (Table 3). For this analysis, IIRMIs were grouped based on the intracellular location of their cognate PRRs. Interestingly, all IIRMIs that activate membrane-tethered TLRs consistently induced two cytokines (IL-1α and MIP-1α (Table 3). This finding suggests that any of these two cytokines could be used as a biomarker for the detection of IIRMIs triggering membrane-tethered PRRs. In contrast, no such consistency was observed for IIRMIs that activate endosomal TLRs. Therefore, a combination of cytokines MCP-1 and IL-8 or MCP-1 and IL-6 would be required to suggest the presence of IIRMIs triggering endosomal TLRs (Table 3). One of the following cytokines—IL-6, IL-8, or IP-10—could be used to suggest the presence of IIRMIs triggering cytosolic PRRs (Table 3).

**Figure 5:**
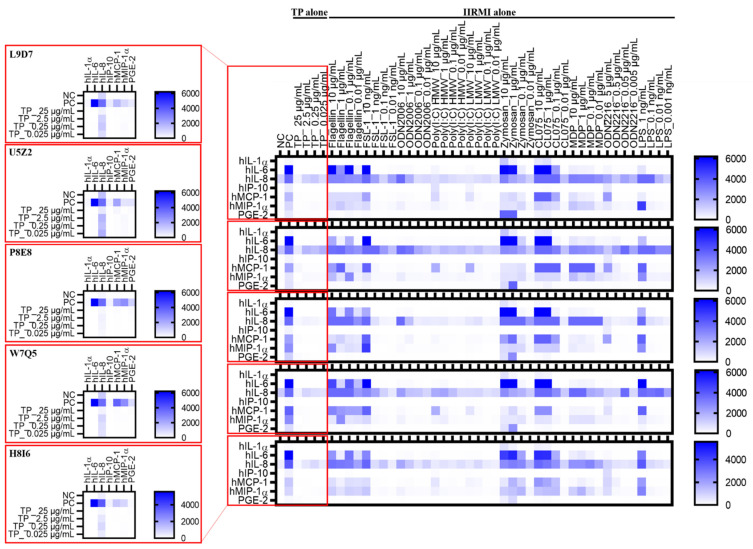
Seven-plex Induction of Cytokines in PBMCs. PBMCs collected from 10 healthy human donors were treated with 0.025, 0.25, 2.5, and 25 µg/mL TP (red box) or IIRMIs alone, compared to a PBS negative control (NC) and LPS/PHA-M/ODN positive control (PC), for 24 h. Supernatants were analyzed for the presence of cytokines by multiplex ELISA. Shown is the mean response (N = 2). Shown here are the data generated using PBMC cultures of five representative donors. The data generated using PBMCs of the remaining five donors are presented in Figure S11. Normalized data for each treatment set in all ten donors are also presented in Figure S12.

**Figure 6:**
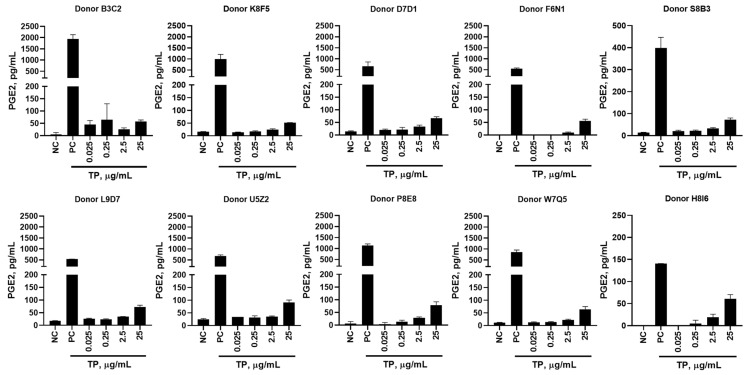
Seven-plex Induction of Prostaglandin-E_2_ by Teriparatide. PBMCs from 10 healthy human donors were treated with 0.025, 0.25, 2.5, and 25 µg/mL teriparatide (TP), compared to a PBS negative control (NC) and LPS/PHA-M/ODN positive control (PC), for 24 h. Supernatants were analyzed for the presence of PGE-2 by 7-plex multiplex ELISA. Each bar shows mean and standard deviation (N = 2).

For the subsequent experiments, we focused on a seven-cytokine panel which includes a combination of the following signature cytokines (IL-1α, MIP-1α, IP-10, MCP-1, IL-6, and IL-8) representing all tested IIRMIs, and one cytokine (PGE-2) representing the response to TP (Figure 5, Figures S11 and S12). Interestingly, the majority of IIRMIs that activate membrane-tethered TLRs, induced MCP-1 production, with the remaining IIRMI, zymosan, instead inducing MIP-1α expression rather than the expected IL-1α. For the cytosolic PRRs, the overwhelming response was IL-8 expression. For the endosomal PRRs, we again observed that there was no cytokine consistency, with the highest cytokine expression covering MIP-1α, IL-8, and IL-6, with two of the five IIRMIs inducing high levels of MCP-1. For the majority of the IIRMIs, there was little or no IL-1α, IP-10, or PGE-2 expression detected (Figure 5, Figures S11 and S12). However, PGE-2 production had a dose dependent response when PBMCs were treated with increasing concentrations of TP alone (Figure 6). This effect was previously observed (Figure 1A) indicating that PGE-2 is still a hallmark cytokine for tracking TP immunostimulatory activity.

### 2.7. Teriparatide Affects Expression of IIRMI-Induced Cytokines

The presence of TP in cell cultures affected the induction of cytokines by individual IIRMIs (Table 4; Figure S8). Euclidian distance and Ward’s clustering analysis demonstrated that the patterns for chemokines IL-8 and MIP-1α did not change with the addition of TP (right half of the plot) (Figure 4). In contrast, the group of IL-1β, IL-1α, and IL-12, which showed strong responses to the higher concentration of zymosan and CLO75, was strongly inhibited by the addition of TP. The loss of response with TP was also seen at the highest concentration of IIRMI for cytokines IFNλ and IFNα. PGE-2 induced by two higher concentrations of zymosan was also lost with the addition of TP (Figure 4).

### 2.8. Teriparatide Effects on IIRMI-Induced Cytokines Are Due to the Formulation Buffer (FB)

To understand whether the suppression of IIRMI-induced cytokines by TP was due to the API or FB, we conducted a follow-up experiment in which four concentrations of TP were tested side-by-side with the second highest concentration of IIRMIs alone, as well as IIRMIs in combination with either 25 µg/mL TP or equivalent 25 µg/mL FB. The results of this experiment demonstrated that changes in the expression of IIRMI-induced cytokines by TP were due to the FB (Figure S13).

### 2.9. Donor’s Genetic Background Determines the Magnitude of Cytokine Response to IIRMIs

We observed that PBMCs from some donors demonstrated more robust (i.e., higher magnitude) responses to TP than cultures from other healthy donors (Figure S4). To understand whether such differences were due to the genetic background of the PBMC donor or variability in the day-to-day handling of donor’s blood and PBMCs, we recalled one highest responder (donor G9L1) and two average responders (donors M4W2 and C9M4) for the second time, repeated the TP treatments, and compared the results between two experiments. The results were consistent between the two experiments despite some variability in the individual cytokine levels observed in all donors (Figure S14).

### 2.10. Influence of Assay Logistics on Cytokine Responses to TP and IIRMIs

**Figure 7:**
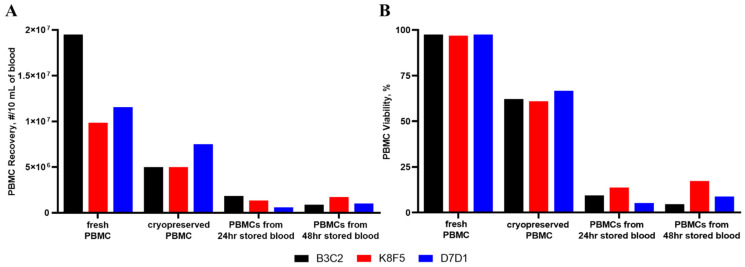
The Effect of Storage Conditions on PBMC Viability and Cell Recovery. To simulate various handling and storage conditions used in research, PBMCs from three healthy human donors were examined after fresh isolation, cryopreservation, and isolation from refrigerated blood (24 h or 48 h). Cell viability was then assessed using AO/PI. (**A**) Number of PBMCs recovered under the various storage/handling conditions. (**B**) Viability of stored PBMCs compared to their freshly isolated PBMC counterparts. Each bar shows the mean result and standard deviation (N = 3).

**Figure 8:**
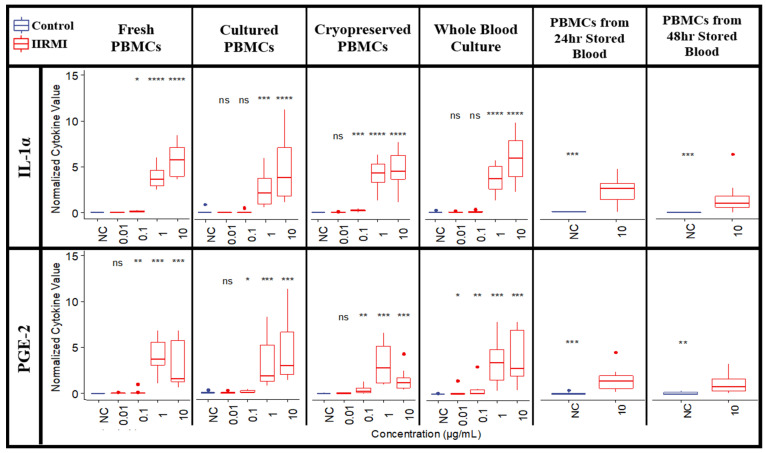
IL-1α and PGE-2 Responses to Zymosan are Affected by PBMC and Blood Handling Conditions. PBMCs from 10 healthy human donors were exposed to various common laboratory handling conditions (isolated from fresh blood, cultured for 24 h, cryopreserved, isolated from blood refrigerated for 24 h or 48 h, and whole blood cultures) before being treated with IIRMIs for 24 h. Supernatants were analyzed for the presence of cytokines. Shown are the mean cytokine responses to zymosan (red), compared to a PBS negative control (NC, blue). The data for which statistical significance was not observed are marked with ns. Statistical significance is shown with an asterisk as follows: * *p* < 0.05; ** *p* < 0.01; *** *p* < 0.001; and **** *p* < 0.0001. Additional results, including zymosan-induced levels of the remaining five cytokines and the cytokine responses for the other nine IIRMIs, are available in Figure 5 (fresh PBMCs) and Figure S15 (all other experimental conditions).

We further examined the influence of blood handling and storage conditions on resultant cytokine responses to IIRMIs or TP. Blood from ten healthy donors was separated into six treatment groups: freshly isolated and freshly treated PBMCs; freshly isolated PBMCs cultured for 24 h prior to the treatment; freshly isolated and cryopreserved PBMCs; PBMCs isolated from blood refrigerated for 24 h or 48 h before PBMC isolation; and whole blood cultures. All groups were then dosed with IIRMIs or TP for 24 h. We then measured PBMC recovery and viability for these treatment groups (Figure 7) as well as the levels of our seven key cytokines, IL-1α, MIP-1α, IP-10, MCP-1, IL-6, and IL-8 (Figure 5, Figure 8, Figures S11, S12 and S15).

Compared to freshly isolated PBMCs, PBMC viability is reduced to approximately 63% after 48 h of cryopreservation, as compared to the very low viability (~10%) of PBMCs isolated from anti-coagulated blood after refrigeration storage for 24 h or 48 h (Figure 7B). Due to the loss of 90% of usable PBMCs from the stored blood samples, we were only able to treat the remaining cells with a limited selection of IIRMIs for comparison to the other treatment/storage conditions. In addition, this loss of available cells can potentially skew the resultant cytokine production (Figure 7A).

As previously discussed, there was very little general expression of IL-1α, IP-10, or PGE-2 detected even for freshly isolated PBMCs (Figure 5, Figure 8, Figures S11, S12 and S15). Interestingly, the highest levels of IL-1α and PGE-2 were observed after zymosan stimulation in whole blood cultures, indicating that other components of blood may be responsible for increasing the levels of these cytokines.

For the other four cytokines (MCP-1, MIP-1α, IL-8, and IL-6), cultured PBMCs and cryopreserved PBMCs had similar but reduced levels of cytokines compared to freshly isolated PBMCs. Cytokines from refrigerated blood further reduced cytokine levels, even at the highest IIRMI concentrations. This was especially true for IL-6, which were reduced to almost nothing even in the presence of strong LPS or zymosan stimulation (Figure 5, Figure 8, Figures S11, S12 and S15).

## 3. Discussion

Based on the results of these initial characterization studies (Table 1), complement activation and leukocyte-proliferation assays were not chosen for subsequent studies as they cannot adequately detect potential differences in IIRMI contamination between different batches of product. These assays, however, could be helpful in studies investigating different formulations of the same API. Examples may include when a product is reformulated, or when a generic or follow-on product elects to have differences in formulation compared to an innovator (reference) product. Therefore, we focused the rest of the study on the cytokine secretion by PBMC after in vitro exposure to TP and formulations containing IIRMIs.

Our study suggested that PGE-2 could be used as a signature cytokine for tracking TP induction of innate immune responses (Figure 1A and Figure 6). We further found that this response is mediated by the FB rather than API. Further investigation found that, unlike our hypothesis about the influence of metacresol, all the FB ingredients contributed to the resultant cytokine response (Figure 2A,C; Figures S5 and S6).

IIRMIs activated a broad and often overlapping spectrum of cytokines (Figure 3, Figures S4, S7 and S8). This finding is consistent with the current literature about PRRs and their cognate ligands [23,24,25]. Using Euclidian distance and Ward’s clustering analyses, we obtained insight on the patterns of IIRMI stimulation and the resultant induced cytokine responses. From these results, we identified groupings of cytokines with similar response patterns across all IIRMI treatments (Figure 4), as well as several cytokines which did not appear to be strongly induced by any IIRMI. These analyses also demonstrated that the highest concentrations of each IIRMI often clustered together (Figure S9).

Further Pearson’s correlation analysis allowed for clustering the cytokine responses based on how well cytokine values correlated across all treatment groups and donors (Figure S10A). Strong correlations patterns identified during these analyses were consistent with the currently available literature about the function of these cytokines and the cells that produce them. Specifically, IL-6 and TNFα are produced by monocytes and T-cells, and are responsible for pyrogenicity; IL-8 and MIP-1α are chemokines produced by monocytes and responsible for neutrophils and mixed leukocyte recruitment; IL-1α, IL-1β, and IL-12 are produced by monocytes and DCs and are responsible for the inflammation, fever, and activation of specific subsets of lymphocytes (i.e., IL-1β promotes T_H_17 differentiation, whereas IL-12 supports T_H_1 differentiation, NK and T-cell activation to increase IFNγ synthesis and increased cytotoxicity); in addition, IL-1α is a danger signal that indicates damaging effects of IIRMIs that induce its secretion [14]. According to our expectations from the global heatmaps (Figure 4 and Figure S9), Pearson’s correlation between the different concentrations of the same IIRMI was consistent with the current knowledge about type and intracellular localization of PRRs stimulated by these IIRMIs (Figure S10B). Specifically, ODN2216, poly(I:C) LMW, and poly(I:C) HMW activate endosomal TLRs (TLR9 and TLR3), whereas zymosan triggers membrane-tethered PRRs (TLR2 and Dectin1) [25,26]. In contrast, CLO75, which showed a lower degree of anti-correlation, is also located in the endosome but is specific to a different PRR (TLR8) [27].

To understand whether our 16-cytokine panel could be narrowed down to three or four cytokines that would be representative of all 10 IIRMIs, we examined the top three “winning” cytokines identified for each IIRMI (Table 3). Due to the overlapping nature of the induced cytokines, we identified two possible panels of three cytokines which would provide at least one positive result for all 10 IIRMIs and potentially could be used by users who do not have access to more than a 3- or 4-plex cytokine detection panel. These panels include the following markers: 1) IL-1α (or MIP-1α), IP-10, and IL-8; or 2) IL-1α (or MIP-1α), MCP-1 and IL-8 (or IL-6).

The results from the subsequent 7-plex panel containing IL-1α, MIP-1α, IP-10, MCP-1, IL-6, IL-8, and PGE-2 (Figure 5, Figures S11 and S12), suggest that our initial 16-cytokine panel can be reduced to a four-cytokine panel, specifically containing MCP-1, MIP-1α, IL-8, and IL-6, which would be representative of all 10 IIRMIs, which can further be expanded to a five-cytokine TP-specific panel, which includes the TP-signature cytokine, PGE-2, in addition to the four IIRMI-specific cytokines.

TP did not significantly increase the levels of cytokines induced by IIRMIs. TP was, instead, found to decrease the levels of most IIRMI-induced cytokines (Figure 4). Reduced levels of IIRMI-induced cytokine responses in the presence of TP were also the result of the FB rather than the API (Figure S13). Collectively, this finding and the data demonstrating the induction of TP signature cytokine PGE-2 by the FB suggests that the assessment of potential IIRMI contamination of the API could be more informative for comparison of RLD and generic formulations. This data also suggests that a change in the formulation buffer may result in a change in the signature cytokine of the whole product.

The more robust cytokine responses to TP demonstrated by some donors suggests that day-to-day variability in phlebotomy and handling of whole blood and PBMCs may result in quantitative differences (i.e., influence the magnitude of the responses) but would not change the overall qualitative trends and resultant conclusions of the study. However, the genetic background of donors that donate their blood for in vitro experiments does appear to be an important factor in qualitative determination of the PBMC response to individual IIRMIs (Figure S14).

Overall, the PBMC handling and blood storage conditions have a significant effect on the detectable levels of cytokines, with freshly isolated PBMCs being the most preferred condition since it allows for more adequate detection of cytokines as a result of innate immunity activation (Figure 5, Figure 8, Figures S11, S12 and S15).

## 4. Materials and Methods

### 4.1. Materials

Feraheme (FH) (AMAG Pharmaceuticals, Waltham, MA) and Forteo™ (teriparatide, TP) (Eli Lilly, Indianapolis, IN, USA), were obtained from NIH Pharmacy. All *Limulus* amebocyte lysate (LAL) reagents, LAL grade (endotoxin free) water, Glucatell kits, Glucashield buffer, and *E. coli* lipopolysaccharide (LPS) were from Associates of Cape Cod (East Falmouth, MA, USA). Veronal Buffer was obtained from Boston BioProducts (Ashland, MA, USA). Phosphate Buffered Saline (PBS), RPMI-1640 media, fetal bovine serum (FBS), penicillin and streptomycin solution, L-glutamine, Ficoll-Paque Premium was from GE Life Sciences (Marlborough, MA, USA). Hank’s balanced salt solution (HBSS) was from Gibco (Gaithersburg, MD). All IIRMIs—*B. subtilis* flagellin, FSL-1, ODN2006 Class B, poly(I:C) HMW, poly(I:C) LMW, zymosan, CLO75, MDP, ODN2216, and *E. coli* O111:B4 LPS—were from Invivogen (San Diego, CA, USA). Acridine orange (AO)/propidium iodide (PI) staining solution were purchased from Nexcelom Bioscience (Lawrence, MA, USA). The 16-plex and 7-plex cytokine multiplex kits were supplied by Quansys Biosciences (Logan, UT, USA). Cobra venom factor (CVF), Heat Aggregated Gamma Globulins (HAGG), and MicroVue EIA kits were purchased from Quidel Corporation (San Diego, CA, USA). Glacial acetic acid, sodium acetate, mannitol, sodium hydroxide (NaOH), hydrochloric acid (HCl), MTT (3-(4,5-dimethyl-2-thiazolyl)-2,5-diphenyl-2H-tetrazolium bromide), glycine, sodium chloride, dimethyl sulfoxide (DMSO), Phytohemagglutinin (PHA-M), and Cremophor (Cre) were purchased from Sigma-Aldrich (Burlington, MA, USA). Metacresol was from USP (Frederick, MD, USA).

### 4.2. Innate Immune Response Modulating Impurities

Ten model innate immune response modulating impurities (IIRMIs) were tested at four concentrations (Table 5) either alone or in combination with teriparatide (TP). Eight IIRMIs (*B. subtilis* flagellin, FSL-1, zymosan, ODN2006, poly(I:C) HMW, poly(I:C) LMW, CLO75, and MDP) were selected based on preliminary studies in HEK-TLR reporter cells [9,10]; two other IIRMIs (ODN2216 and *E. coli* O111:B4 LPS) were selected based on the Nanotechnology Characterization Laboratory (NCL) (https://ncl.cancer.gov/, accessed on 15 October 2020) prior experience using them as immunological assay cascade positive controls. Taken together, these ten IIRMIs bind Dectin 1, TLRs 2, 3, 4, 5, 6, 8, 9, and NOD2, as summarized in Table 5.

### 4.3. Endotoxin Detection

Endotoxin levels were evaluated using the kinetic turbidity *Limulus* Amebocyte Lysate (LAL) Assay according to NCL protocol STE-1.2 [28,29]. Briefly, 100 µL of TP (at 250 µg/mL) and the equivalent amount of its formulation buffer (FB) were each mixed with 100 µL of LAL reagent in a glass tube, then measured via spectrophotometer at 660 nm for at least 7200 sec for appropriate development. Using a standard curve prepared with Control Standard Endotoxin of known potency, we calculated the concentration of endotoxin present in the TP and FB solutions.

### 4.4. β-Glucan Detection

Levels of β-glucans were evaluated using Glucatell^®^ kit as detailed in NCL protocol STE-4 [15,30]. Briefly, 50 µL of TP (at 250 µg/mL) and the equivalent amount of its formulation buffer (FB) were each mixed with 50 µL of Glucatell reagent in a 96-well plate and incubated at 37 °C. The reaction was stopped through the addition of 50 µL of 1N HCl-sodium nitrite solution, 50 μL of ammonium sulfamate solution, and then 50 µL of NEDA solution to each well. Color development was immediately observed and measured at 540–550 nm using a spectrophotometer. Using a β-(1,3)-D-glucan standard curve, we calculated the concentration of β-(1,3)-D-glucan present in the TP and FB solutions.

### 4.5. Donor Blood

Blood from healthy human donors was collected in vacutainers containing either Li-heparin or K_2_-EDTA (BD Biosciences, San Jose, CA, USA) under the NCI-Frederick protocol OH9-C-N046. At the time of blood collection, donors were not on any medications and have never been exposed to the model Forteo™ teriparatide formulation.

### 4.6. Peripheral Blood Mononuclear Cell (PBMC) Isolation and Culture

Fresh donor blood anti-coagulated with Li-heparin was mixed with an equal volume of room-temperature PBS. The blood/PBS mixture was then slowly layered on top of Ficoll-Paque solution in a 4:3 ratio. The sample was centrifuged for 30 min at 900*× g*, 18–20 °C, without brake. After centrifugation, the upper layer containing plasma and platelets was removed and discarded. The mononuclear cell layer was isolated and washed using an excess (approximately three times volume) of Hank’s Balanced Salt Solution (HBSS) and centrifuged for 10–15 min at 400*× g*, 18–20 °C. After washing, the supernatant was discarded, and the wash step was repeated once more. The remaining mononuclear cells were then resuspended in complete RPMI-1640 medium, containing 10% FBS (heat inactivated), 2 mM L-glutamine, 100 U/mL penicillin, and 100 µg/mL streptomycin. Cell viability was then determined using the acridine orange (AO)/propidium iodide (PI) dual-fluorescence viability method, in which an equal volume of staining solution, containing AO (live cells, green) and PI (dead cells, red) was added to cells and analyzed in <60 s using a fluorescent Cellometer instrument. The details of the protocols are publicly available through NCL protocol ITA-10 and were previously described [31,32].

### 4.7. PBMC Cryopreservation

Isolated PBMCs were resuspended at a concentration of 5–7.5 × 10^6^ cells/mL in freezing media (10% DMSO in FBS), placed into cryopreservation tubes, and stored in a freezing container containing isopropanol for controlled freezing at −80 °C.

### 4.8. Whole Blood Cell (WBC) Culture

Fresh donor blood anti-coagulated with Li-heparin was mixed 1:4 with room-temperature PBS (e.g., 10 mL blood added to 30 mL PBS). The blood/PBS mixture was then added directly to 96-well plate for treatment and culture at 37 °C. The details of the protocols are publicly available through NCL protocol ITA-10 and were previously described [31,32].

### 4.9. Teriparatide Cytotoxicity Analysis

PBMCs in complete 1640-RPMI were incubated with 0–50 µg/mL teriparatide (TP) for 24 h. Cell viability was then determined using the AO/PI staining method [33].

### 4.10. Leukocyte Proliferation

PBMCs were cultured at in the presence of controls, 0.025–25 µg/mL TP, four concentrations of IIRMIs (Table 5), or four concentrations of IIRMI + 25 µg/mL TP for 72 h. The proliferation of leukocytes was determined according to NCL protocol ITA-6 [34].

### 4.11. Complement Activation

These experiments were conducted according to NCL protocol ITA5.2 [35]. Briefly, K_2_-EDTA plasma from individual donors was pooled and incubated with controls or 0.025–83.3 µg/mL TP, and veronal buffer for 30 min at 37 °C. Following incubation, the samples were analyzed for the presence of complement split product C3a using a commercial multiplex ELISA kit. In this experiment, Cobra venom factor (CVF) and Heat Aggregated Gamma Globulins (HAGG) were used as the assay positive controls (PC). Cremophor (Cre) and Feraheme (FH) were included as additional controls as they are known to cause complement-mediated toxicity in sensitive patients [36,37,38,39].

### 4.12. Cytokine Production

These experiments followed NCL protocol ITA-10 [31,32]. PBMCs were cultured at in the presence of PBC negative control, LPS/PHA-M/ODN positive control, 0.025–25 µg/mL TP, four concentrations of IIRMIs (Table 5), or four concentrations of IIRMI + 25 µg/mL TP for 24 h in a humidified 37 °C, 5% CO_2_ incubator. After incubation, the plates were centrifuged for 5 min at 700× *g* to pellet the PBMCs. The supernatants were collected for cytokine analysis using custom 16-plex or 7-plex multiplex plates from Quansys Biosciences (Logan, UT, USA). The cytokines present in the multiplex panel included type I interferon (IFNα), type II interferon (IFNγ), type III interferon (IFNλ), interleukins (IL-1α, IL-1β, IL-2, IL-6, IL-8, IL-10, IL-12, IL-17), interferon-gamma inducible protein (IP-10), tumor necrosis factor alpha (TNFα), prostaglandin-E_2_ (PGE-2), macrophage inflammatory protein (MIP-1α), and monocyte chemoattractant protein (MCP-1). Cytokine levels were each quantified against a standard curve of calibrator controls (provided in the Quansys kit).

### 4.13. Statistical Analysis

All experiments were performed with at least two independent samples, tested in duplicate (%CV < 25). Unless otherwise stated, results show the mean and standard deviation generated from these independent samples. For the cytokine multiplex assay, the analysis was performed using custom R scripts. Cytokine concentration values above the detection limit (“ADL”) were set to the upper detection limit, and values below the detection limit (“BDL”) were set to zero. Statistical analysis of cytokine data was performed using normalized values. The normalization included scaling each cytokine reading across all collected values by dividing each value by that cytokine’s standard deviation obtained across all donors. The normalization brought all cytokines onto roughly the same scale. The benefit of using this approach is that one can compare cytokines directly on graphs across 10 donors, and it keeps cytokines with very large values from swamping global analyses. As an initial quality control (QC) step, we looked at the negative control (NC) vs. positive control (PC) values for each cytokine using the Wilcoxon non-parametric test on replicate-averaged cytokine-normalized values. All cytokines showed significantly higher PC than NC values. Additionally, we looked at the correlation between pairs of replicate runs (a vs. b for each treatment) and observed a good correlation for most pairs. Unless otherwise noted, comparisons between cytokine levels were made on normalized values using two-sided Wilcoxon tests.

## 5. Conclusions

Cytokine secretion by human PBMCs may be used to assess the innate immune responses to IIRMIs, formulation components, and whole products containing peptide and protein therapeutics. While the whole product needs to be analyzed, the results of our study emphasize that the components of FB are not immunologically inert and can contribute to both the cytokine stimulation by the whole product and inhibition of the IIRMI-mediated cytokines. Statistical analysis helps to identify signature cytokines and select cytokine panel appropriate for the given peptide drug product and any prospective generics and biosimilars. It is expected that signature cytokines maybe different between different products due to differences in formulation components, potential IIRMI contamination, immunological properties of API, and interactions among them, which collectively may lead to both quantitative and qualitative differences. Importantly, the logistics of blood storage and handling may influence the results, and, therefore, should be carefully investigated during assay validation phase.

## Figures and Tables

**Table 1:** Initial Characterization of Teriparatide. Teriparatide (TP) purity and capability of triggering innate immunity activation in vitro, either due to the presence of innate immune response modulating impurities (IIRMIs) in the drug formulation or due to the presence of the drug itself, was assessed through the following assays. Results were below the level of detection, so these assays were not used for future TP immunity experiments. LAL = *Limulus* Amoebocyte Lysate Assay; LLOQ = lower limit of quantification; STE = Sterility Endotoxin assay; ITA = Immuno-Toxicity Assay; CBA = Cell Based Assay; ELISA = Enzyme-Linked Immuno-Sorbent Assay; AO = Acridine Orange; PI = Propidium Iodine.

Purpose	Assay Type (NCL Protocol)	Main Findings
Endotoxin Detection	LAL (STE-1.2)	Endotoxin contamination is below the assay LLOQ
β-Glucan Detection	Glucatell (STE-4)	β-glucan contamination is below the assay LLOQ
Cell Viability/Teriparatide Cytotoxicity	AO/PI staining	>85% viability for TP <25 μg/mL ~60% viability for 50 μg/mL TP 25 μg/mL TP chosen for future experiments
Leukocyte Proliferation	CBA (ITA-6)	TP did not induce leukocyte proliferation IIRMIs induced low levels of leukocyte proliferation TP suppressed IIRMI-induced leukocyte proliferation The assay is not chosen for future studies
Complement Activation	ELISA (ITA-5.2)	TP resulted in complement activation Levels of IIRMIs contamination in drug product are insufficient for the complement activation The assay is not chosen for future studies

**Table 2:** Cytokines Induced by Innate Immune Response Modulating Impurities. Individual IIRMIs, their cognate pattern recognition receptors (PRRs), and signature cytokines detected after treatment with IIRMI are summarized. Using a two-sided Wilcoxon test, a signature cytokine was identified for each IIRMI by determining the lowest IIRMI concentration, which, when compared to the baseline, resulted in an elevation of the cytokine, and had the lowest ranking *p*-value (i.e., at least *p* < 0.05). IIRMI = innate immune response modulating impurities; TLR = Toll-Like Receptor; IL = interleukin; IFN = interferon; MCP = monocyte chemoattractant protein; MIP = macrophage inflammatory protein; NOD = nucleotide-binding oligomerization domain; TNF = tumor necrosis factor; PGE = prostaglandin; LPS = lipopolysaccharide; CLO = thiazoloquinolone derivative; MDP = muramyldipeptide; ODN = oligo deoxyribonucleotide; LMW = low molecular weight; HMW = high molecular weight; FSL = Pam2CGDPKHPKSF, a synthetic lipopeptide derived from *Mycoplasma salivarium*.

IIRMI	PRR	Signature Cytokine	Lowest Conc. of IIRMI at Which Signature Cytokine Is Detected	Other Cytokines Statistically Higher than the Baseline at the Lowest IIRMI Conc. that Induced Signature Cytokine
*B. subtilis* flagellin	TLR5	IL-1β	0.01 µg/mL	IFNα, IL-10, IL-1α, IL-2, IL-6, IL-8, IL-1, MCP-1, MIP-1α, TNFα
FSL-1	TLR2/TLR6	IL-1α	10 pg/mL	IFNα, IFNγ, IFNλ, IL-10, IL-12, IL-1b, IL-2, IL-6, IL-8, IP-10, MCP-1, MIP-1α, TNFα, PGE-2
ODN2006 Class B	TLR9	IFNα	1 µg/mL	IFNγ, IL-1α, IL-10, IL-2, IL-6, IL-8, IP-10, MCP-1, MIP-1α, TNFα
Poly(I:C) HMW	TLR3	IP-10	0.1 µg/mL	IFNα, IFNγ, IFNλ, IL-10, IL-12, IL-1α, IL-2, IL-6, IL-8, IP-10, MCP-1, MIP-1α, TNFα
Poly(I:C) LMW	TLR3	MCP-1	1 µg/mL	IFNγ, IL-12, IL-6, IP-10, MIP-1α
Zymosan	TLR2/Dectin 1	MIP-1α	0.01 µg/mL	IFNα, IFNγ, IFNλ, IL-10, IL-12, IL-17, IL-1α, IL-1β, IL-6, IL-8, IP-10, MCP-1, TNFα
CLO75	TLR8	IL-10	0.01 µg/mL	IL-1α, IL-1β, IL-2, IL-6, IL-8, IP-10, MCP-1, MIP-1α, TNFα
MDP	NOD2	IL-8	0.01 µg/mL	IFNα, IL-10, IL-12, IL-6, IP-10, MCP-1, MIP-1α, TNFα
ODN2216	TLR9	IL-6	0.005 µg/mL	IL-6, IL-8
*E. coli* O111:B4 LPS	TLR4	IL-1α	1 pg/mL	IFNα, IFNγ, IL-10, IL-12, IL-1β, IL-2, IL-6, IL-8, IP-10, MCP-1, MIP-1α, TNFα, PGE-2

**Table 3:** Selection of three signature cytokines induced by individual Innate Immune Response Modulating Impurities. A two-sided unpaired Wilcoxon test was used to select the top three cytokines for each IIRMI, which had consistent responses between all donors and the lowest *p*-value. Starting with the lowest concentration for each IIRMI, if three cytokines did not achieve significance of *p* ≤ 0.05, the next highest concentration was evaluated until three cytokines were chosen. If more than three cytokines achieved *p* ≤ 0.05 at the selected concentration, the three with the lowest (most significant) p-values were selected. The top three cytokines selected for any IIRMI are shown as “TRUE” while the remaining less significant cytokines are shown as “FALSE”. IIRMIs are grouped based on the intracellular localization of their cognate pattern-recognition receptors (PRRs) and color-coded as follows: BLUE-cellular membrane, RED-endosome, GREEN-cytosol. TRUE values in each group are highlighted in bold and the same color code as that used for corresponding IIRMIs.

IIRMI	IFNα	IFNγ	IFNλ	IL-10	IL-12	IL-17	IL-1α	IL-1β	IL-2	IL-6	IL-8	IP-10	MCP-1	MIP-1α	TNFα	PGE-2
*B. subtilis* flagellin	FALSE	FALSE	FALSE	FALSE	FALSE	FALSE	TRUE	TRUE	FALSE	FALSE	FALSE	FALSE	FALSE	TRUE	FALSE	FALSE
FSL-1	FALSE	FALSE	FALSE	FALSE	FALSE	FALSE	TRUE	TRUE	FALSE	FALSE	FALSE	FALSE	FALSE	TRUE	FALSE	FALSE
Zymosan	FALSE	FALSE	FALSE	FALSE	FALSE	FALSE	TRUE	FALSE	FALSE	FALSE	FALSE	FALSE	TRUE	TRUE	FALSE	FALSE
*E. coli* O111:B4 LPS	FALSE	FALSE	FALSE	FALSE	FALSE	FALSE	TRUE	TRUE	FALSE	FALSE	FALSE	FALSE	FALSE	TRUE	FALSE	FALSE
ODN2006	TRUE	FALSE	FALSE	FALSE	FALSE	FALSE	FALSE	FALSE	FALSE	FALSE	TRUE	FALSE	TRUE	FALSE	FALSE	FALSE
Poly(I:C) HMW	FALSE	FALSE	FALSE	FALSE	FALSE	FALSE	FALSE	FALSE	FALSE	TRUE	FALSE	TRUE	TRUE	FALSE	FALSE	FALSE
Poly(I:C) LMW	FALSE	TRUE	FALSE	FALSE	FALSE	FALSE	FALSE	FALSE	FALSE	FALSE	FALSE	TRUE	TRUE	FALSE	FALSE	FALSE
CLO75	FALSE	FALSE	FALSE	TRUE	FALSE	FALSE	FALSE	FALSE	FALSE	TRUE	TRUE	FALSE	FALSE	FALSE	FALSE	FALSE
ODN2216	TRUE	FALSE	FALSE	FALSE	FALSE	FALSE	FALSE	FALSE	FALSE	FALSE	FALSE	TRUE	TRUE	FALSE	FALSE	FALSE
MDP	FALSE	FALSE	FALSE	FALSE	FALSE	FALSE	FALSE	FALSE	FALSE	TRUE	TRUE	TRUE	FALSE	FALSE	FALSE	FALSE

**Table 4:** Teriparatide Affects Cytokines Induced by Innate Immune Response Modulating Impurities (IIRMIs). Individual IIRMIs and IIRMI-triggered cytokines in which expression is affected by the presence of 25 µg/mL of teriparatide (TP) are summarized in the table. In the presence of TP, all cytokines shown in the table are inhibited, except for the cytokines highlighted with an asterisk (*); levels of these cytokines are higher in the presence of TP. Statistical analysis included a two-sided Wilcoxon test.

IIRMI	IIRMI-Induced Cytokines Affected by TP
*B. subtilis* flagellin	IFNα, IL-1α, IL-1β, IL-6, MIP-1α, TNFα, PGE-2 *
FSL-1	IFNα, IFNγ, IFNλ, IP-10, IL-1α, IL-1β, IL-2, IL-6, TNFα
ODN2006 Class B	IFNα, IP-10, TNFα, PGE-2 *
Poly(I:C) HMW	IFNγ, IFNλ, IL-12, IP-10, MIP-1α, TNFα, PGE-2 *
Poly(I:C) LMW	IFNγ, IP-10, MCP-1, MIP-1α, TNFα, PGE-2 *
Zymosan	IFNα, IFNγ, IFNλ, IL-10, IL-12, IL-1α, IL-1β, IL-6, MCP-1, MIP-1α *, PGE-2
CLO75	IFNα, IFNγ, IFNλ, IL-10, IL-1α, IL-1β, IP-10, PGE-2 *
MDP	IL-1α, IL-1β, MIP-1α, TNFα, PGE-2 *
ODN2216	IFNα, IFNγ, IFNλ, IL-1α, MIP-1α, TNFα, PGE-2 *
*E. coli* O111:B4 LPS	IFNγ, IFNλ, IL-1β

**Table 5:** Innate Immune Response Modulating Impurities used in the present study. IIRMIs and their final concentrations tested in vitro are summarized. LPS = lipopolysaccharide; CLO = thiazoloquinolone derivative; MDP = muramyldipeptide; ODN = oligo deoxyribonucleotide; LMW = low molecular weight; HMW = high molecular weight; FSL = Pam2CGDPKHPKSF, a synthetic lipopeptide derived from *Mycoplasma salivarium*.

Reagent	PRR	Final Concentrations per mL
*B. subtilis* flagellin	TLR5	10 µg, 1 µg, 100 ng, 10 ng
FSL-1	TLR2/TLR6	10 ng, 1 ng, 100 pg, 10 pg
ODN2006 Class B	TLR9	10 µg, 1 µg, 100 ng, 10 ng
Poly(I:C) HMW	TLR3	10 µg, 1 µg, 100 ng, 10 ng
Poly(I:C) LMW	TLR3	10 µg, 1 µg, 100 ng, 10 ng
Zymosan	TLR2/Dectin 1	10 µg, 1 µg, 100 ng, 10 ng
CLO75	TLR8	10 µg, 1 µg, 100 ng, 10 ng
MDP	NOD2	10 µg, 1 µg, 100 ng, 10 ng
ODN2216	TLR9	5 µg, 500 ng, 50 ng, 5 ng
*E. coli* O111:B4 LPS	TLR4	1 ng, 100 pg, 10 pg, 1 pg

## Data Availability

Materials used in this study are available from commercial vendors; the data used to produce figures in this manuscript maybe requested by contacting principal investigators of the study.

## Supplementary Materials

The following are available online, Table S1: Endotoxin and β-glucan Levels in Teriparatide Formulation, Figure S1: PBMC Viability in the Presence of Teriparatide, Figure S2: In vitro Leukocyte Proliferation in the Presence of Teriparatide and/or Innate Immune Response Modulating Impurities, Figure S3: In vitro Complement Activation Induced by Teriparatide, Figure S4: 16-plex Induction of Cytokines in PBMCs, Figure S5: Formulation Buffer is Responsible for the Cytokine Response to Teriparatide, Figure S6: Metacresol and Mannitol are Responsible for the Formulation Buffer Cytokine Response, Figure S7: Normalized 16-plex Cytokine Response to Innate Immune Response Modulating Impurities and Selection of One Signature Cytokine, Figure S8: Normalized 16-plex Cytokine Response in the Combined Presence of Innate Immune Response Modulating Impurities and Teriparatide, Figure S9: Innate Immune Response Modulating Impurity-Induced Cytokine Response Patterns via Euclidian Distance and Ward’s Clustering, Figure S10: Cytokine Analysis via Pearson’s Correlation, Figure S11: 7-plex Induction of Cytokines in PBMCs, Figure S12: Normalized 7-plex Cytokine Response to Innate Immune Response Modulating Impurities, Figure S13: Formulation Buffer Affects Cytokines Induced by Innate Immune Response Modulating Impurities, Figure S14: Reproducibility of Cytokine Response to Teriparatide in PBMC Cultures, Figure S15: Normalized Cytokine Responses to Innate Immune Response Modulating Impurities are Affected by PBMC and Blood Handling Conditions.
