# Supplementary material for: An In Vitro Assessment of Immunostimulatory Responses to Ten Model Innate Immune Response Modulating Impurities (IIRMIs) and Peptide Drug Product, Teriparatide
Source: Molecules. 2021 Dec 9;26(24):7461. doi: 10.3390/molecules26247461 (PMC8707785; doi:10.3390/molecules26247461)
Supplement: Supplementary file 1 [file molecules-26-07461-s001.zip › molecules-1469474-supplementary.pdf]

molecules MDPI

Supplementary Materials

An *In Vitro* Assessment of Immunostimulatory Responses to Ten Model Innate Immune Response Modulating Impurities (IIRMIs) and Peptide Drug Product, Teriparatide

Claire K. Holley [1,†], Edward Cedrone [1,†], Duncan Donohue [2,†], Barry W. Neun [1], Daniela Verthelyi [3], Eric S. Pang [4,*] and Marina A. Dobrovolskaia [1,*]

[1] Nanotechnology Characterization Laboratory, Cancer Research Technology Program, Frederick National Laboratory for Cancer Research Sponsored by the National Cancer Institute, Frederick, MD 21702, USA; claire.holley@nih.gov (C.K.H.); edward.cedrone@nih.gov (E.C.); neunb@mail.nih.gov (B.W.N.)
[2] Statistics Department, Data Management Services Inc., Frederick National Laboratory for Cancer Research Sponsored by the National Cancer Institute, Frederick, MD 21702, USA; duncan.donohue@nih.gov
[3] Laboratory of Innate Immunity, Division of Biotechnology Review and Research-III, Office of Biotechnology Products, Center for Drug Evaluation and Research, U.S. Food and Drug Administration, Silver Spring, MD 20993, USA; daniela.verthelyi@fda.hhs.gov
[4] Division of Therapeutic Performance, Office of Research and Standards, Office of Generic Drugs, Center for Drug Evaluation and Research, U.S. Food and Drug Administration, Silver Spring, MD 20993, USA
* Correspondence: eric.pang@fda.hhs.gov (E.S.P.); marina@mail.nih.gov (M.A.D.)
† These authors contributed equally.

Table S1. Endotoxin and β-glucan Levels in Teriparatide Formulation. The pharmaceutical formulation of teriparatide (TP) and its formulation buffer (FB) were tested for the presence of endotoxin and β-glucans.

Sample	Working Concentration per mL	Endotoxin (EU/mL) (% Spike Recovery)	β-Glucans (pg/mL) (% Spike Recovery)
TP	250 µg	<0.05 (89)	<25 (82)
FB	250 µg	<0.05 (136)	<25 (98)

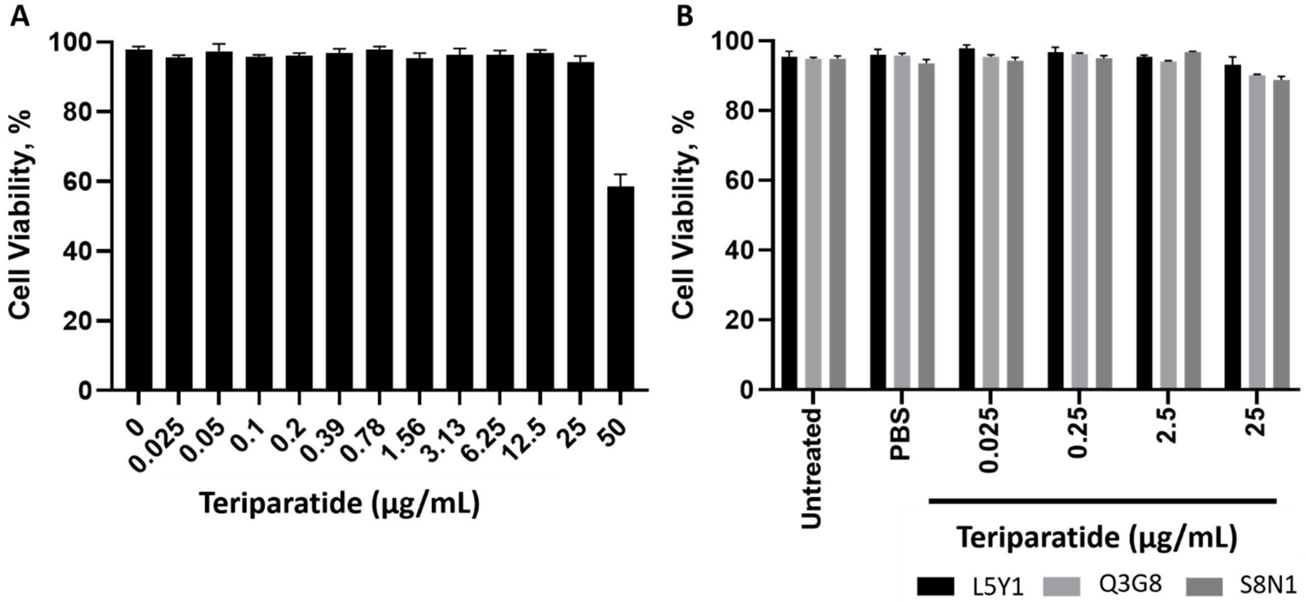

Figure S1. PBMC Viability in the Presence of Teriparatide. (A) PBMCs purified from the blood of one healthy human donor were treated with 0.025 - 50 µg/mL TP for 24 hours. Cell viability was then assessed using AO/PI. (B) The results

were verified in three healthy human donors treated with 0.025, 0.25, 2.5, and 25 µg/mL TP. Each bar shows the mean result and standard deviation of four independent samples (N=4).

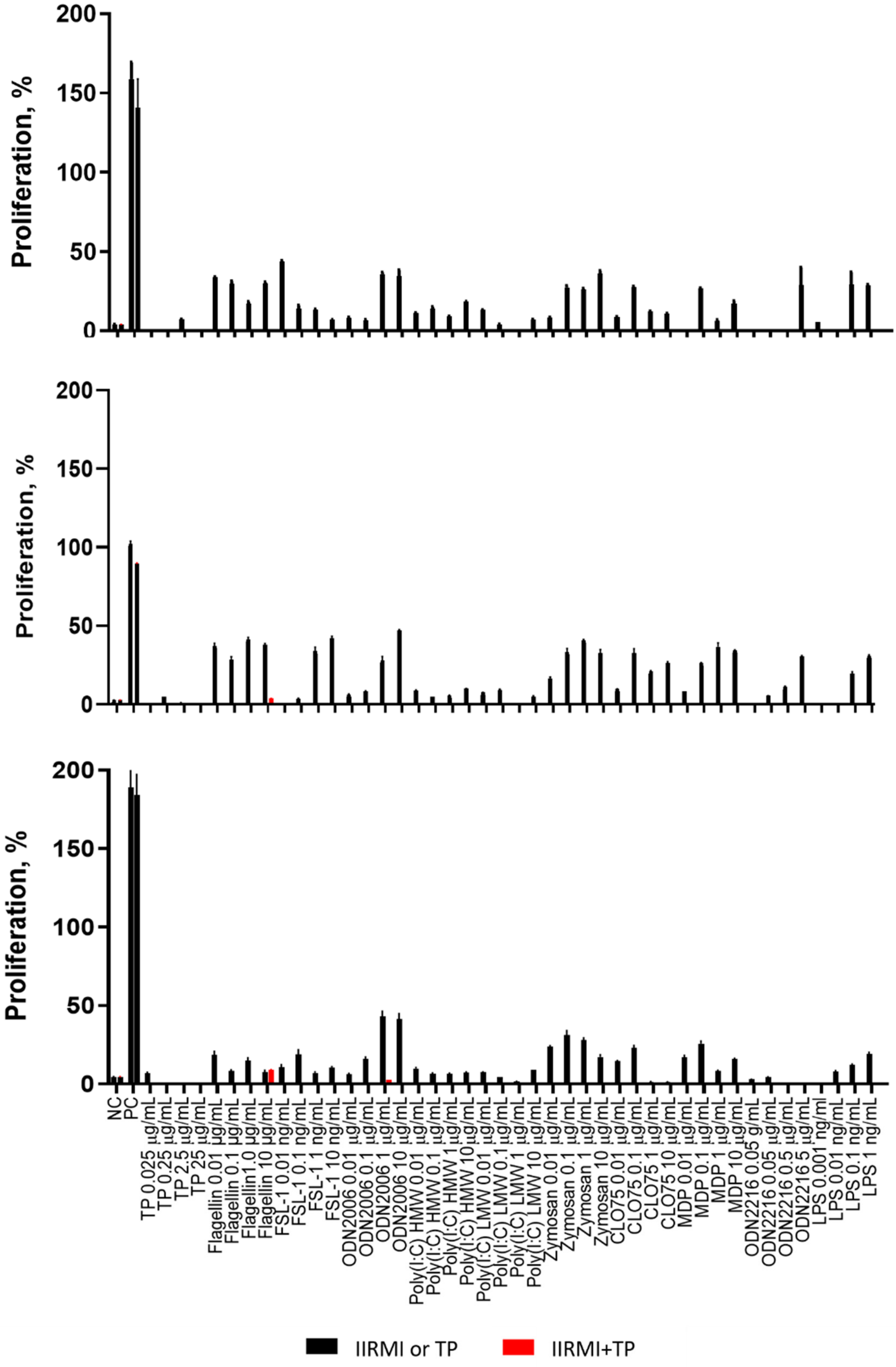

Figure S2. In vitro Leukocyte Proliferation in the Presence of Teriparatide and/or Innate Immune Response Modulating Impurities. PBMCs from three healthy human donors were cultured for 72 hours in the presence of PBS as negative control (NC), Phytohemagglutinin (PHA-M) as positive control (PC), TP, IIRMIs, or IIRMIs+25 µg/mL TP. Following incubation,

the proliferation of leukocytes was estimated using the MTT reagent. Percent proliferation was calculated by comparing the mean O.D. of test samples to that of the baseline. Each bar shows the mean result and standard deviation of three independent samples (N=3).

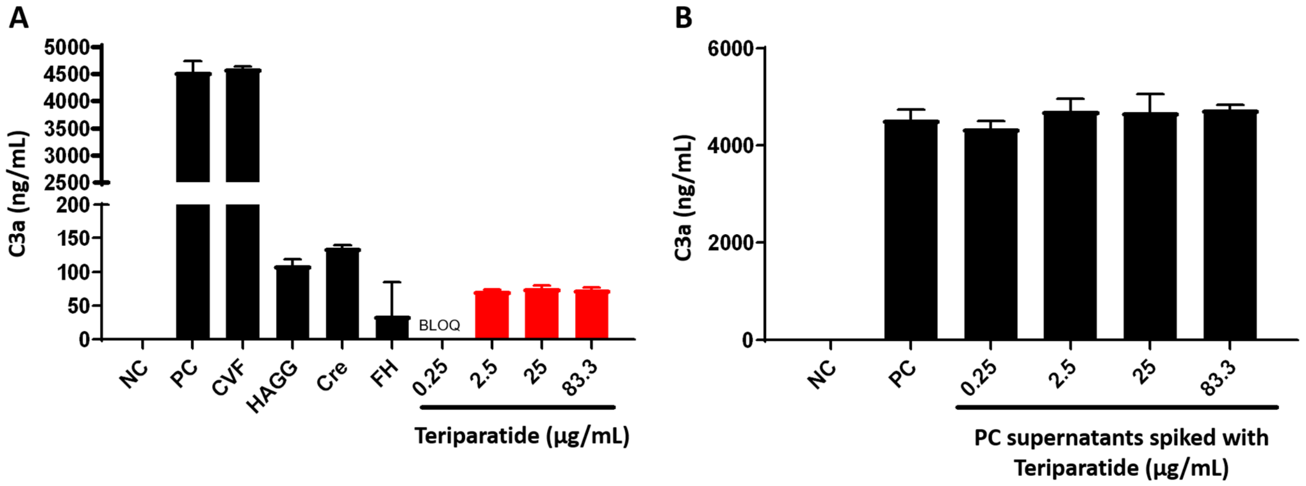

Figure S3. In vitro Complement Activation Induced by Teriparatide. (A) Plasma obtained from healthy human donors was treated with 0.25, 2.5, 25, and 83.3 μg/mL TP for 30 min. PBS was used as a negative control (NC) and a combination of Cobra Venom Factor (CVF) and Heat Aggregated Gamma Globulins (HAGG) was used as positive control (PC). For comparison, individual samples of CVF, HAGG, Cremophor-EL (Cre), and Feraheme (FH) were examined as additional controls. (B) Further, to determine potential TP interference with the assay and rule out false negative results, PC supernatant was spiked with 0.25, 2.5, 25, and 83.3 μg/mL TP. Complement activation was assessed by ELISA assay detecting C3a split product. Shown is the mean response (N=3) ± SD. BLOQ = below the limit of quantification.

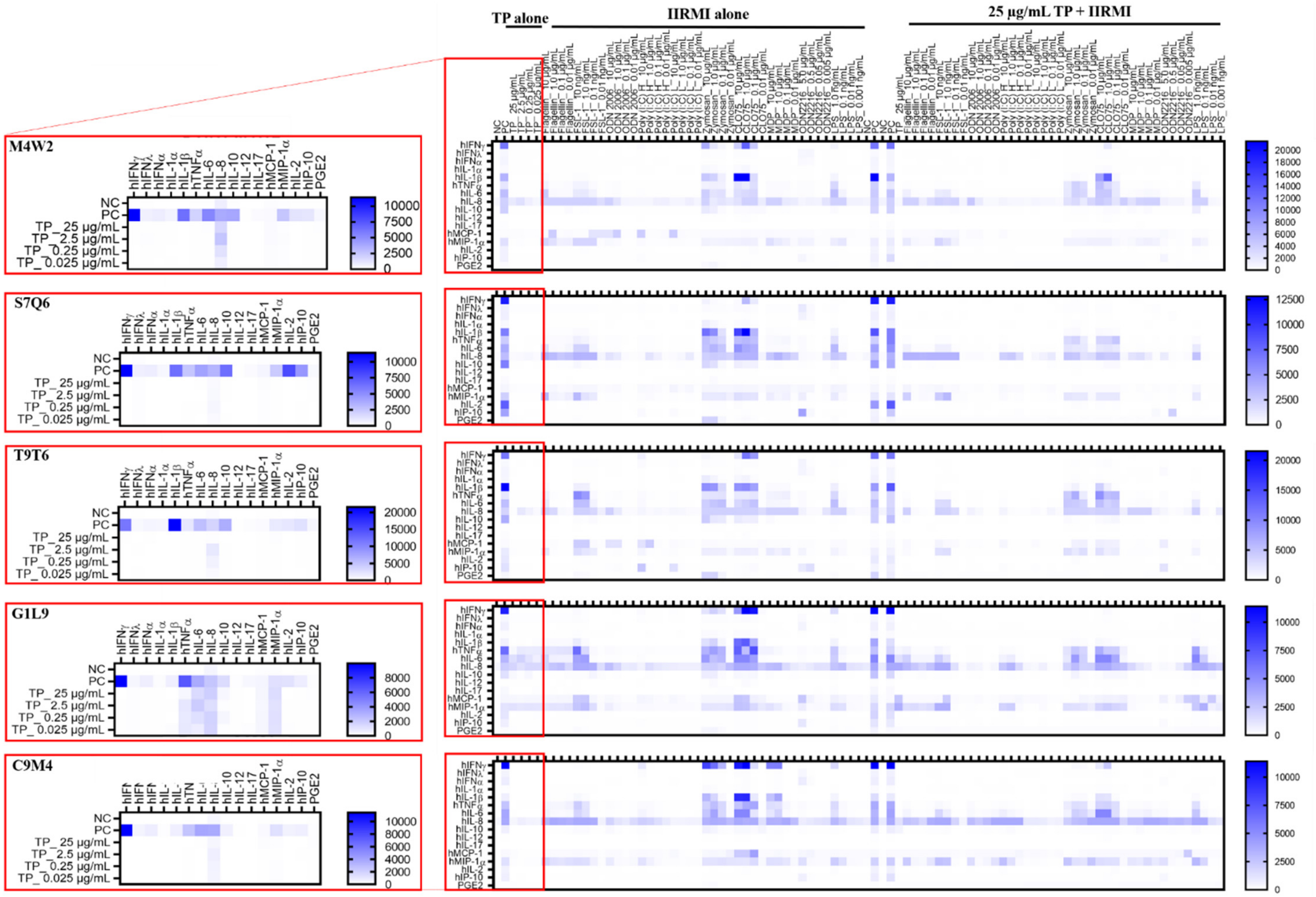
TP alone
IIRMI alone
25 μg/mL TP + IIRMI
M4W2
S7Q6
T9T6
G1L9
C9M4
NC
PC
TP_25 μg/mL
TP_2.5 μg/mL
TP_0.25 μg/mL
TP_0.025 μg/mL
hIFNγ
hIFNλ
hIFNα
hIL-1α
hIL-1β
hTNFα
hIL-6
hIL-8
hIL-10
hIL-12
hIL-17
hMCP-1
hMIP-1α
hIL-2
hIP-10
PGE2

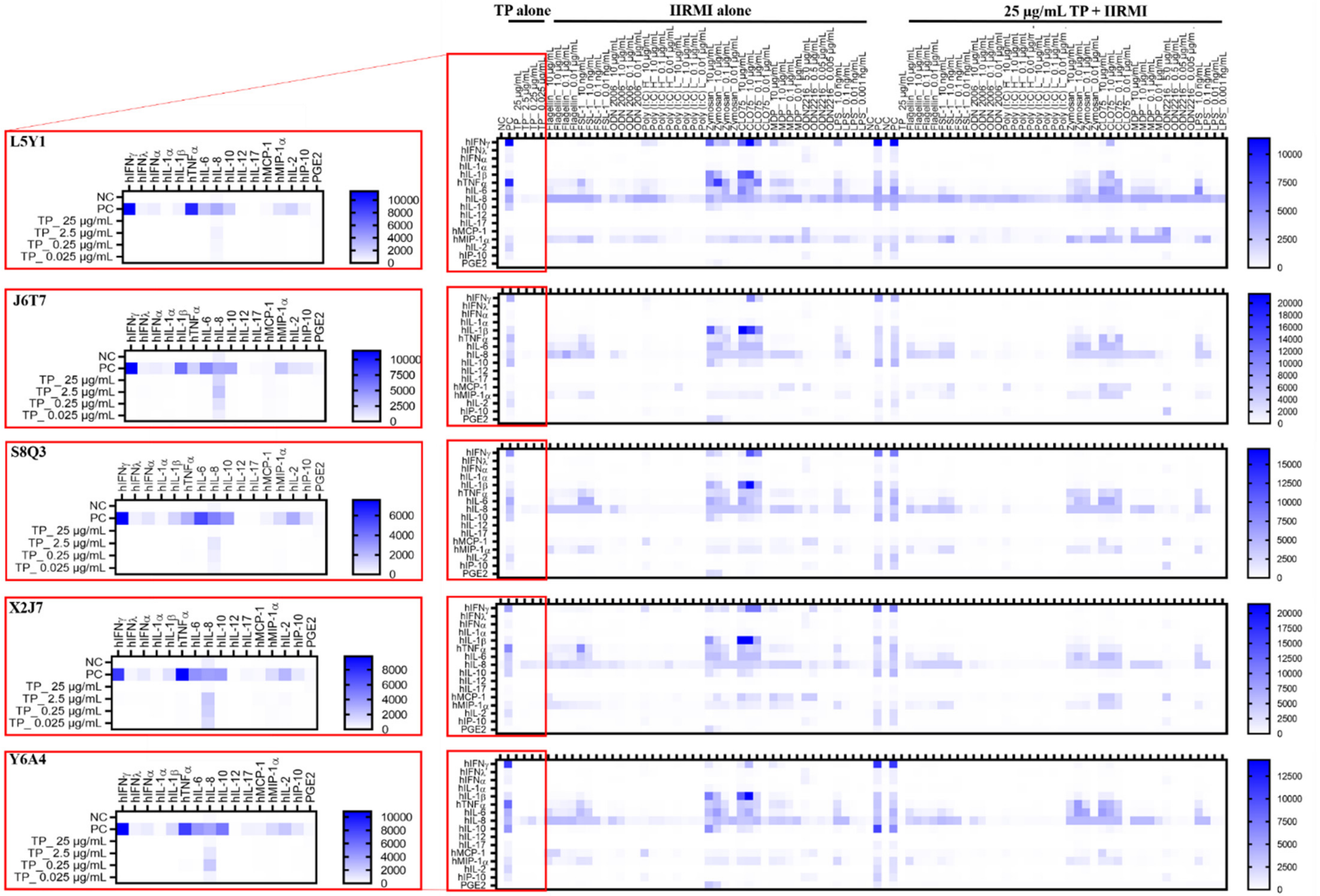

Figure S4. 16-plex Induction of Cytokines in PBMCs. PBMCs collected from 10 healthy human donors were treated with 0.025, 0.25, 2.5, and 25 µg/mL TP (red box), IIRMIs, or a combination of 25 µg/mL TP+IIMIs for 24 hours. Supernatants were analyzed for the presence of cytokines by multiplex ELISA. Shown is the mean response (N=2).

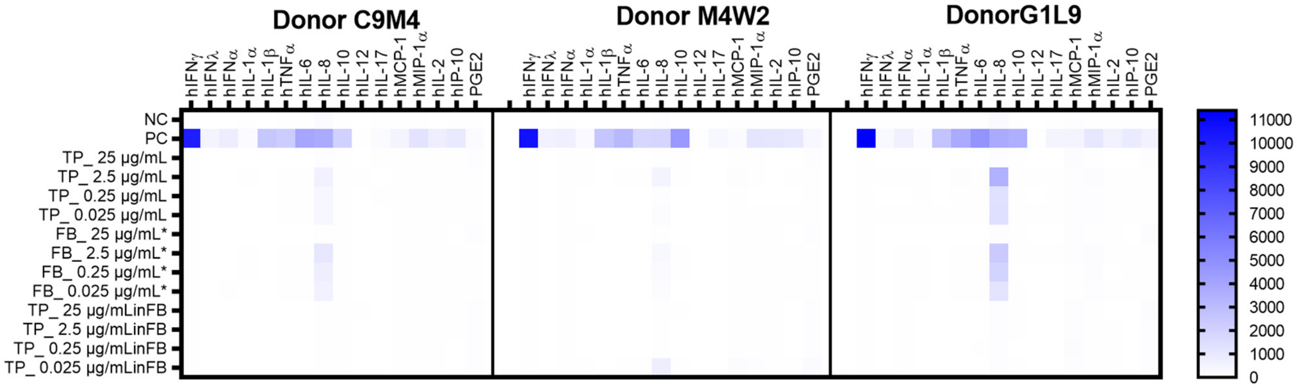

Figure S5. Formulation Buffer is Responsible for the Cytokine Response to Teriparatide. To determine if the observed cytokine response is induced by TP or FB, PBMCs from three healthy human donors were treated with 0.025, 0.25, 2.5, and 25 µg/mL TP diluted in PBS or FB, compared to equivalent concentrations of FB alone for 24 hours. The supernatant was collected and analyzed for cytokine levels via multiplex ELISA.

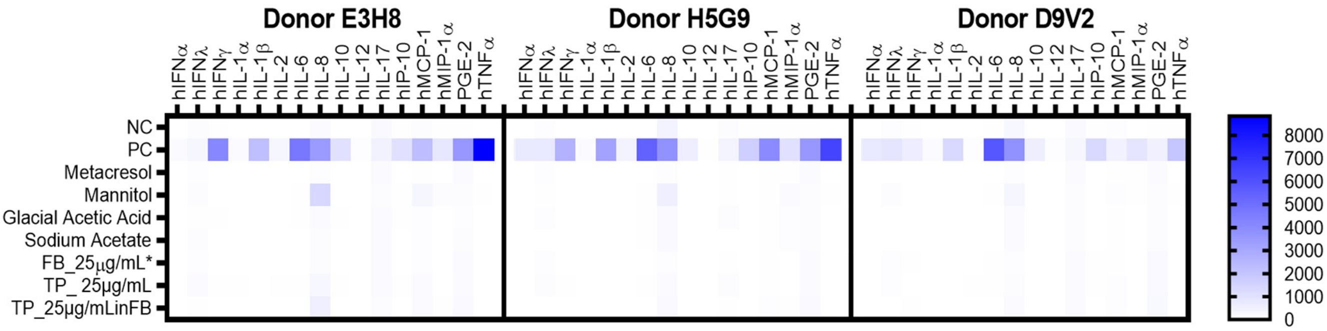

Figure S6. Metacresol and Mannitol are Responsible for the Formulation Buffer Cytokine Response. To determine which component of the formulation buffer is responsible for the observed cytokine response, PBMCs from three healthy human donors were treated with 25 µg/mL TP diluted in PBS or FB, compared to equivalent concentrations of FB or the individual buffer components (metacresol, mannitol, glacial acetic acid, and sodium acetate) alone for 24 hours. The supernatant was collected and analyzed for cytokine levels via multiplex ELISA. Each data point represents a mean response (N=2).

A. Flagellin

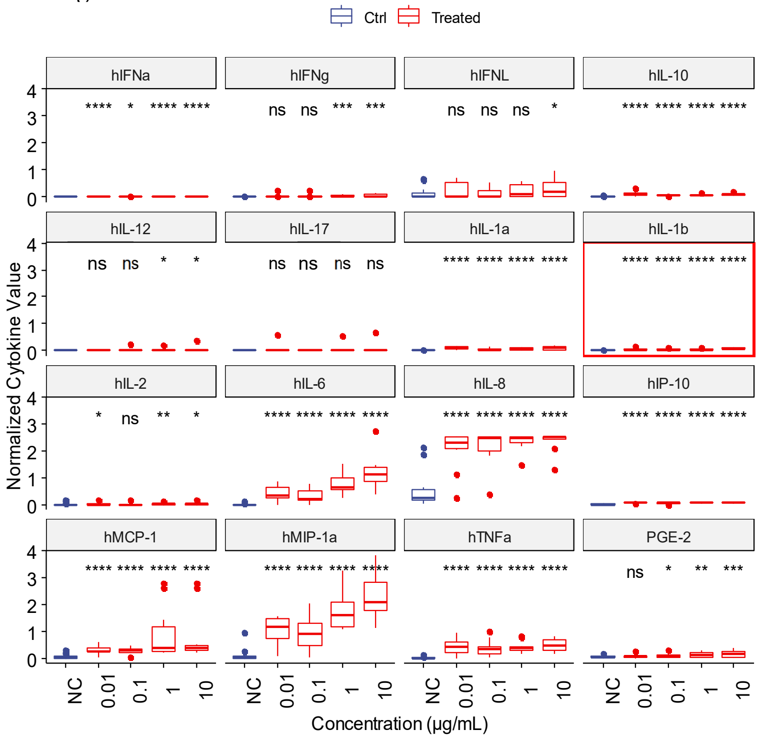

B. FSL-1

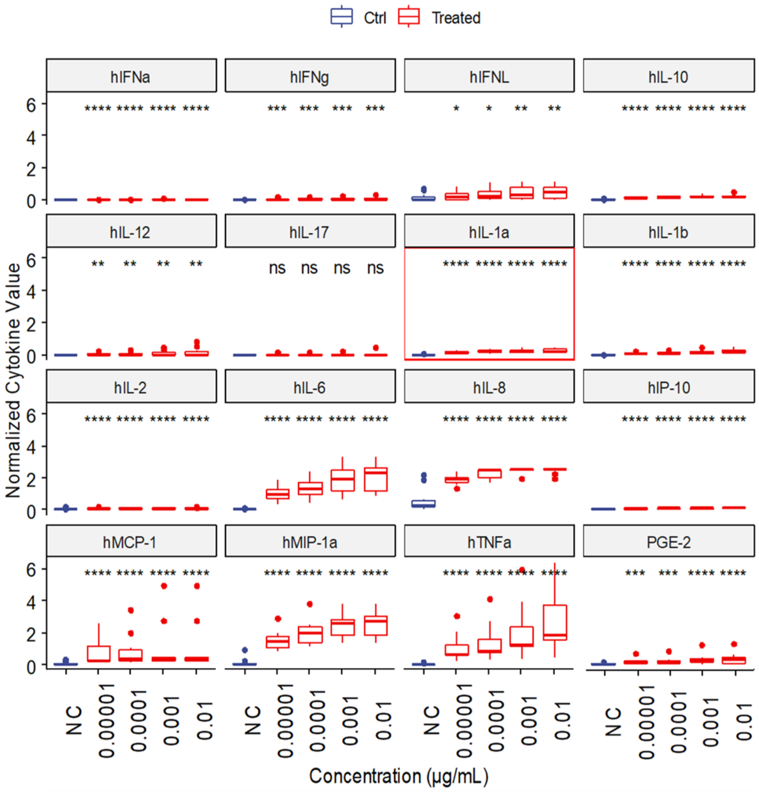

C. ODN2006

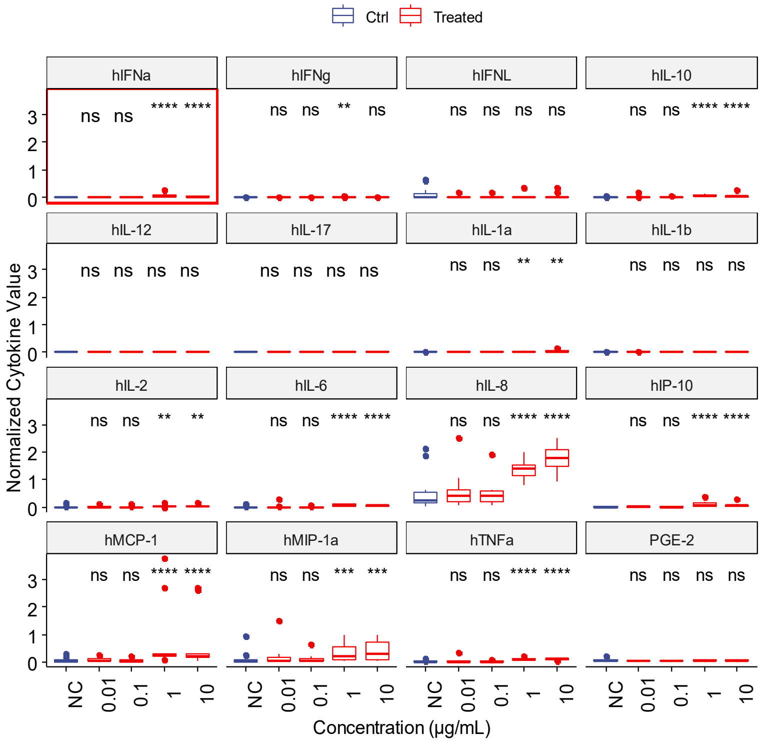

D. Poly(I:C) HMW

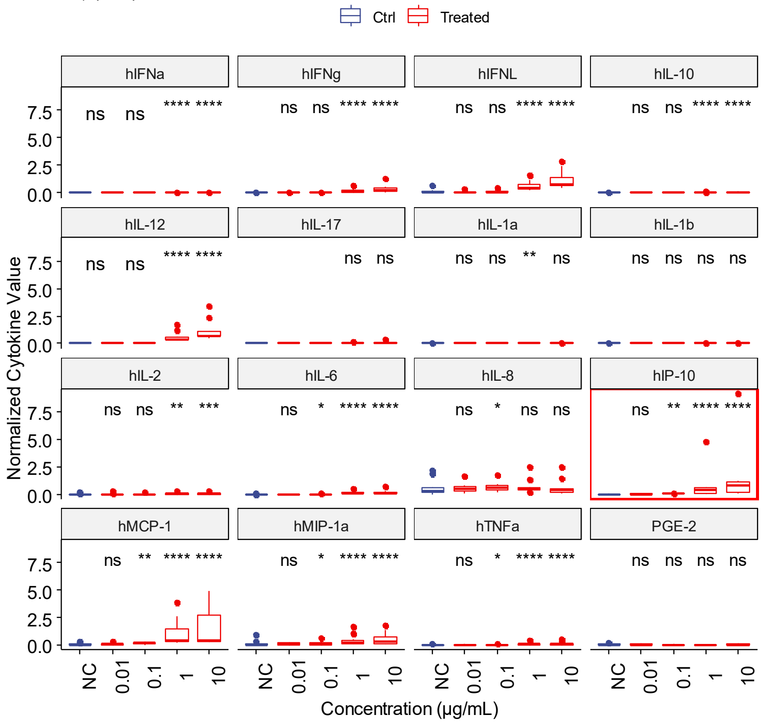

E. Poly(I:C) LMW

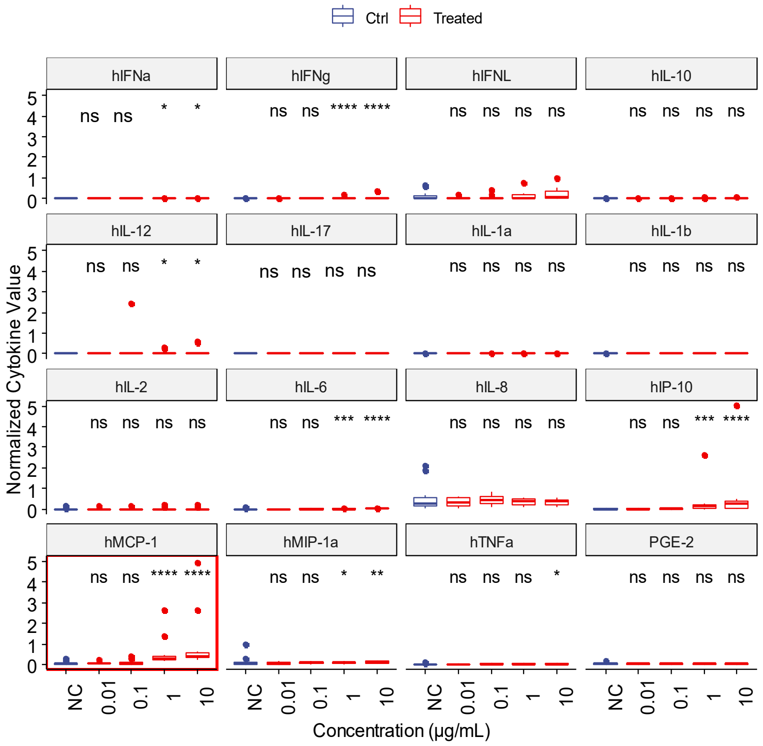

F. Zymosan

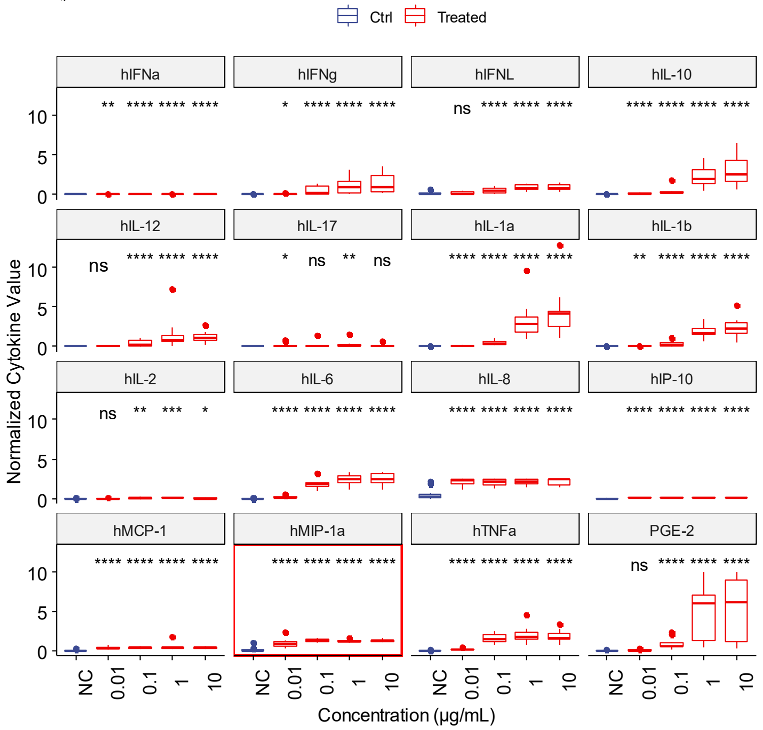

G. CLO75

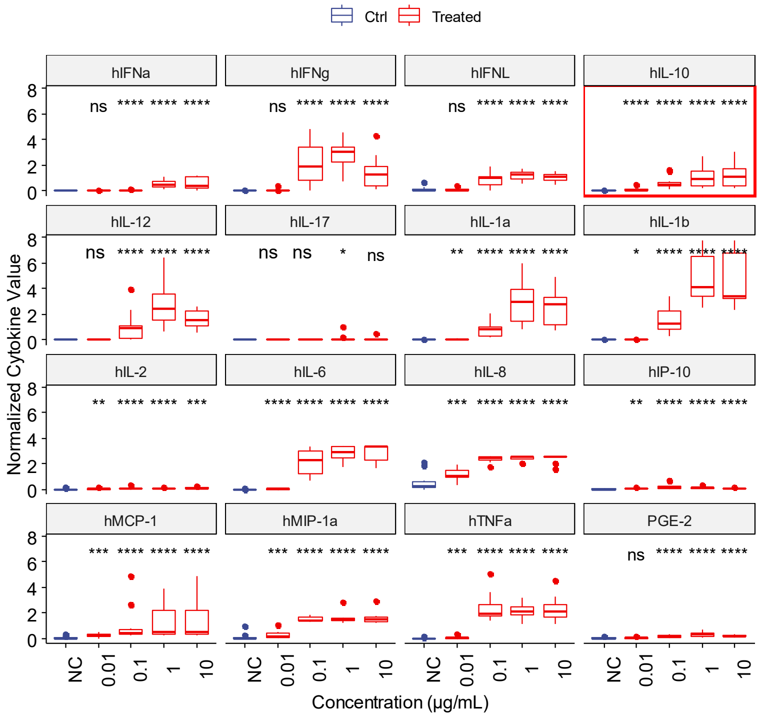
Ctrl
Treated
hIFNa
hIFNg
hIFNL
hIL-10
hIL-12
hIL-17
hIL-1a
hIL-1b
hIL-2
hIL-6
hIL-8
hIP-10
hMCP-1
hMIP-1a
hTNFa
PGE-2
Normalized Cytokine Value
NC
0.01
0.1
1
10
Concentration (μg/mL)

H. MDP

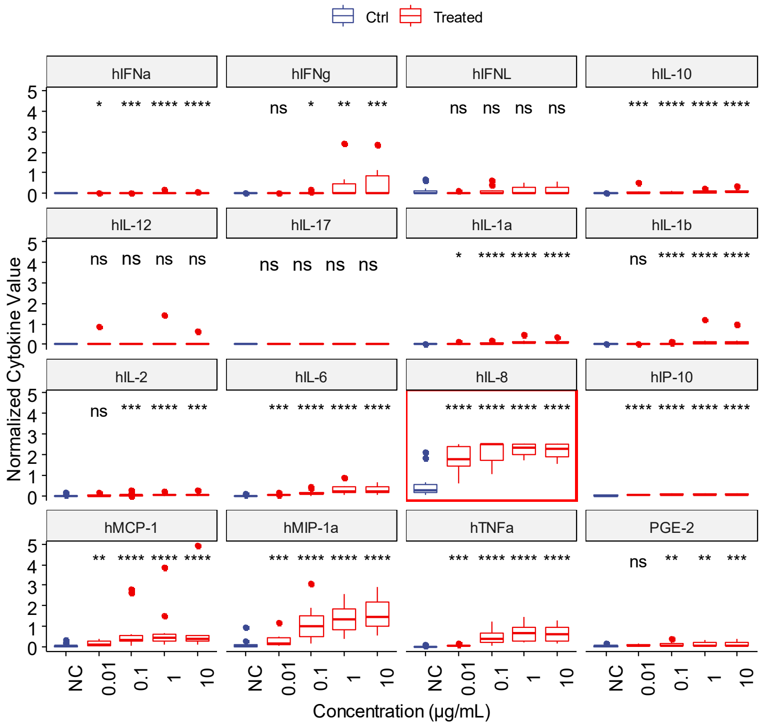
Ctrl
Treated
hIFNa
hIFNg
hIFNL
hIL-10
hIL-12
hIL-17
hIL-1a
hIL-1b
hIL-2
hIL-6
hIL-8
hIP-10
hMCP-1
hMIP-1a
hTNFa
PGE-2
Normalized Cytokine Value
NC
0.01
0.1
1
10
Concentration (μg/mL)

I. ODN2216

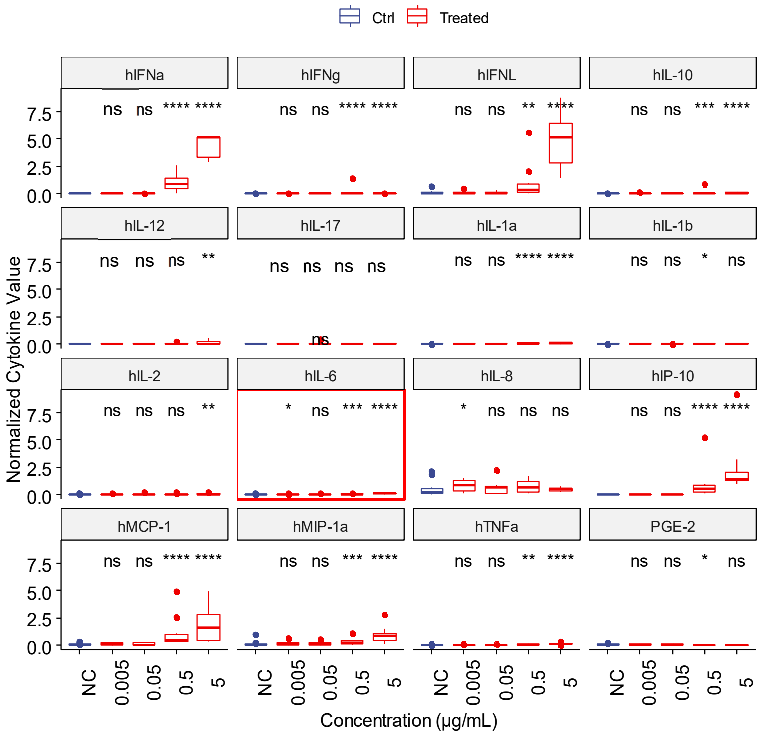

J. O111:B4 LPS

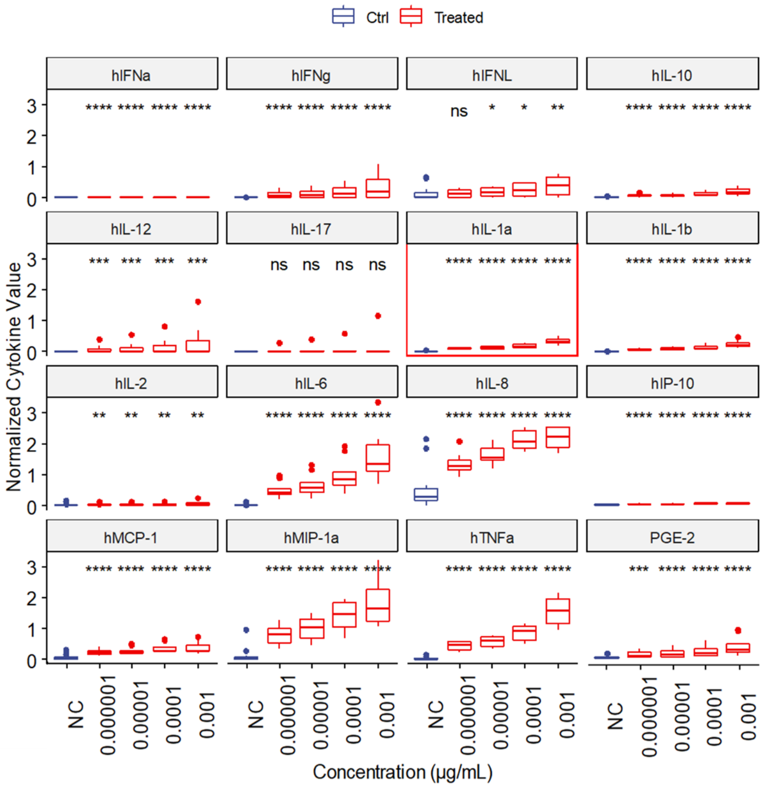

Figure S7. Normalized 16-plex Cytokine Response to Innate Immune Response Modulating Impurities and Selection of One Signature Cytokine. PBMCs from 10 healthy human donors were treated with IIRMIs for 24 hours. Supernatants were analyzed for the presence of cytokines by multiplex ELISA. Shown are the mean cytokine responses to individual IIRMI concentrations (red) of (A) flagellin, (B) FSL-1, (C) ODN2006, (D) poly(I:C) HMW, (E) poly(I:C) LMW, (F) zymosan, (G) CLO75, (H) MDP, (I) ODN2216, and (J) O111:B4 LPS as well as negative control (NC, blue). The signature cytokine, highlighted using a red box, is the one for which the IIRMI concentration, when compared to the negative control (NC), results in a $p<0.05$. The data for which statistical significance was not observed are marked with ns. Statistical significance is shown with an asterisk as follows: *$p<0.05$; **$p<0.01$; ***$p<0.001$; and ****$p<0.0001$.

A. Flagellin

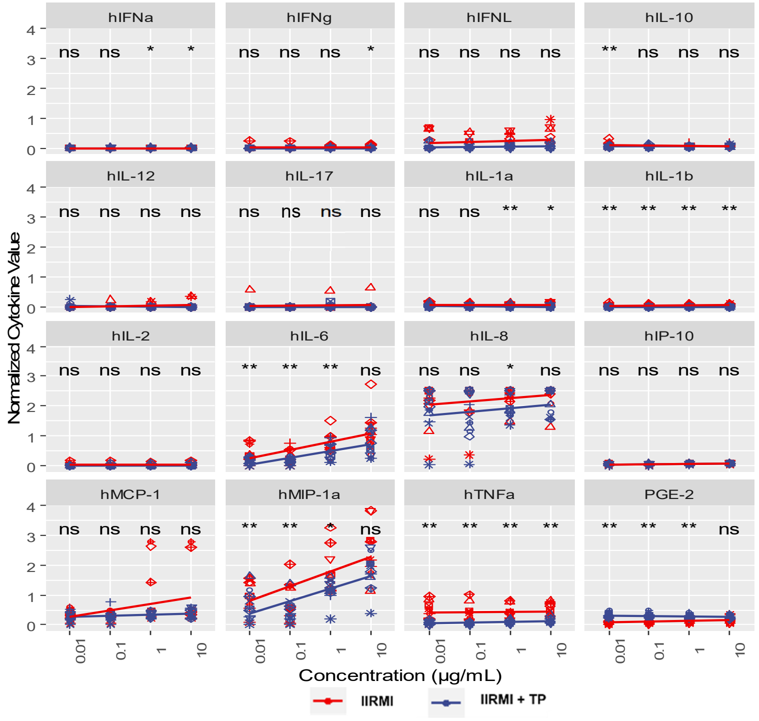

B. FSL-1

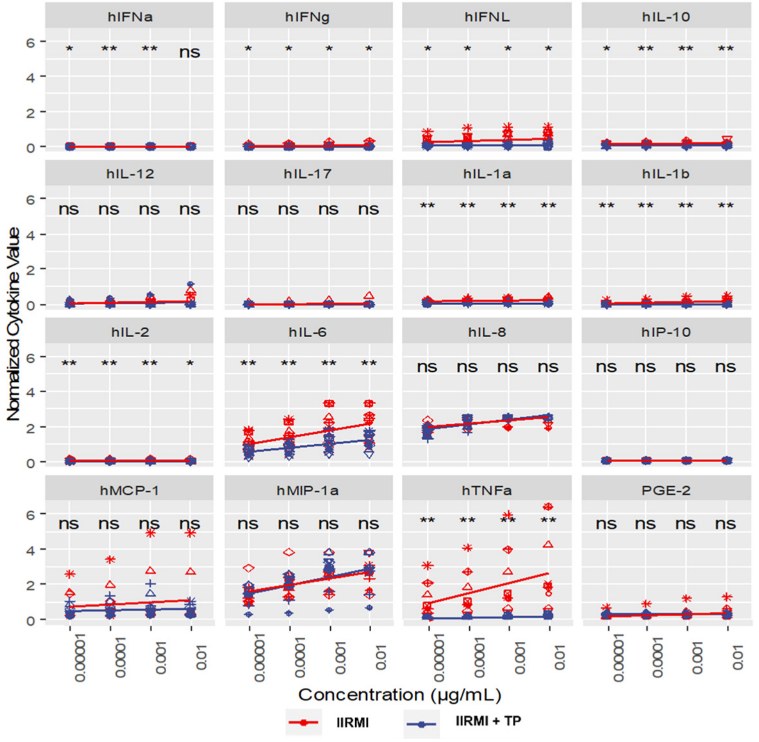

C. ODN2006

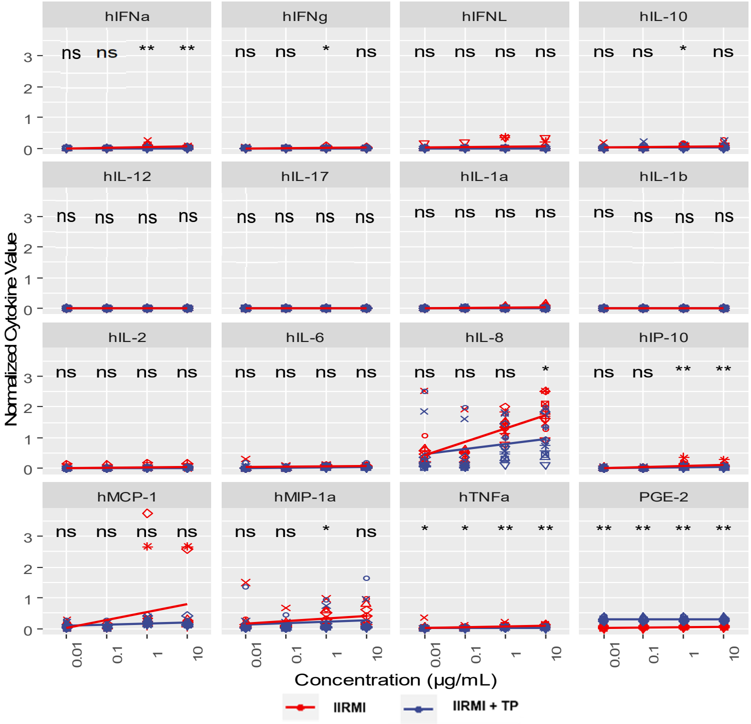

D. Poly(I:C) HMW

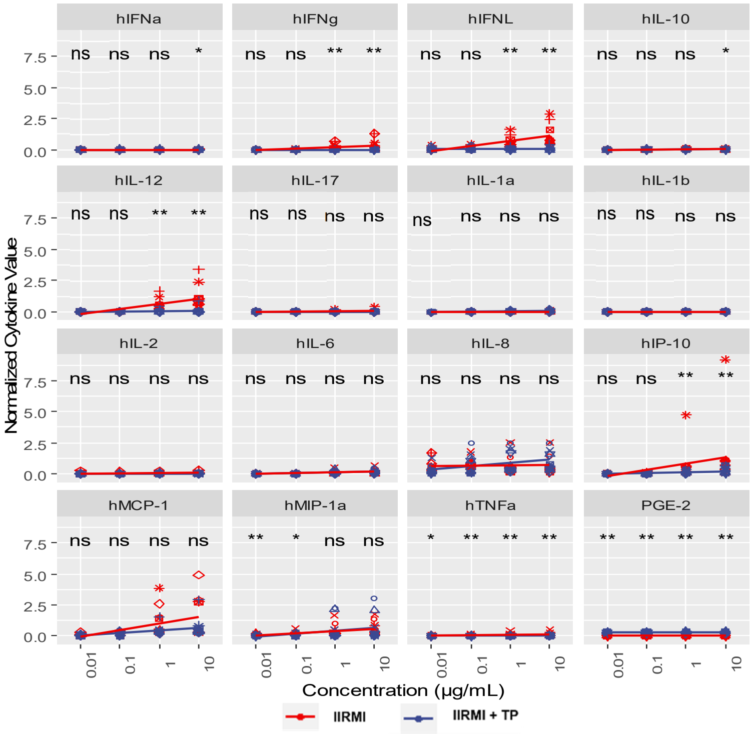

E. Poly(I:C) LMW

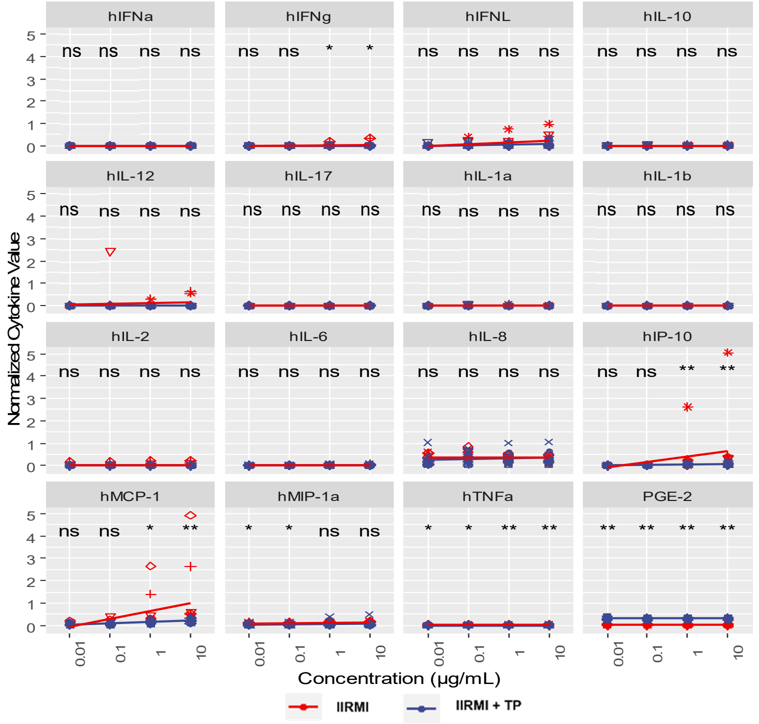

F. Zymosan

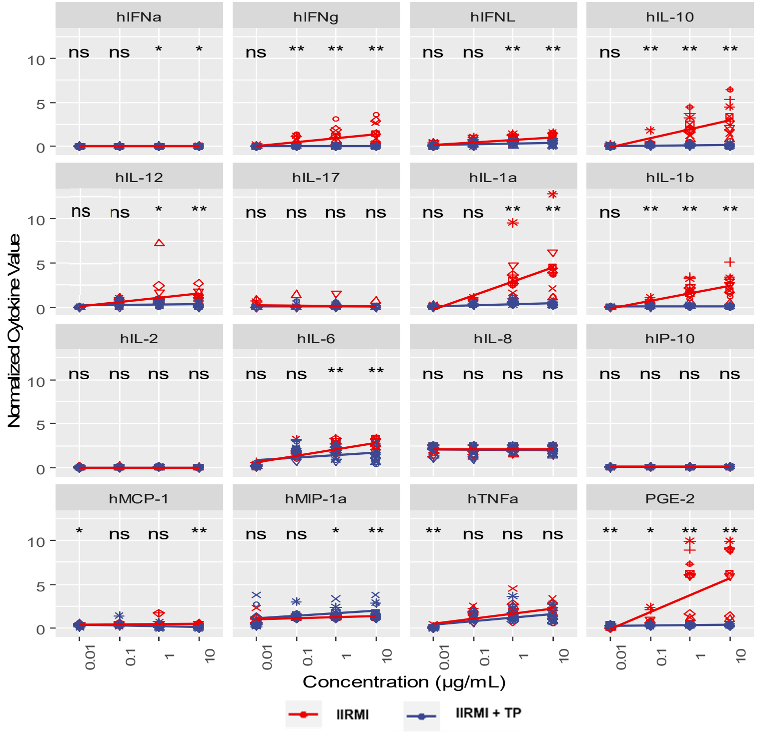

G. CLO75

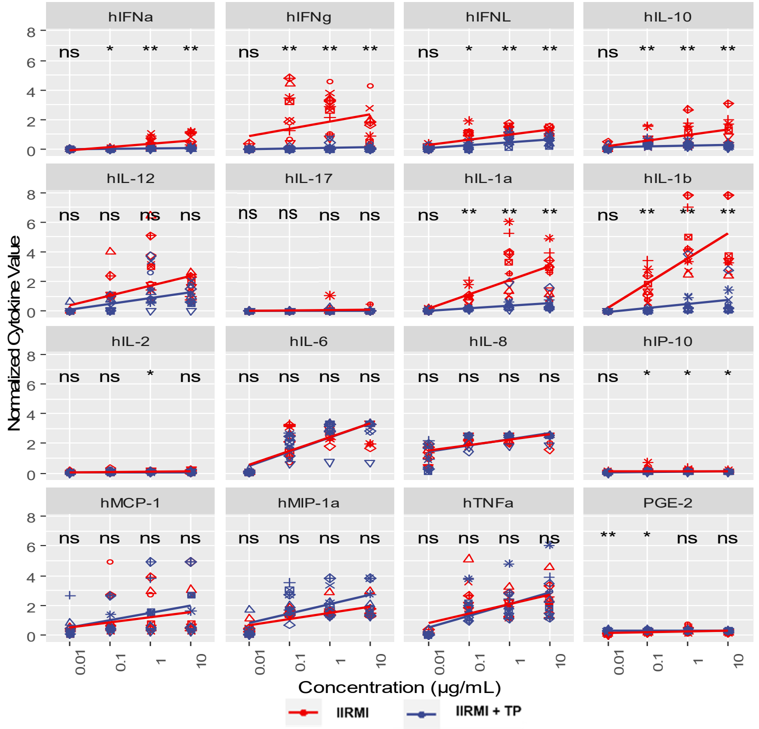
hIFNa
hIFNg
hIFNL
hIL-10
hIL-12
hIL-17
hIL-1a
hIL-1b
hIL-2
hIL-6
hIL-8
hIP-10
hMCP-1
hMIP-1a
hTNFa
PGE-2
Normalized Cytokine Value
Concentration (µg/mL)
IIRMI
IIRMI + TP

H. MDP

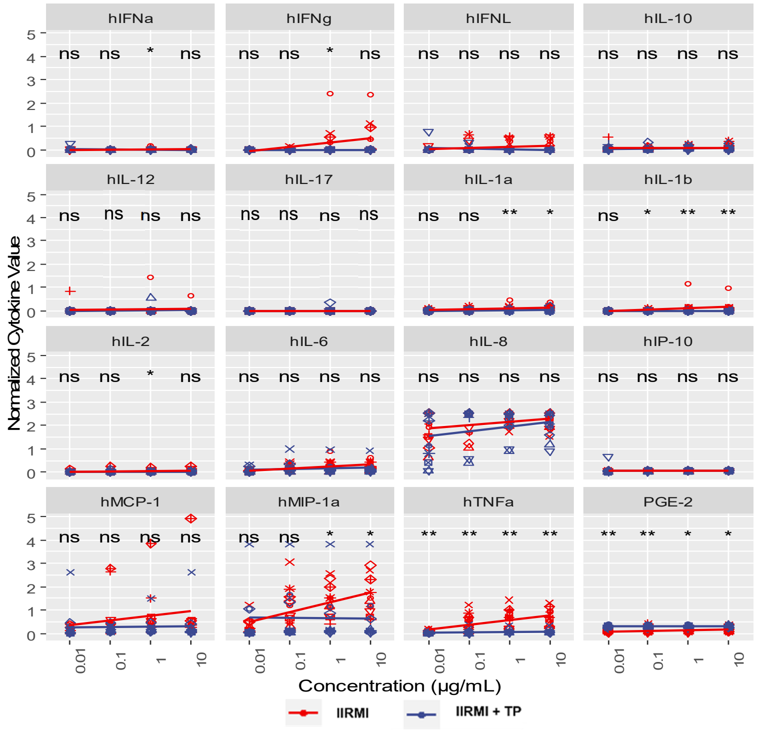
hIFNa
hIFNg
hIFNL
hIL-10
hIL-12
hIL-17
hIL-1a
hIL-1b
hIL-2
hIL-6
hIL-8
hIP-10
hMCP-1
hMIP-1a
hTNFa
PGE-2
Normalized Cytokine Value
Concentration (µg/mL)
IIRMI
IIRMI + TP

I. ODN2216

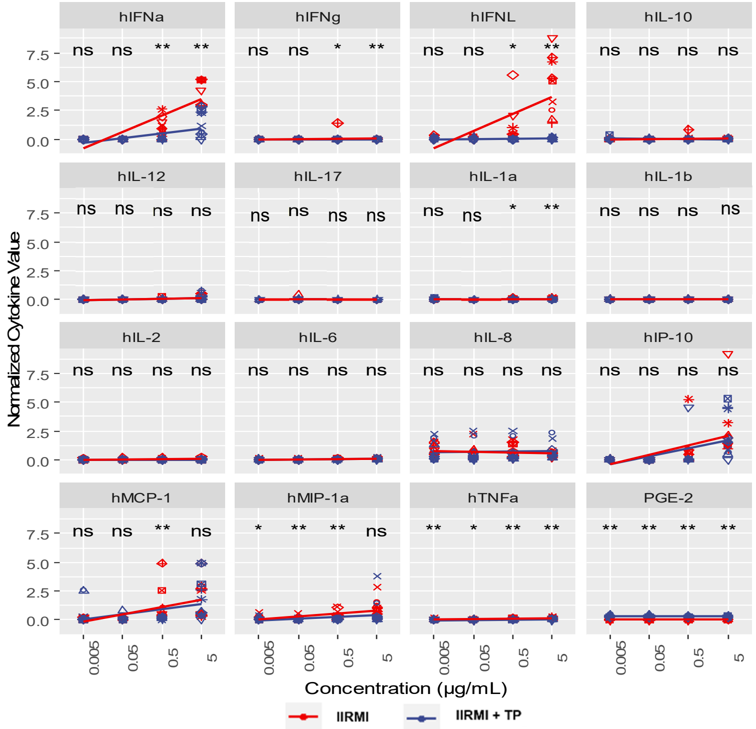

J. O111:B4 LPS

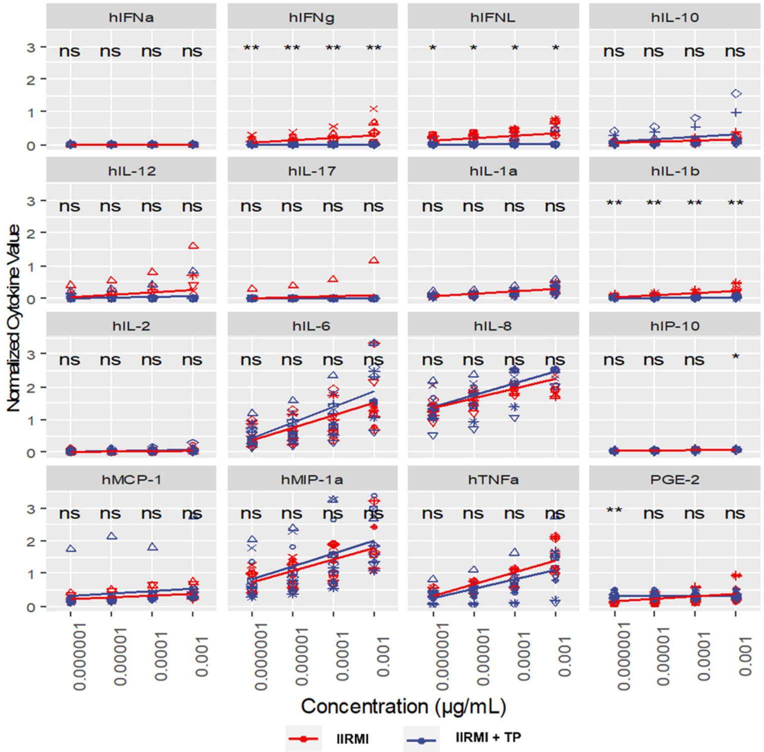

Figure S8. Normalized 16-plex Cytokine Response in the Combined Presence of Innate Immune Response Modulating Impurities and Teriparatide. PBMCs from 10 healthy human donors were treated with IIRMI alone (red line) or in combination with 25 μg/mL TP (blue line) for 24 hours. Supernatants were analyzed for the presence of cytokines, of which the mean responses from individual donors at various IIRMI concentrations are highlighted by different geometric shapes. Shown are the normalized cytokine responses, the result of individual cytokine response in pg/mL divided by the standard deviation of all responses for that cytokine across all donors and concentrations, to individual IIRMI concentrations of (A) flagellin, (B) FSL-1, (C) ODN2006, (D) poly(I:C) HMW, (E) poly(I:C) LMW, (F) zymosan, (G) CLO75, (H) MDP, (I) ODN2216, and (J) O111:B4 LPS. The data for which statistical significance was not observed are marked with ns. Statistical significance is shown with an asterisk as follows: *$p<0.05$; **$p<0.01$; ***$p<0.001$; and ****$p<0.0001$.

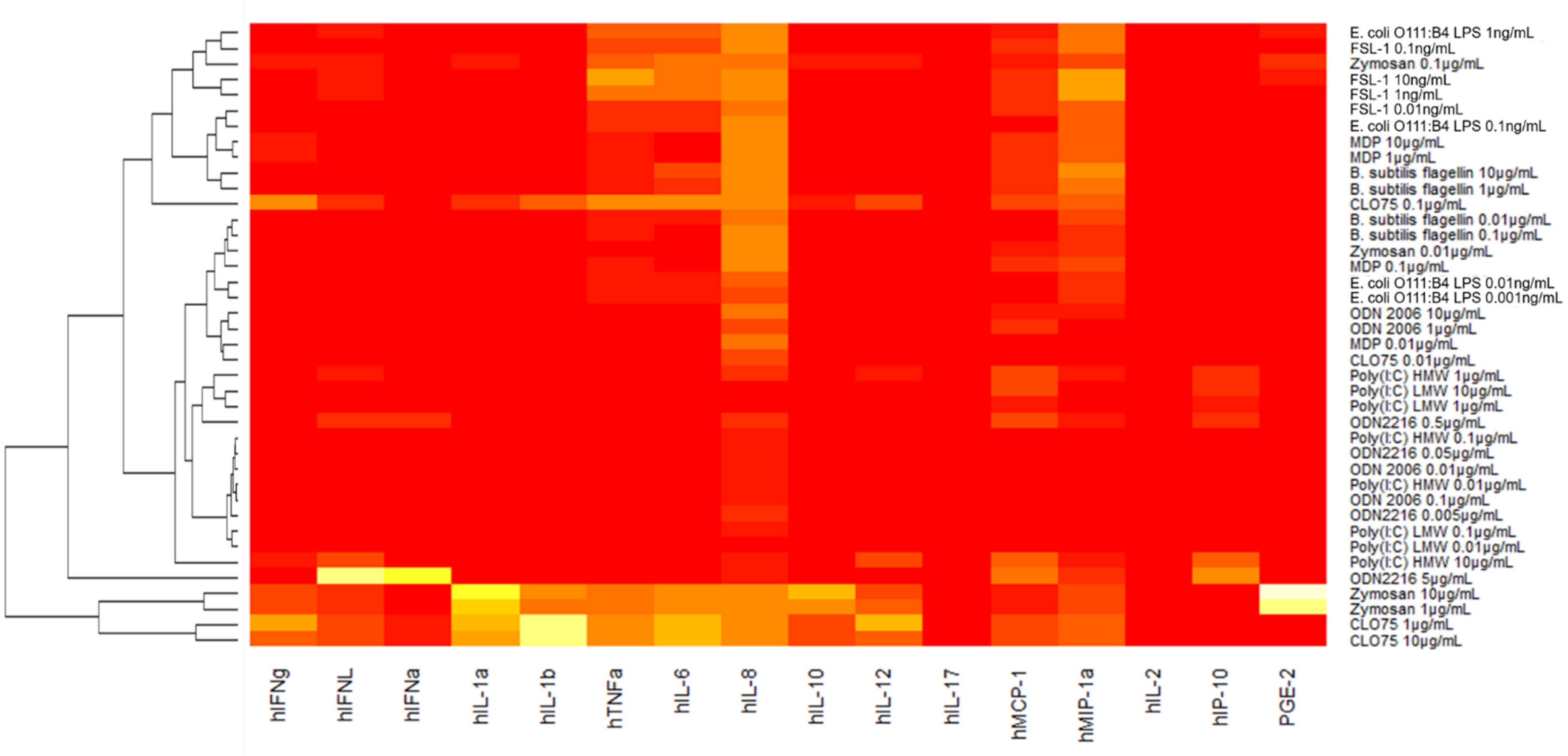

Figure S9. Innate Immune Response Modulating Impurity-Induced Cytokine Response Patterns via Euclidian Distance and Ward's Clustering. PBMCs from 10 healthy human donors were treated with various concentrations of IIRMI for 24 hours. Supernatants were analyzed for the presence of cytokines by multiplex ELISA. Shown is the mean response of normalized values averaged across all donors, clustered based on IIRMI treatment. Dendrograms were created using complete linkage clustering on the Euclidian distance matrices. Similar results for cytokine clustering available in Figure 4.

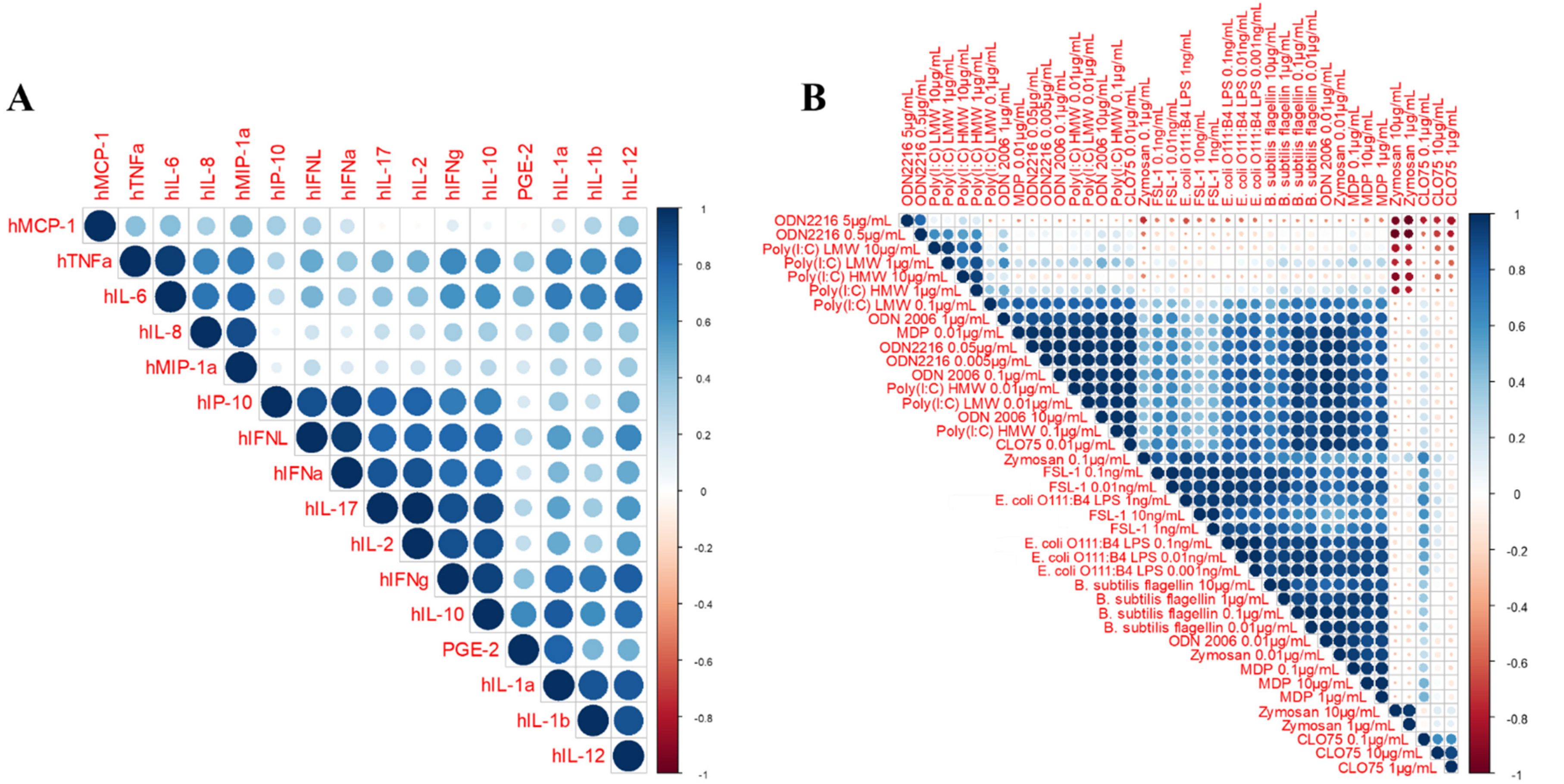

Figure S10. Cytokine Analysis via Pearson's Correlation. PBMC from 10 healthy human donor volunteers were treated with various concentrations of IIRMIs for 24 hours. Supernatants were analyzed for the presence of cytokines by multiplex ELISA. Shown is the mean response of normalized values averaged across all donors and replicates. (A) Pearson's correlation matrix of cytokine responses across all IIRMI. Cytokines are ordered based on their complete linkage hierarchical clustering of their correlation values. (B) Pearson's correlation matrix of all IIRMI/doses based on cytokine response values. IIRMI treatments/dose are ordered based on complete linkage hierarchical clustering of cytokine response value correlations.

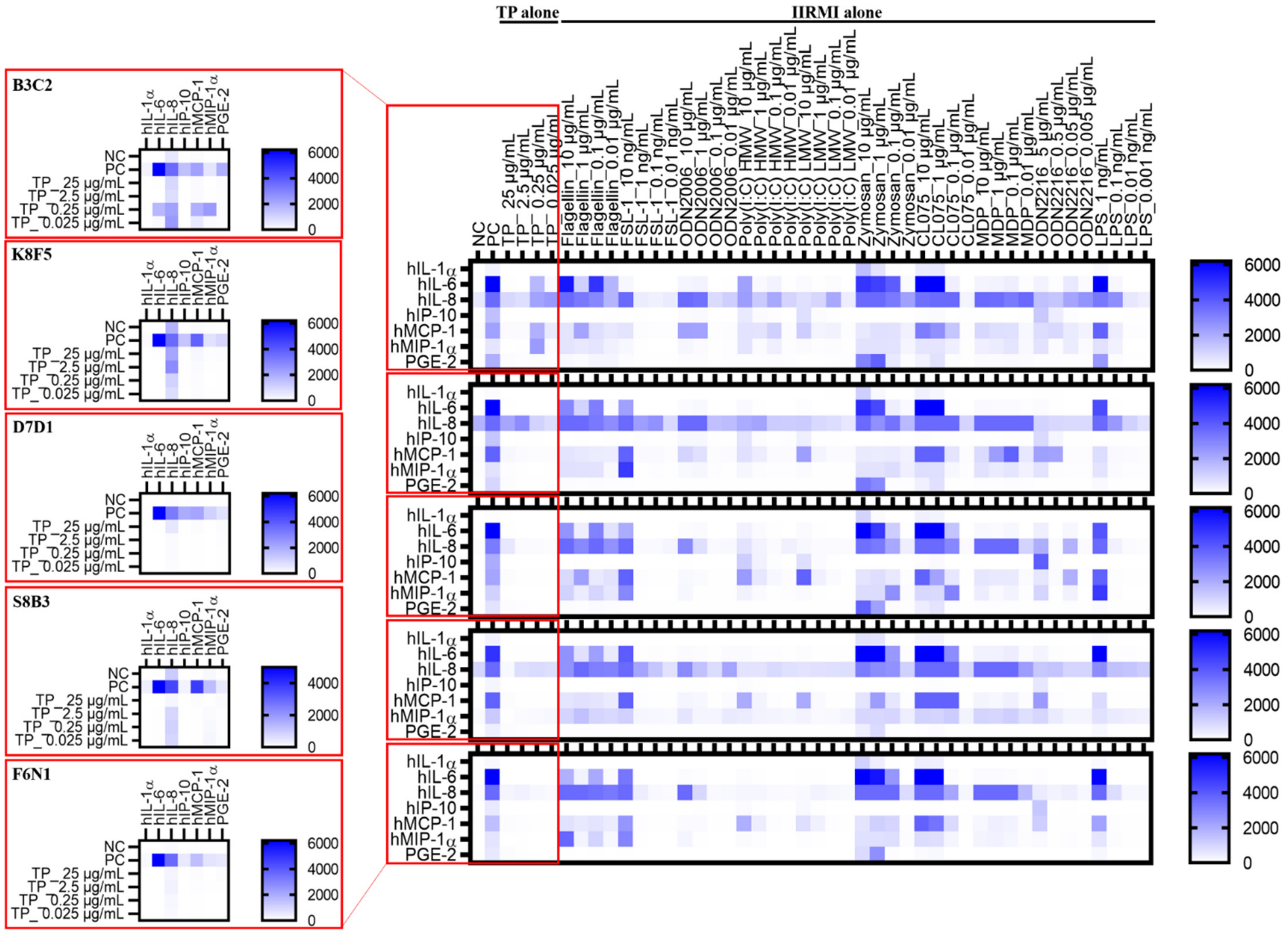

Figure S11. 7-plex Induction of Cytokines in PBMCs. PBMCs collected from 10 healthy human donors were treated with 0.025, 0.25, 2.5, and 25 µg/mL TP (red box), IIRMIs, or a combination of 25 µg/mL TP+IIMIs for 24 hours. Supernatants were analyzed for the presence of cytokines by multiplex ELISA. Shown is the mean response (N=2). Shown here are the data generated using PBMC cultures of five representative donors. The data generated using PBMCs of additional five donors are presented in Figure 5. Normalized data used in Figures 5 and S11 are presented on Figure S12.

A. Flagellin

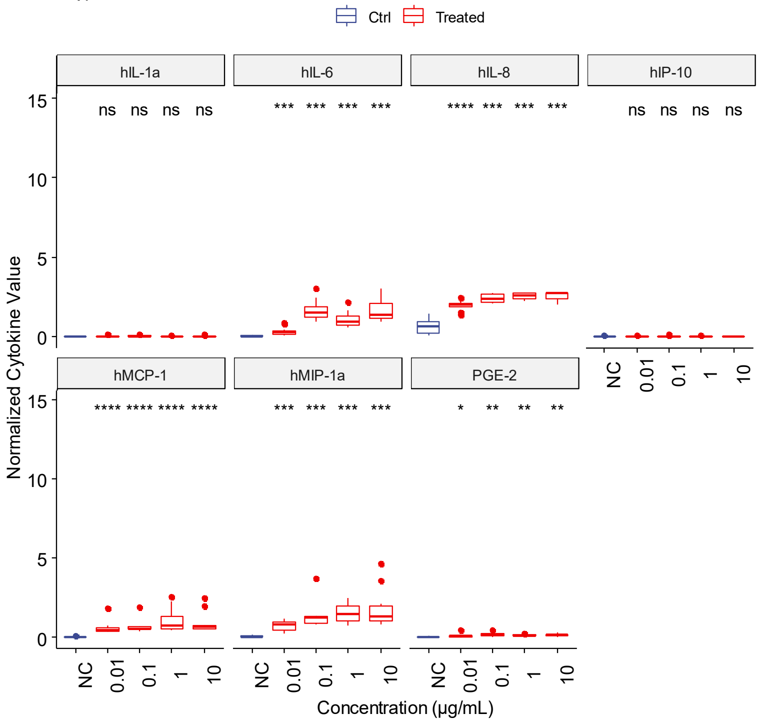

B. FSL-1

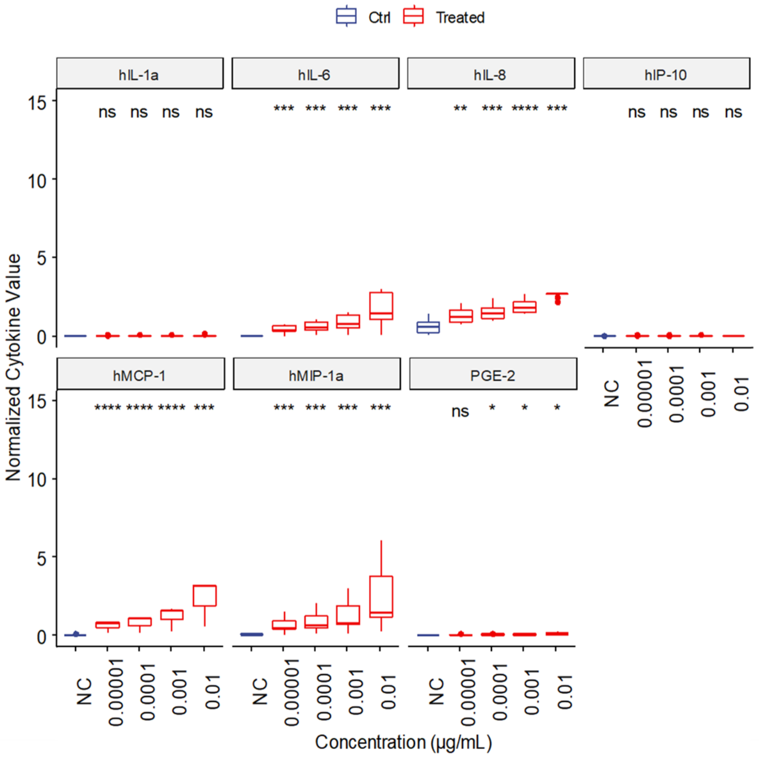

C. ODN2006

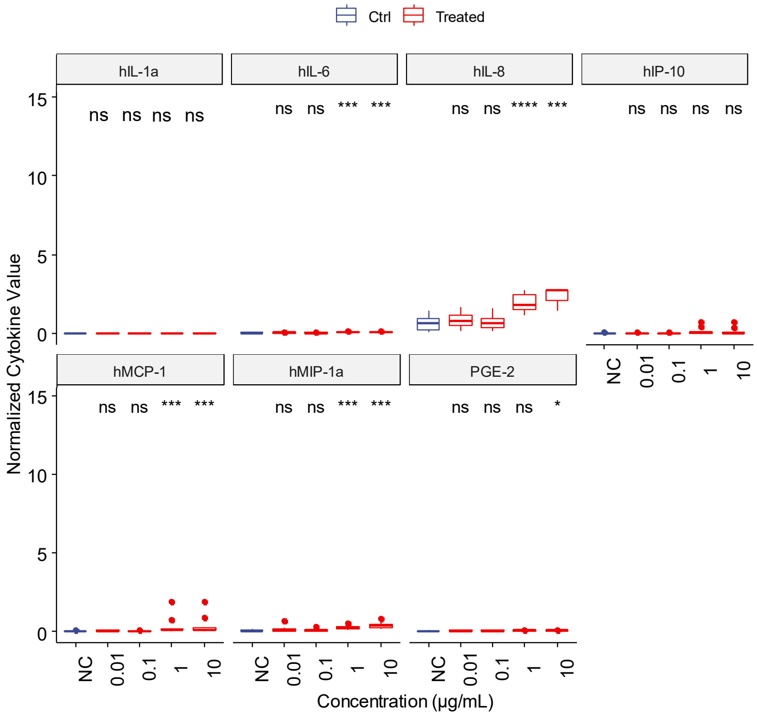

D. Poly(I:C) HMW

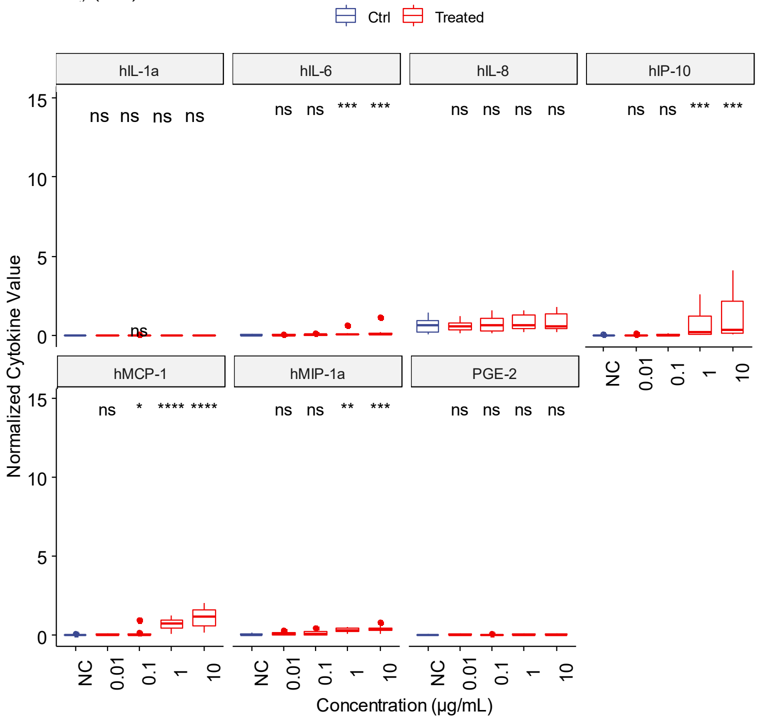

E. Poly(I:C) LMW

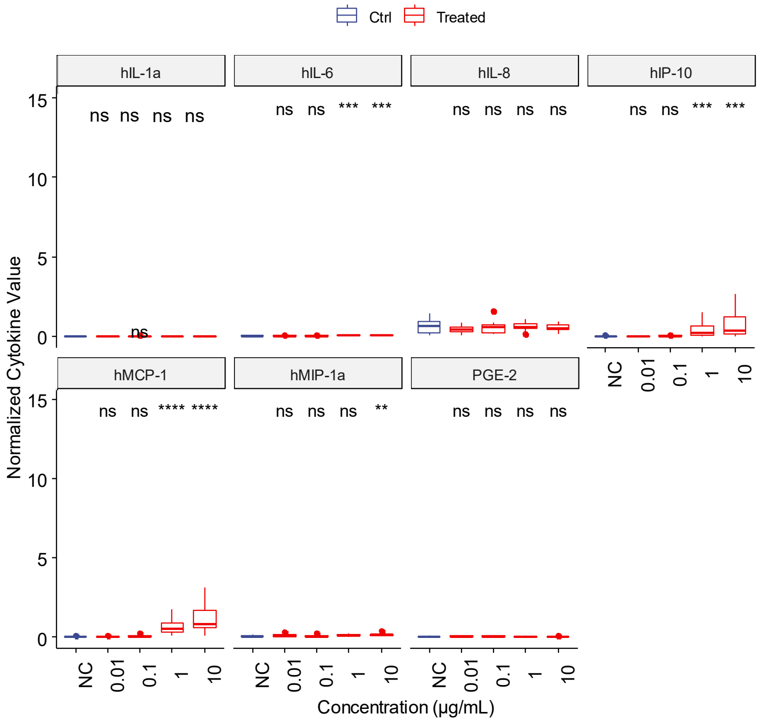

F. Zymosan

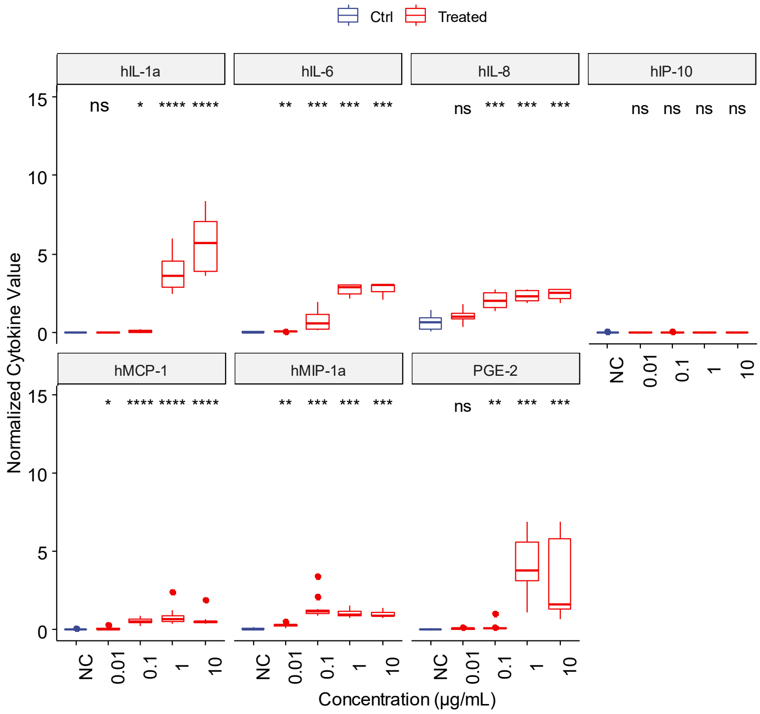

G. CLO75

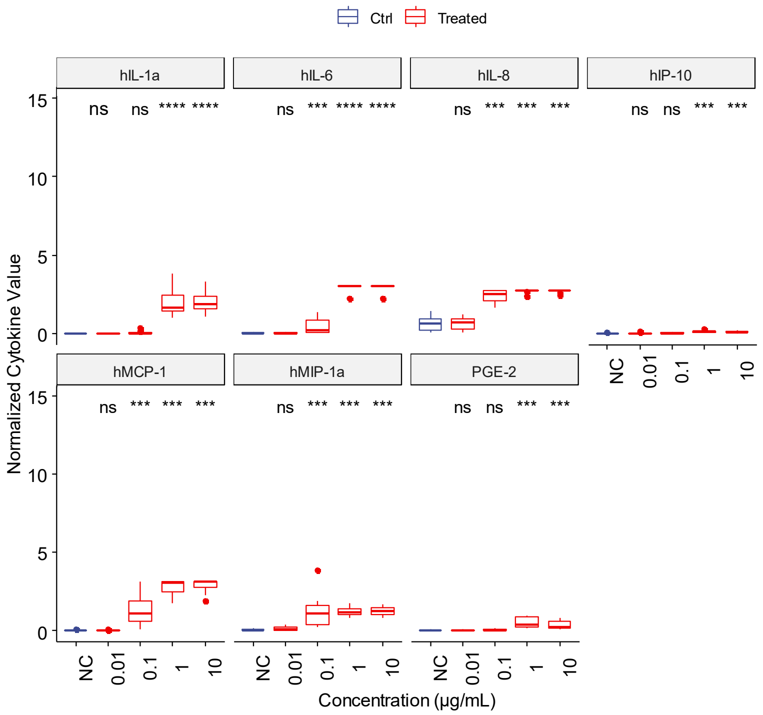
Ctrl
Treated
hIL-1a
hIL-6
hIL-8
hIP-10
hMCP-1
hMIP-1a
PGE-2
Normalized Cytokine Value
Concentration (μg/mL)
NC
0.01
0.1
1
10

H. MDP

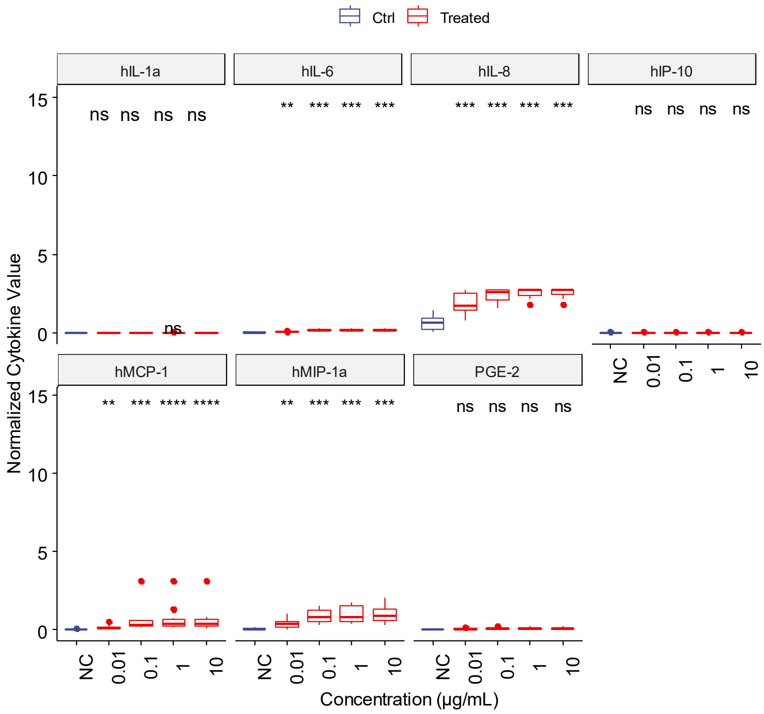
Ctrl
Treated
hIL-1a
hIL-6
hIL-8
hIP-10
hMCP-1
hMIP-1a
PGE-2
Normalized Cytokine Value
Concentration (μg/mL)
NC
0.01
0.1
1
10

I. ODN2216

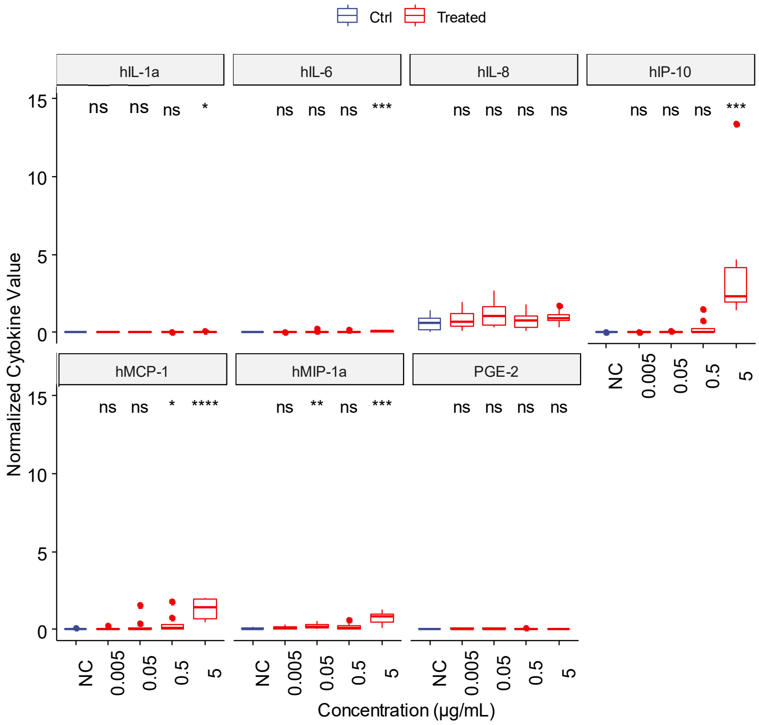

J. O111:B4 LPS

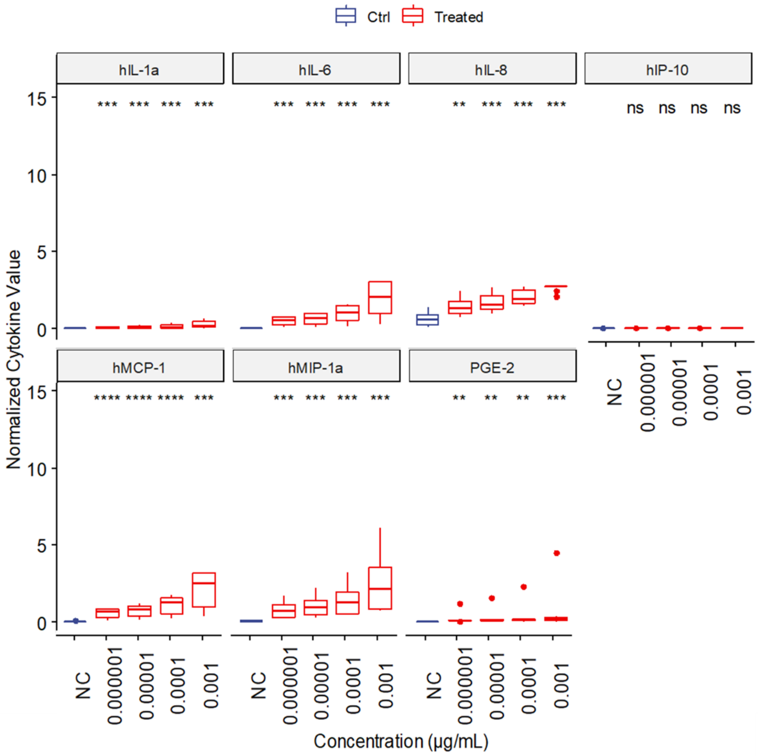

Figure S12. Normalized 7-plex Cytokine Response to Innate Immune Response Modulating Impurities. PBMCs from 10 healthy human donors were treated with IIRMIs alone for 24 hours. Supernatants were analyzed for the presence of cytokines by multiplex ELISA. Shown are the mean cytokine responses to individual IIRMI concentrations (red) of (A) flagellin, (B) FSL-1, (C) ODN2006, (D) poly(I:C) HMW, (E) poly(I:C) LMW, (F) zymosan, (G) CLO75, (H) MDP, (I) ODN2216, and (J) O111:B4 LPS as well as negative control (NC, blue). The data used to prepare this figure are shown on Figures 5 and S11. The data for which statistical significance was not observed are marked with ns. Statistical significance is shown with an asterisk as follows: *$p<0.05$; **$p<0.01$; ***$p<0.001$; and ****$p<0.0001$.

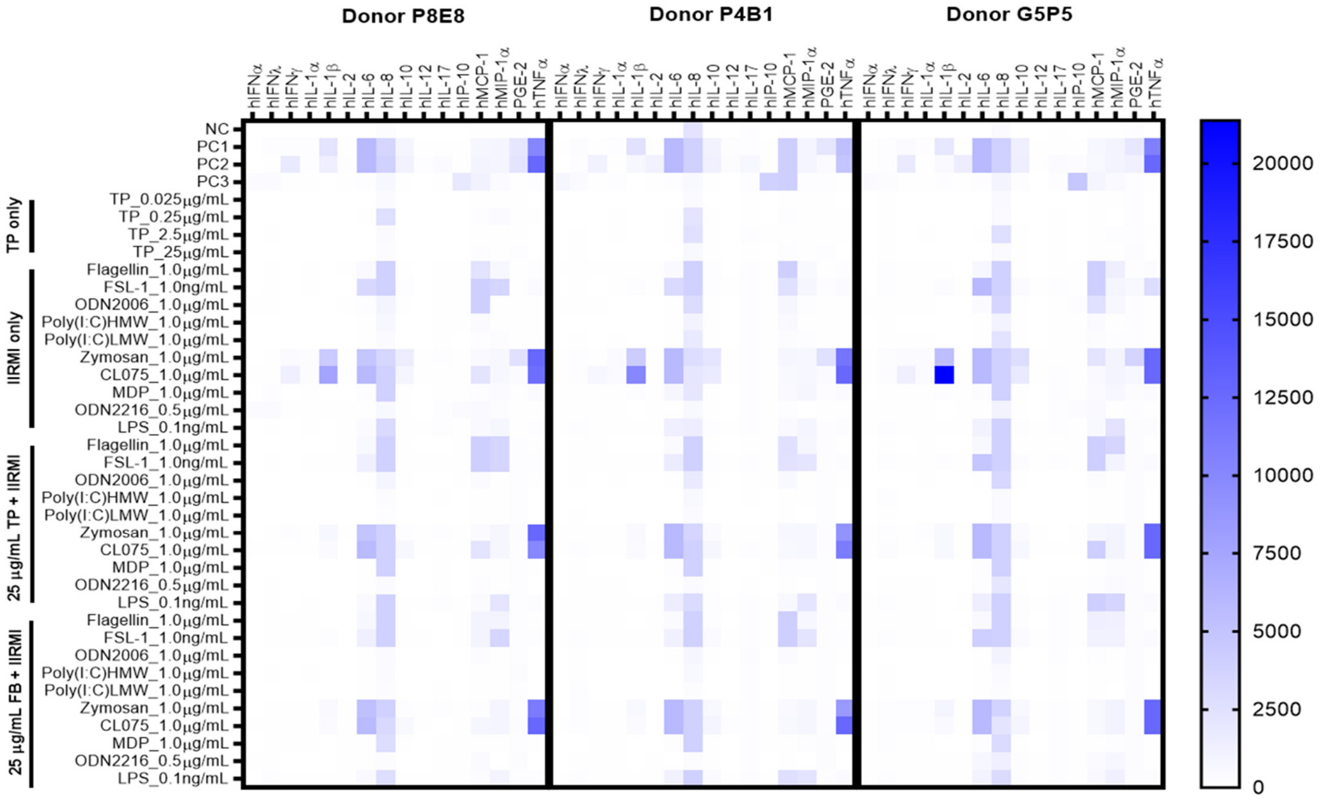

Figure S13. Formulation Buffer Affects Cytokines Induced by Innate Immune Response Modulating Impurities. To test the effects of TP and FB on IIRMI-induced cytokines, PBMCs collected from three healthy human donors, were treated with 0.025, 0.25, 2.5, and 25 µg/mL of TP alone, IIRMIs alone at the second highest concentration – see Table 5, or IIRMIs in combination with either 25 µg/mL TP or equivalent FB for 24 hours. Supernatants were analyzed for the presence of cytokines by multiplex ELISA. Shown is the mean response (N=2).

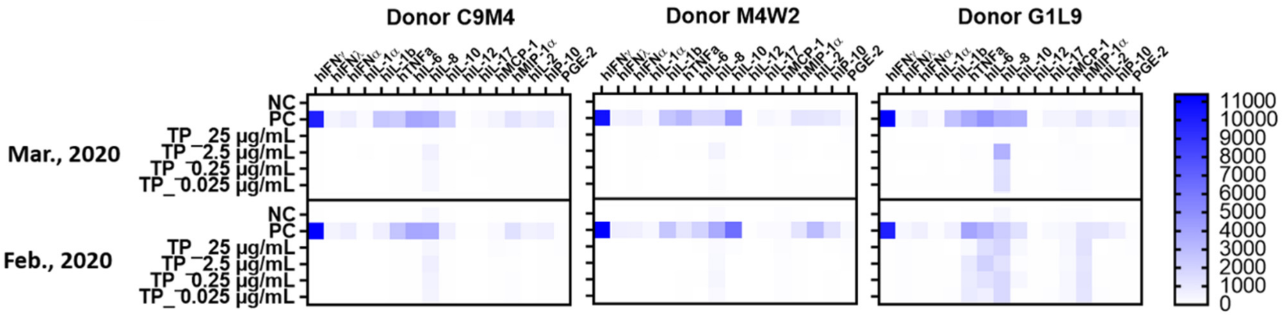

Figure S14. Reproducibility of Cytokine Response to Teriparatide in PBMC Cultures. PBMC from three healthy human donors were isolated in February 2020 (Feb) and again in March 2020 (Mar) for use in cytokine experiments. After each collection, the cells were treated with 0.025, 0.25, 2.5, and 25 µg/mL TP for 24 hours. Then supernatants were analyzed for the presence of cytokines by multiplex ELISA. Each data point represents a mean response (N=2).

A. 7-plex Cultured PBMCs

A. Flagellin

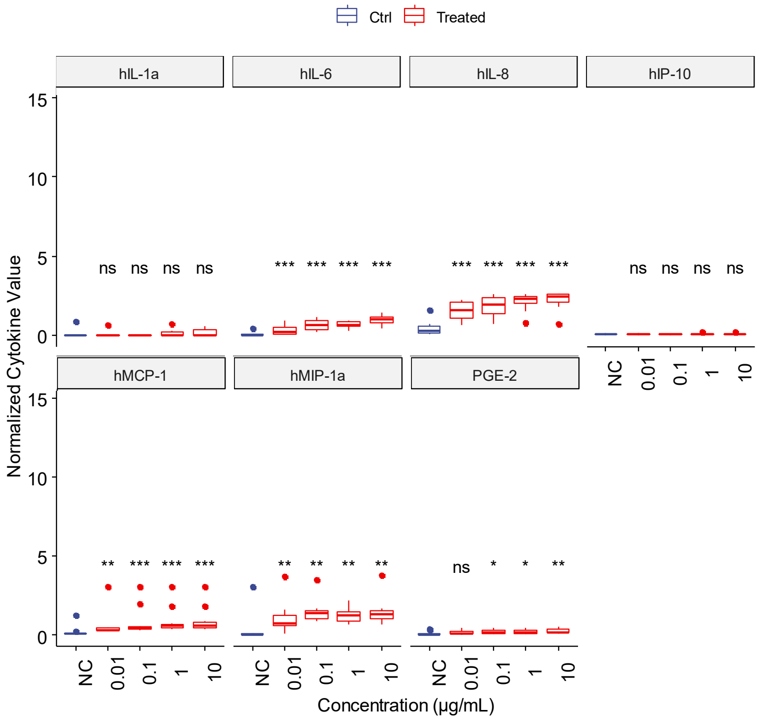

B. FSL-1

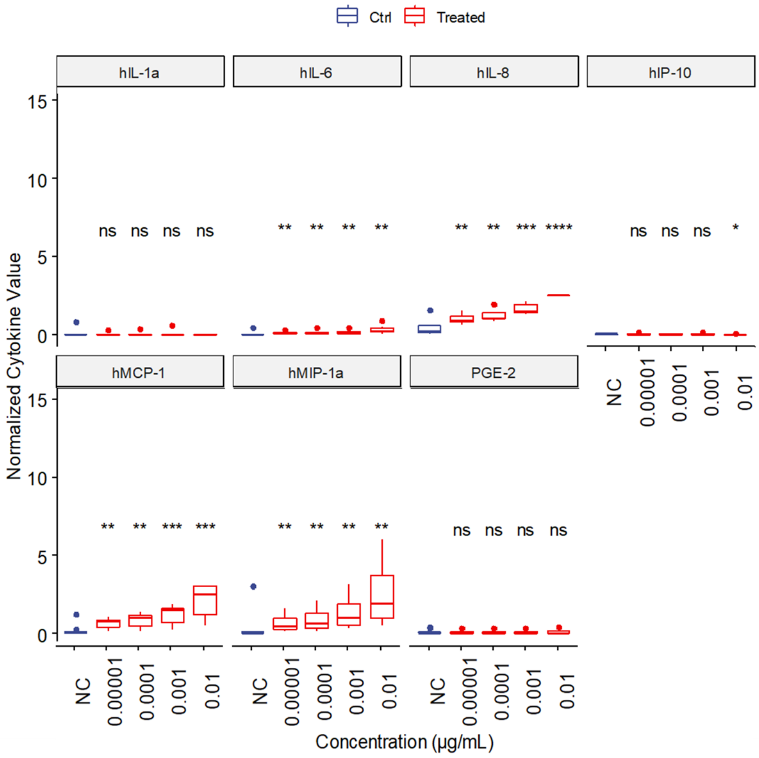

C. ODN2006

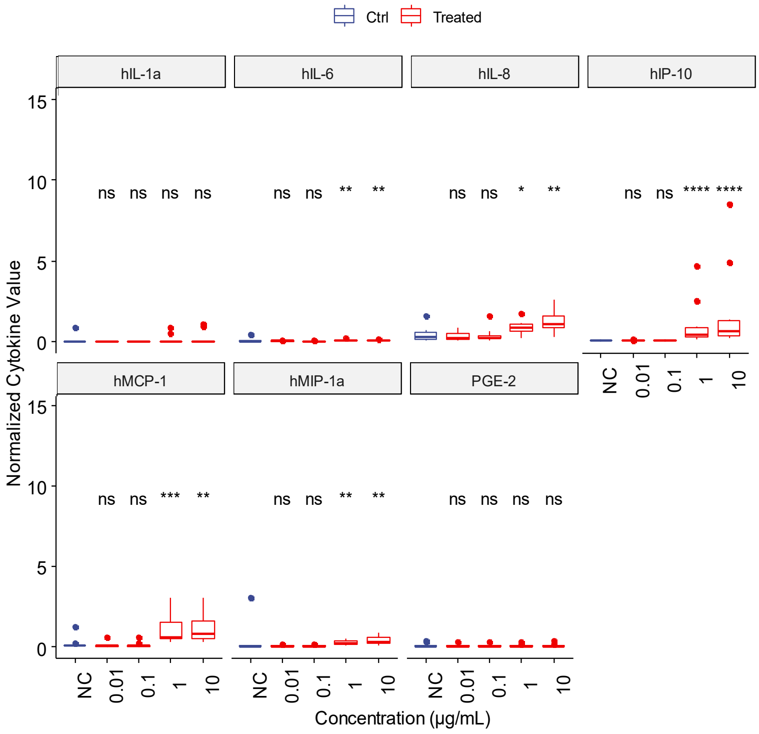

D. Poly(I:C) HMW

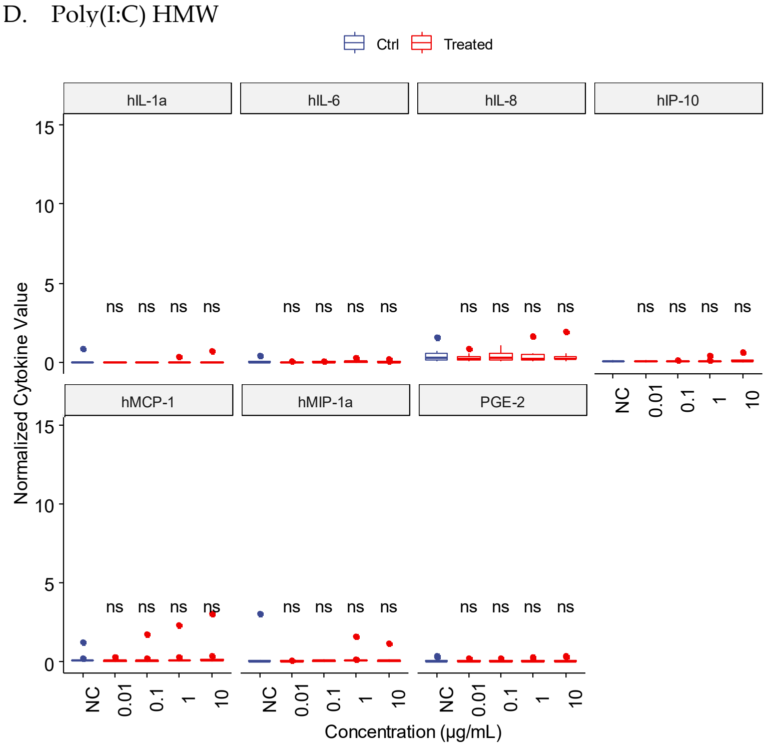

E. Poly(I:C) LMW

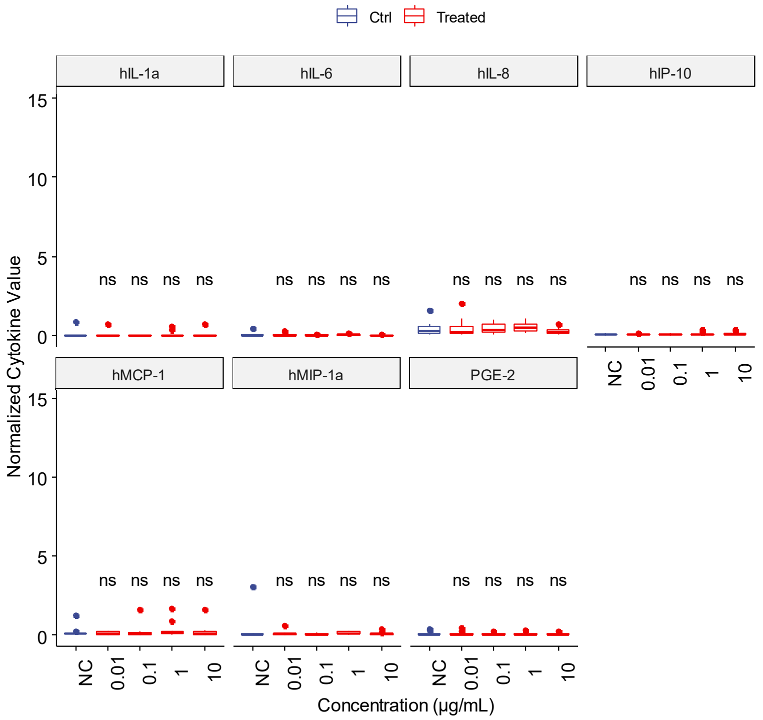

F. Zymosan

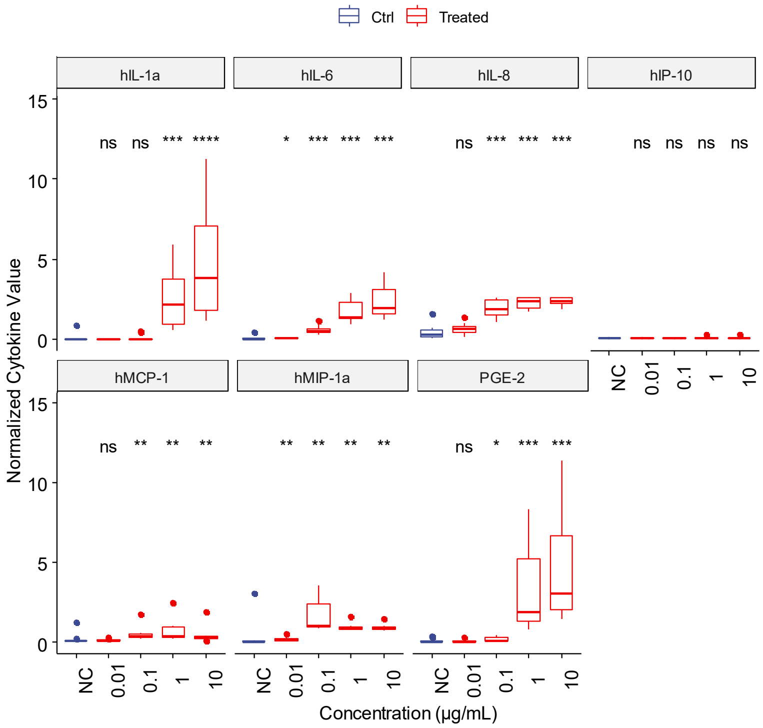

G. CLO75

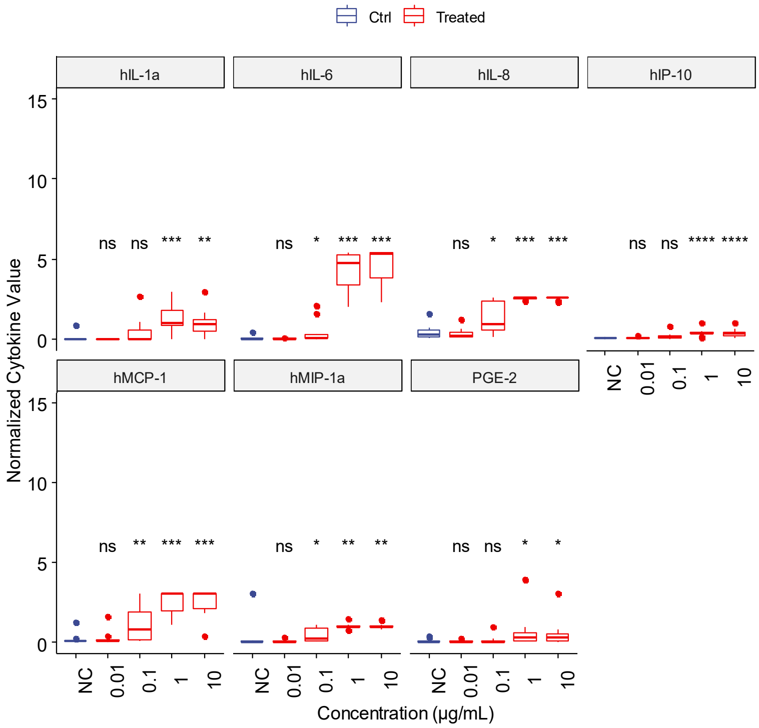

H. MDP

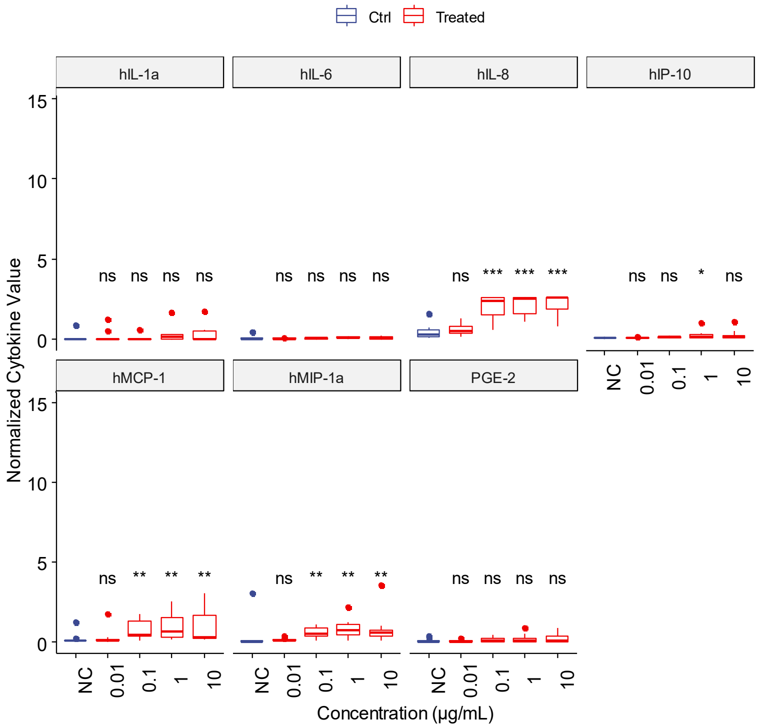

I. ODN2216

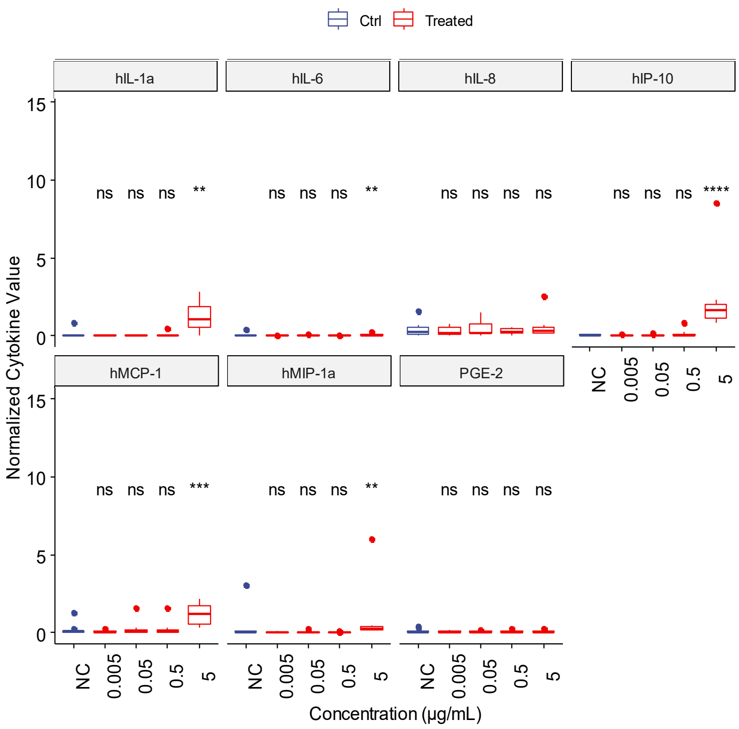

J. O111:B4 LPS

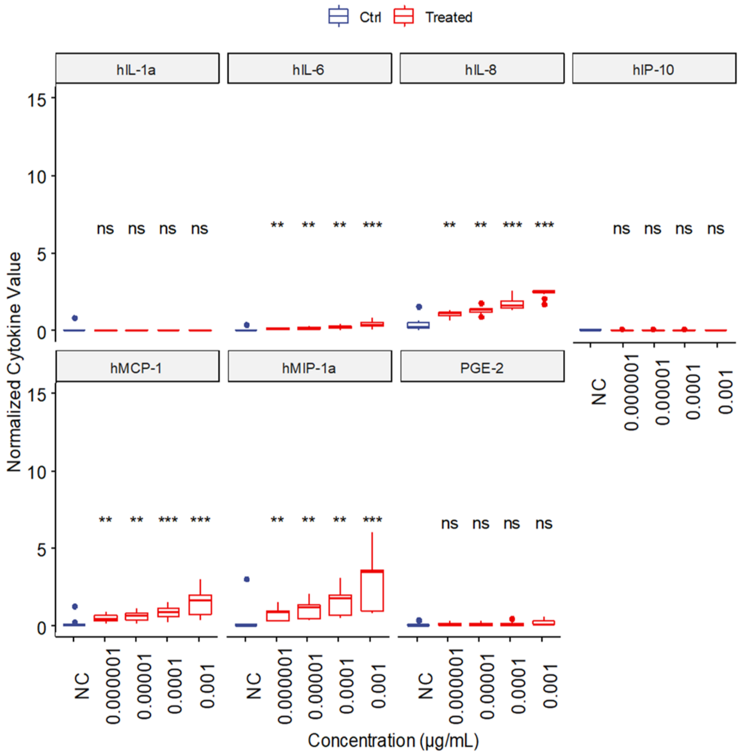

Figure S15A. Normalized Cytokine Responses to Innate Immune Response Modulating Impurities are Affected by PBMC and Blood Handling Conditions. PBMCs from 10 healthy human donors were exposed to various common laboratory handling conditions: A. cultured for 24 hours, B. cryopreserved, C. whole blood cultures, and D. isolated from blood stored for 24 hours or 48 hours (as compared to PBMCs isolated from fresh blood – see Figure 5) before being treated with IIRMIs alone for 24 hours. Supernatants were analyzed for the presence of cytokines by multiplex ELISA. Shown are the mean cytokine responses to individual IIRMI concentrations (red) of (A) flagellin, (B) FSL-1, (C) ODN2006, (D) poly(I:C) HMW, (E) poly(I:C) LMW, (F) zymosan, (G) CLO75, (H) MDP, (I) ODN2216, and (J) O111:B4 LPS as well as negative control (NC, blue). The data for which statistical significance was not observed are marked with ns. Statistical significance is shown with an asterisk as follows: *p<0.05; **p<0.01; ***p<0.001; and ****p<0.0001.

B. 7-plex Cryopreserved PBMCs

A. Flagellin

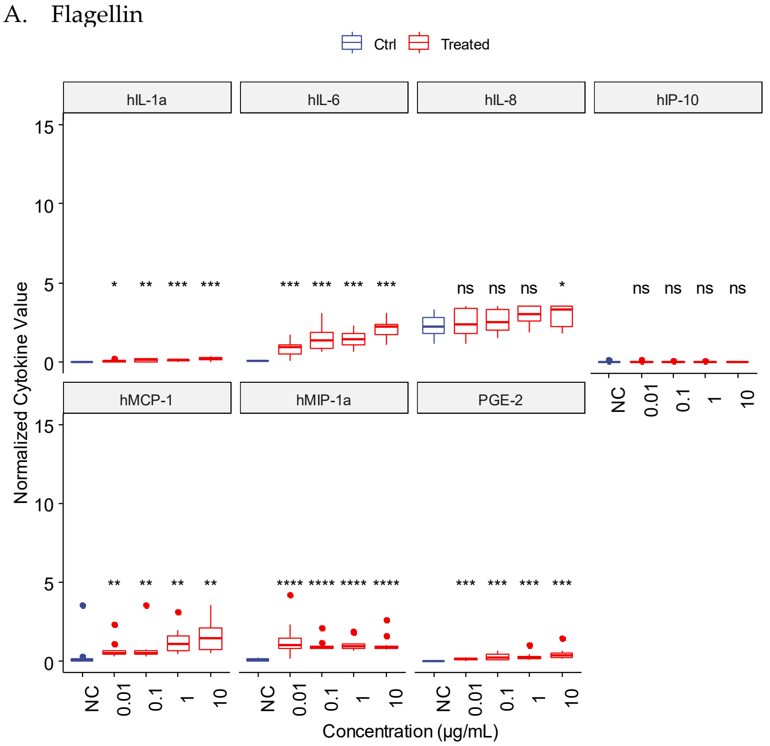

B. FSL-1

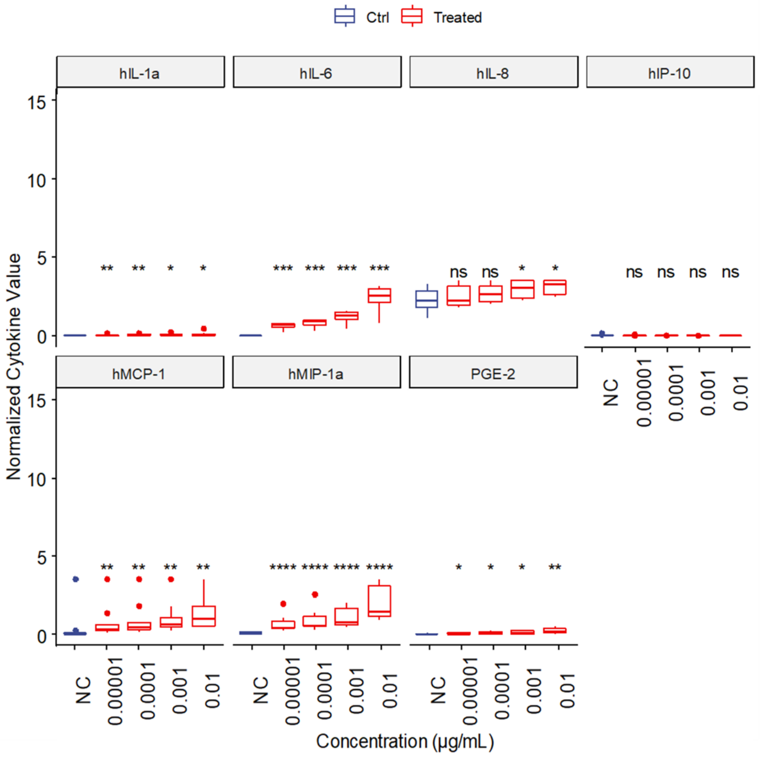

C. ODN2006

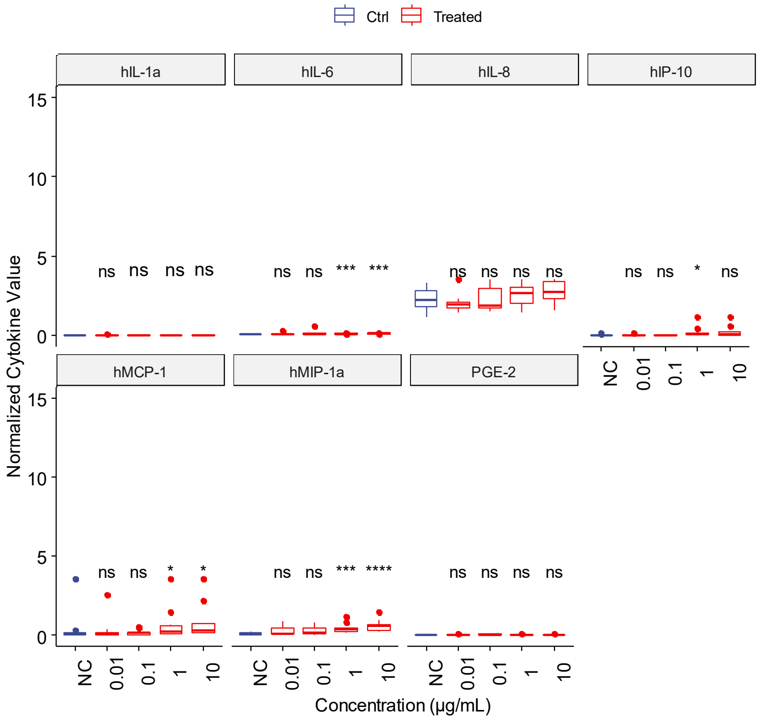

D. Poly(I:C) HMW

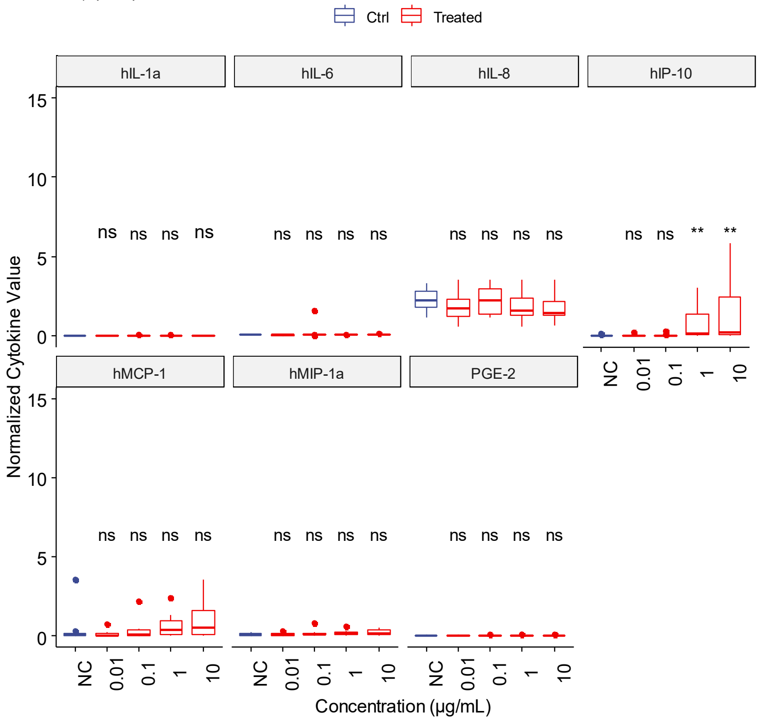

E. Poly(I:C) LMW

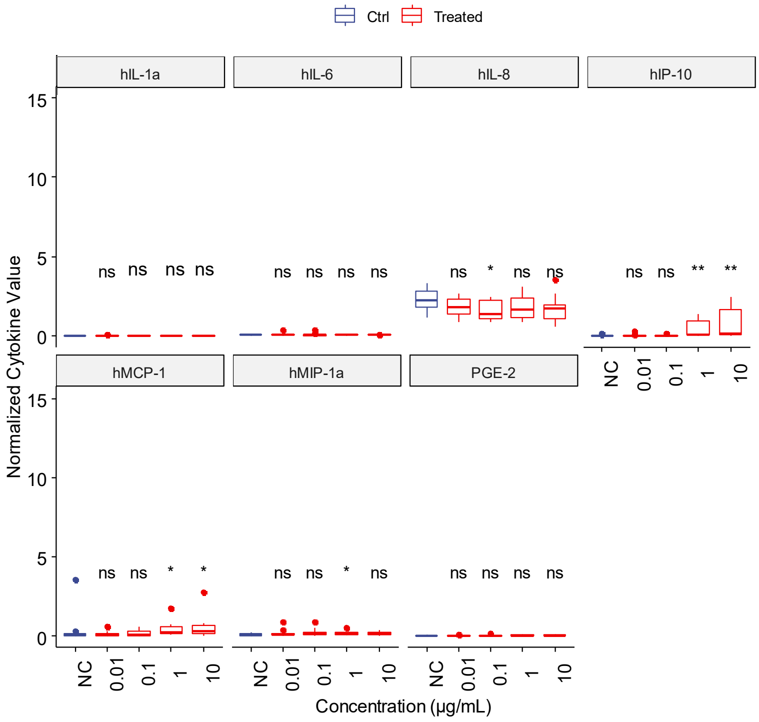

F. Zymosan

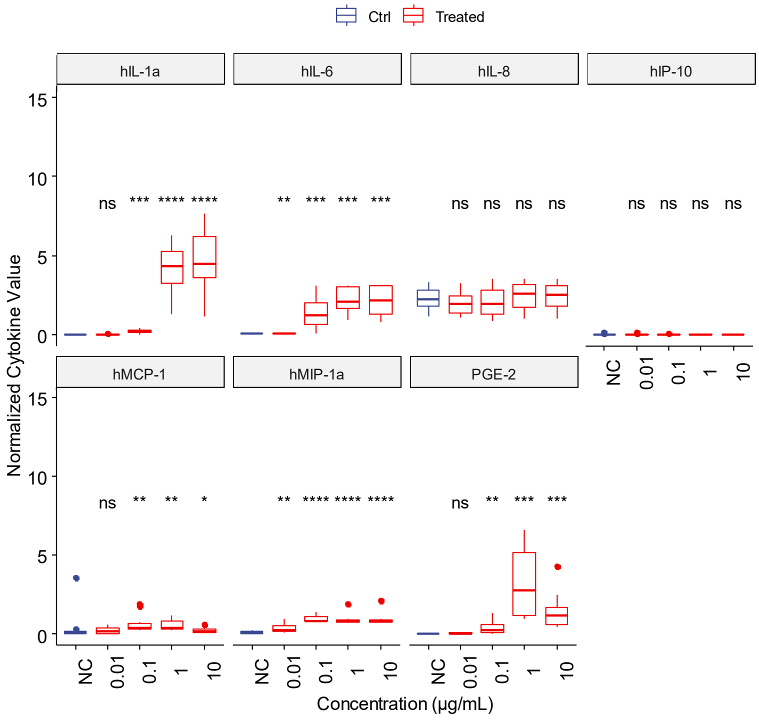

G. CLO75

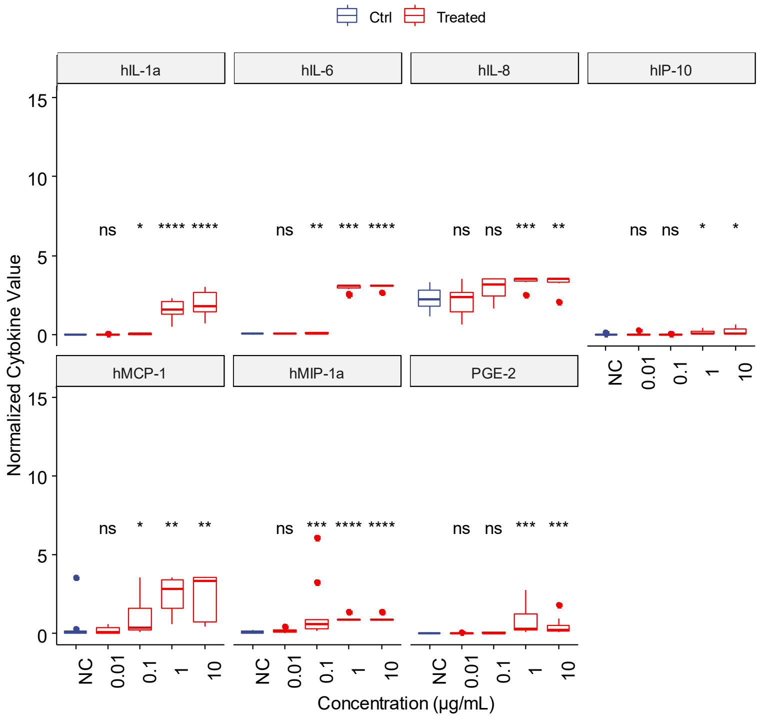

Ctrl
Treated
hIL-1a
hIL-6
hIL-8
hIP-10
hMCP-1
hMIP-1a
PGE-2
Normalized Cytokine Value
Concentration (µg/mL)
NC
0.01
0.1
1
10

H. MDP

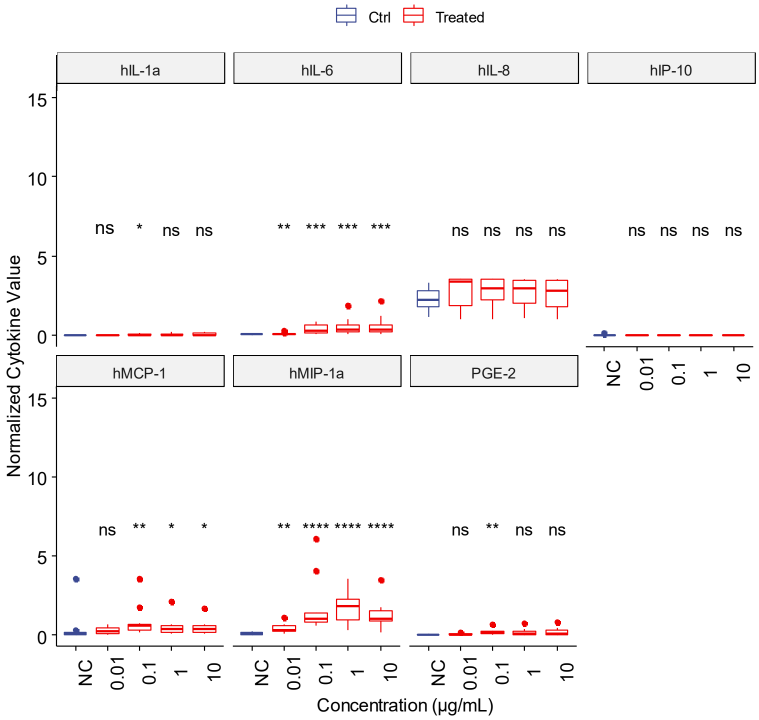

Ctrl
Treated
hIL-1a
hIL-6
hIL-8
hIP-10
hMCP-1
hMIP-1a
PGE-2
Normalized Cytokine Value
Concentration (µg/mL)
NC
0.01
0.1
1
10

I. ODN2216

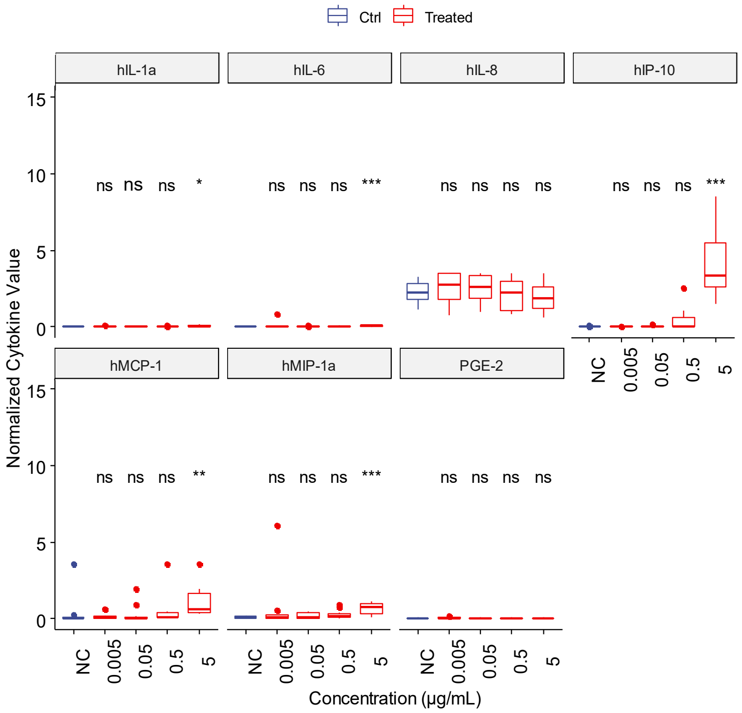

J. O111:B4 LPS

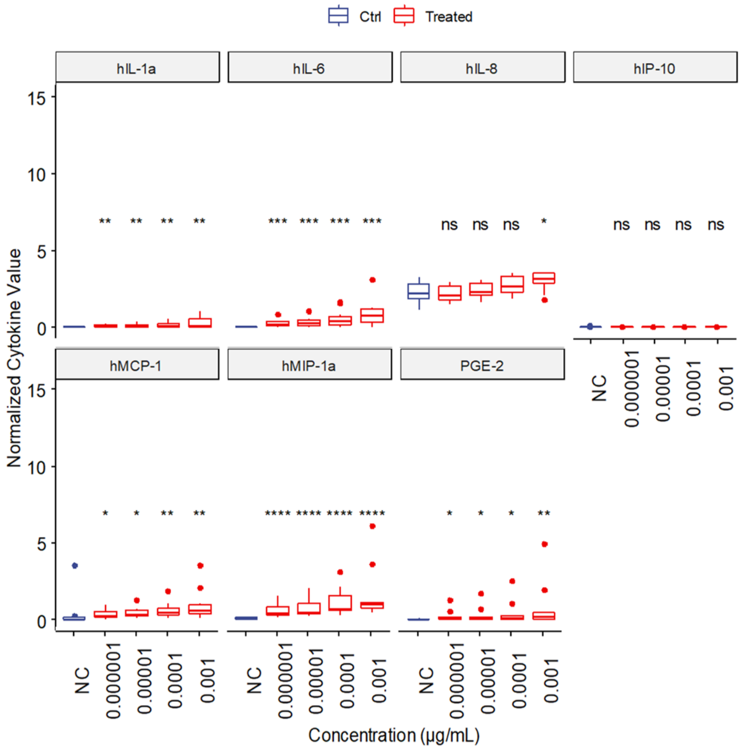

Figure S15B. Normalized Cytokine Responses to Innate Immune Response Modulating Impurities are Affected by PBMC and Blood Handling Conditions. PBMCs from 10 healthy human donors were exposed to various common laboratory handling conditions: A. cultured for 24 hours, B. cryopreserved, C. whole blood cultures, and D. isolated from blood stored for 24 hours or 48 hours (as compared to PBMCs isolated from fresh blood – see Figure 5) before being treated with IIRMIs alone for 24 hours. Supernatants were analyzed for the presence of cytokines by multiplex ELISA. Shown are the mean cytokine responses to individual IIRMI concentrations (red) of (A) flagellin, (B) FSL-1, (C) ODN2006, (D) poly(I:C) HMW, (E) poly(I:C) LMW, (F) zymosan, (G) CLO75, (H) MDP, (I) ODN2216, and (J) O111:B4 LPS as well as negative control (NC, blue). The data for which statistical significance was not observed are marked with ns. Statistical significance is shown with an asterisk as follows: *p<0.05; **p<0.01; ***p<0.001; and ****p<0.0001.

C. 7-plex Whole Blood Cultures

A. Flagellin

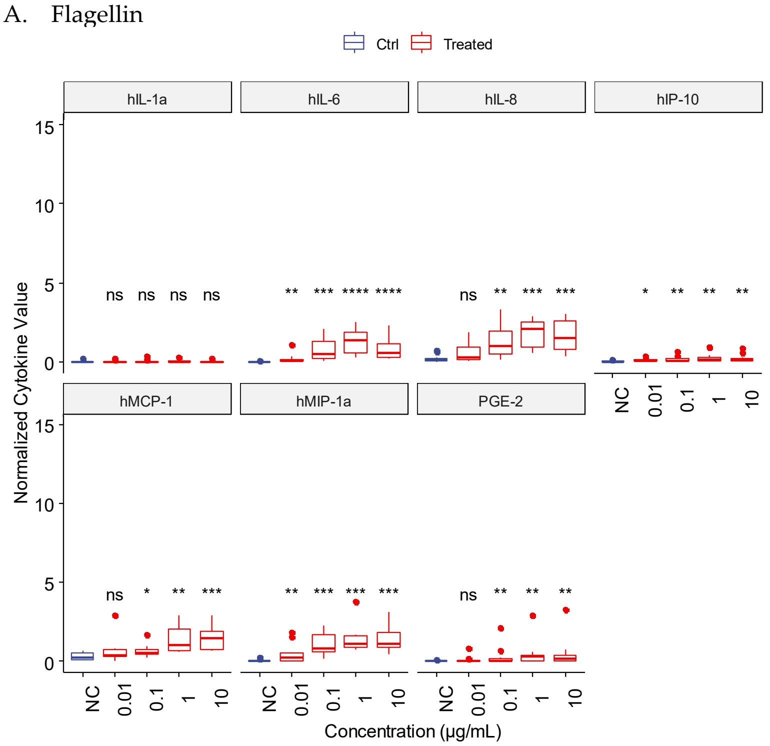

B. FSL-1

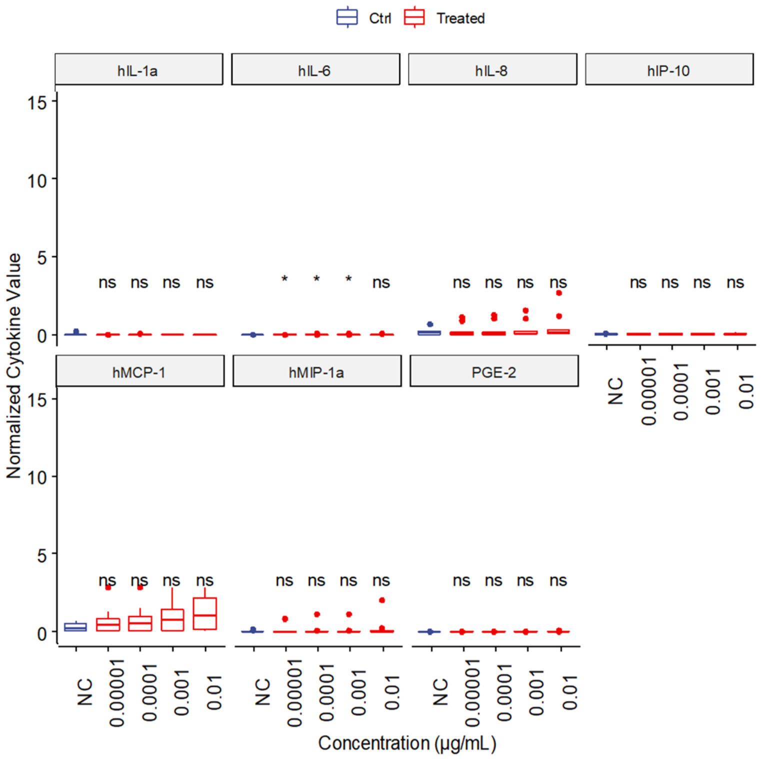

C. ODN2006

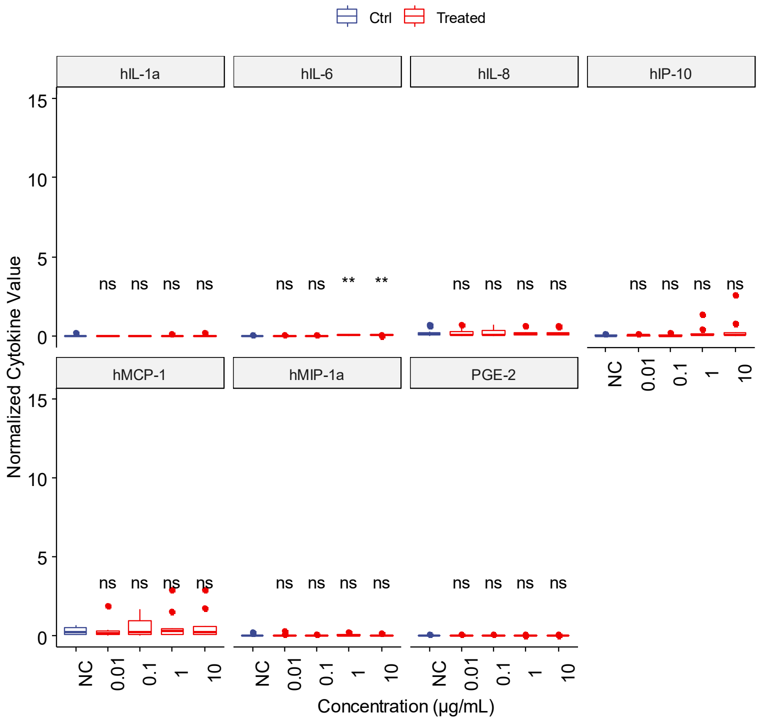

D. Poly(I:C) HMW

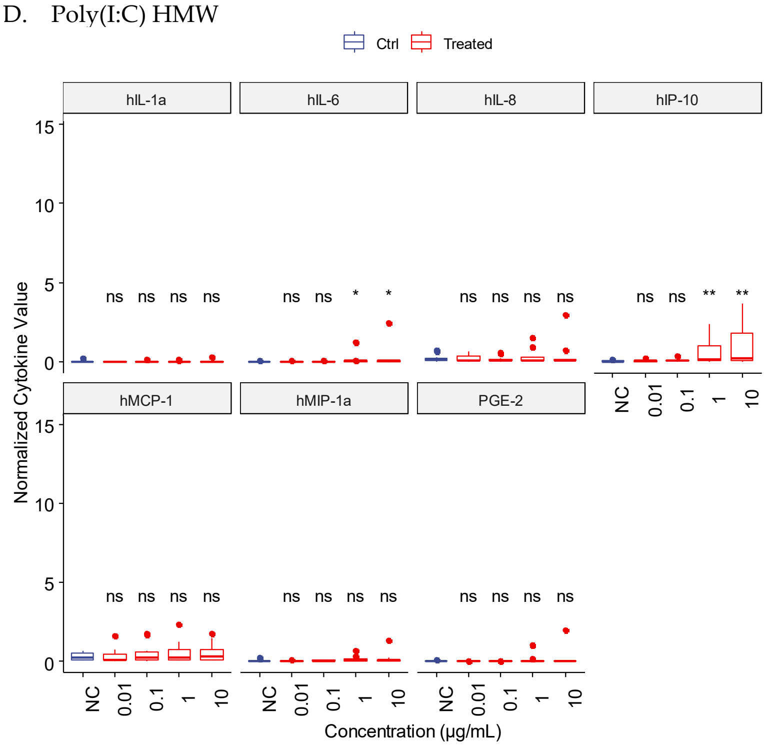

E. Poly(I:C) LMW

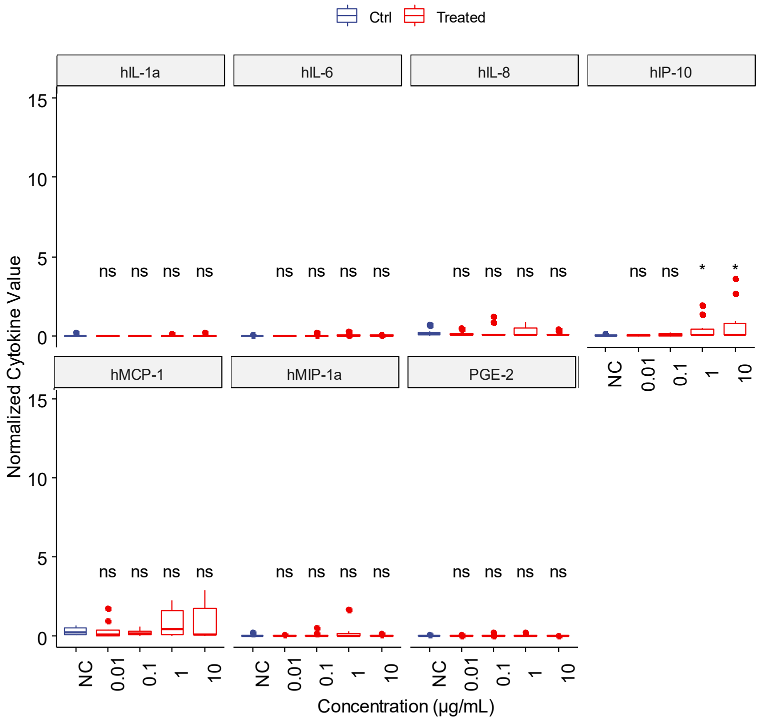

F. Zymosan

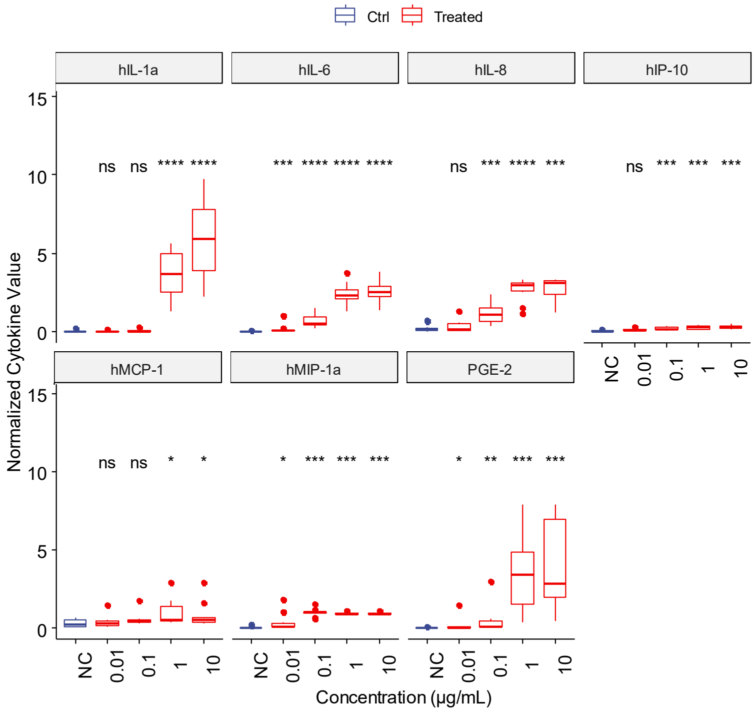

G. CLO75

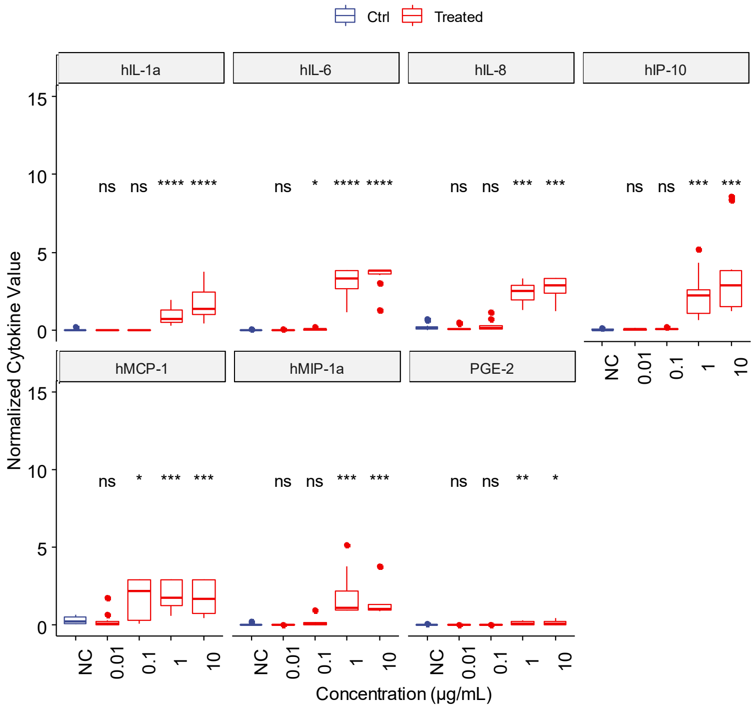
Ctrl
Treated
hIL-1a
hIL-6
hIL-8
hIP-10
hMCP-1
hMIP-1a
PGE-2
ns ns **** ****
ns * **** ****
ns ns *** ***
ns ns *** ***
ns * *** ***
ns ns *** ***
ns ns ** *
Normalized Cytokine Value
NC
0.01
0.1
1
10
Concentration (μg/mL)

H. MDP

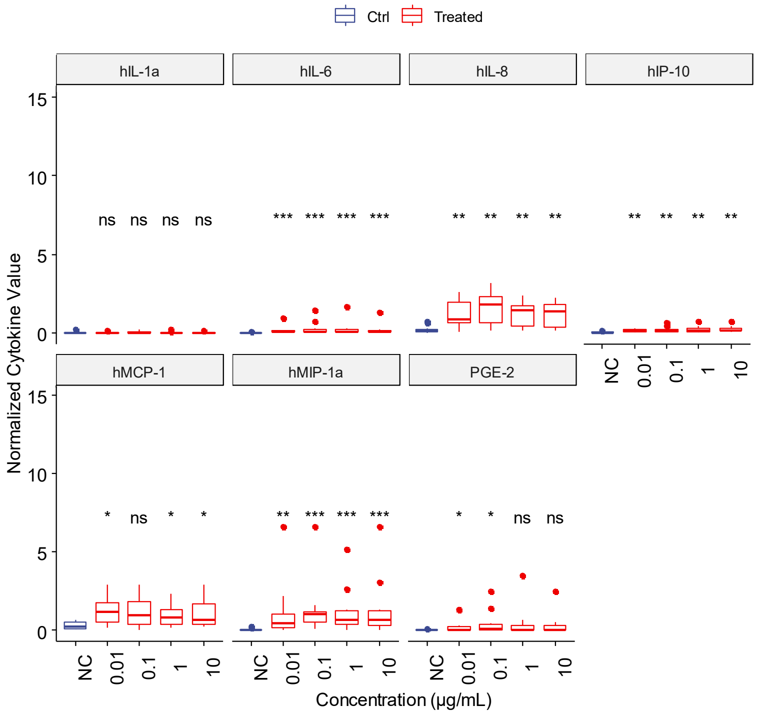
Ctrl
Treated
hIL-1a
hIL-6
hIL-8
hIP-10
hMCP-1
hMIP-1a
PGE-2
ns ns ns ns
*** *** *** ***
** ** ** **
** ** ** **
* ns * *
** *** *** ***
* * ns ns
Normalized Cytokine Value
NC
0.01
0.1
1
10
Concentration (μg/mL)

I. ODN2216

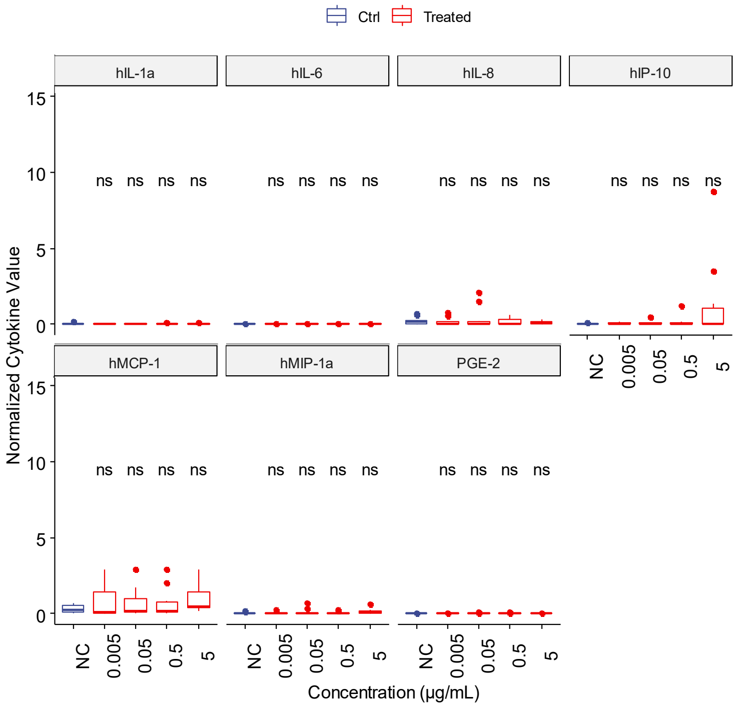

J. O111:B4 LPS

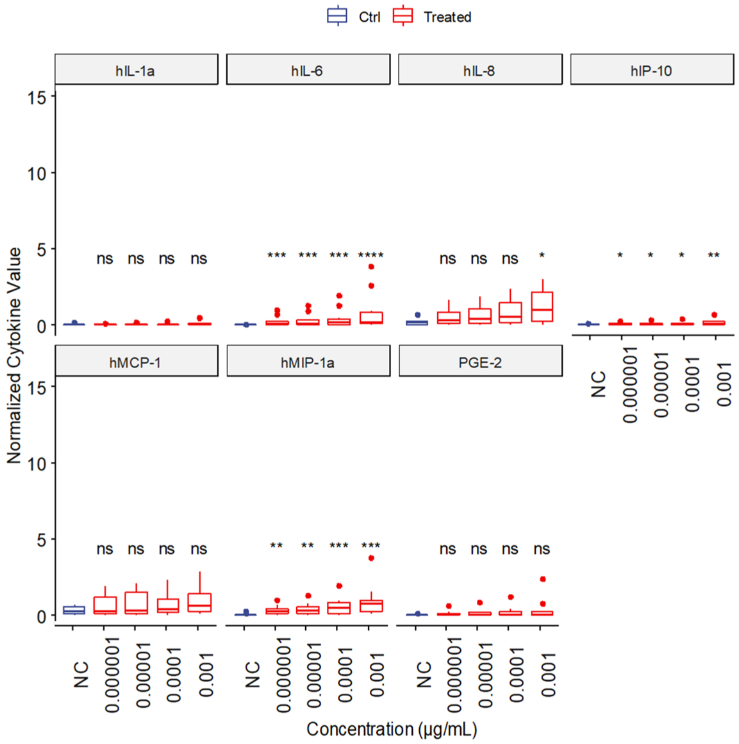

Figure S15C. Normalized Cytokine Responses to Innate Immune Response Modulating Impurities are Affected by PBMC and Blood Handling Conditions. PBMCs from 10 healthy human donors were exposed to various common laboratory handling conditions: A. cultured for 24 hours, B. cryopreserved, C. whole blood cultures, and D. isolated from blood stored for 24 hours or 48 hours (as compared to PBMCs isolated from fresh blood – see Figure 5) before being treated with IIRMIs alone for 24 hours. Supernatants were analyzed for the presence of cytokines by multiplex ELISA. Shown are the mean cytokine responses to individual IIRMI concentrations (red) of (A) flagellin, (B) FSL-1, (C) ODN2006, (D) poly(I:C) HMW, (E) poly(I:C) LMW, (F) zymosan, (G) CLO75, (H) MDP, (I) ODN2216, and (J) O111:B4 LPS as well as negative control (NC, blue). The data for which statistical significance was not observed are marked with ns. Statistical significance is shown with an asterisk as follows: *p<0.05; **p<0.01; ***p<0.001; and ****p<0.0001.

D. 7-plex PBMCs from Stored Blood

A. Flagellin – 24hr blood storage

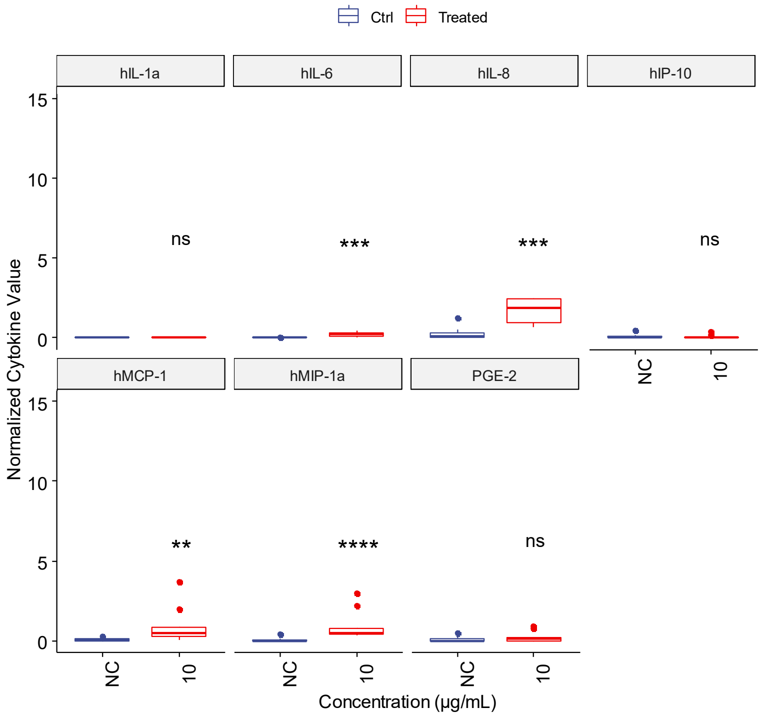

F. Zymosan – 24hr blood storage

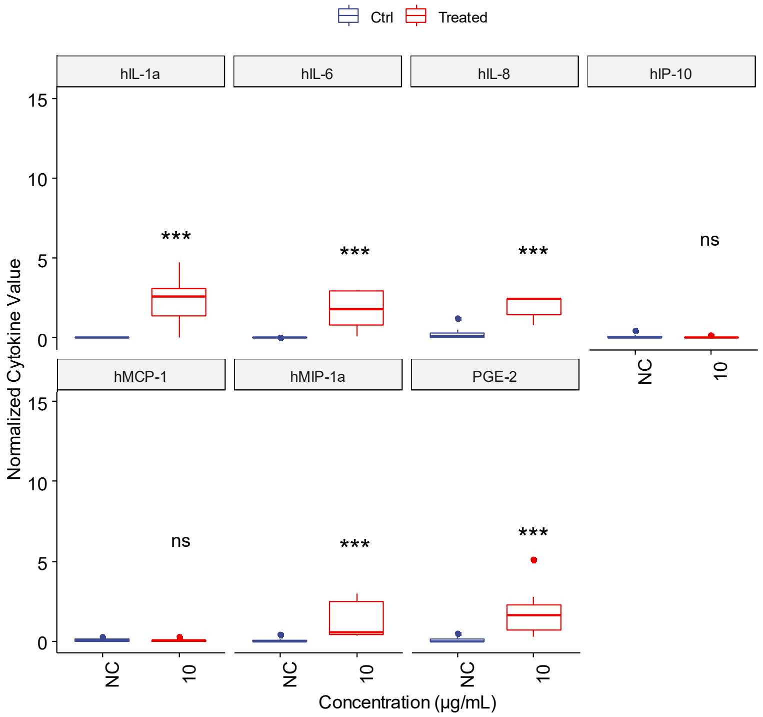

I. ODN2216 – 24hr blood storage

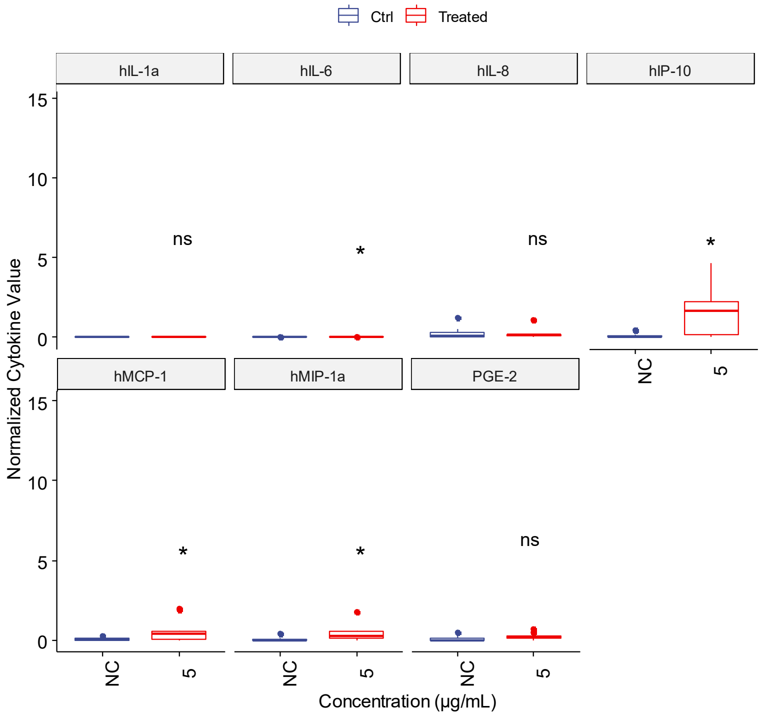

J. O111:B4 LPS – 24hr blood storage

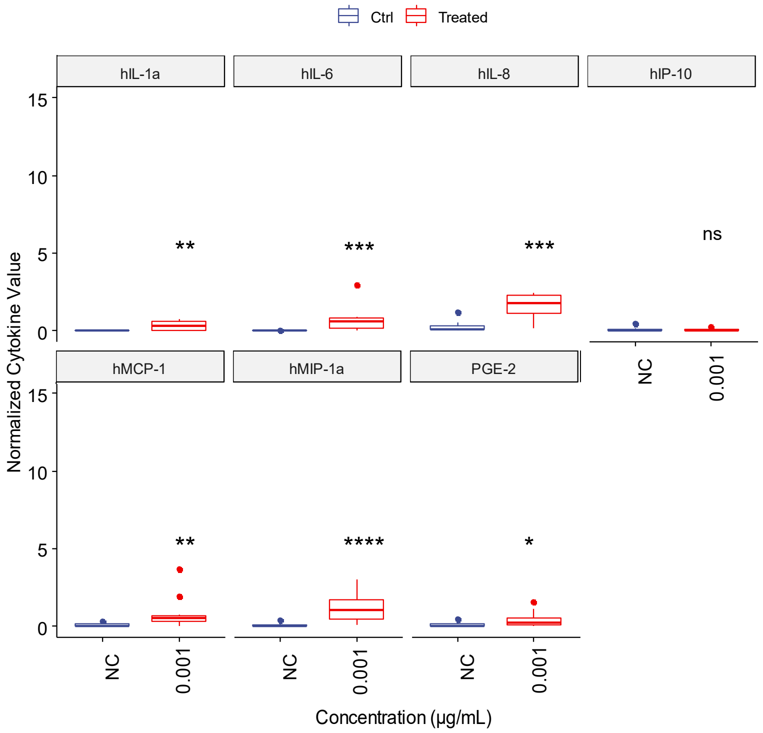

A. Flagellin – 48hr blood storage

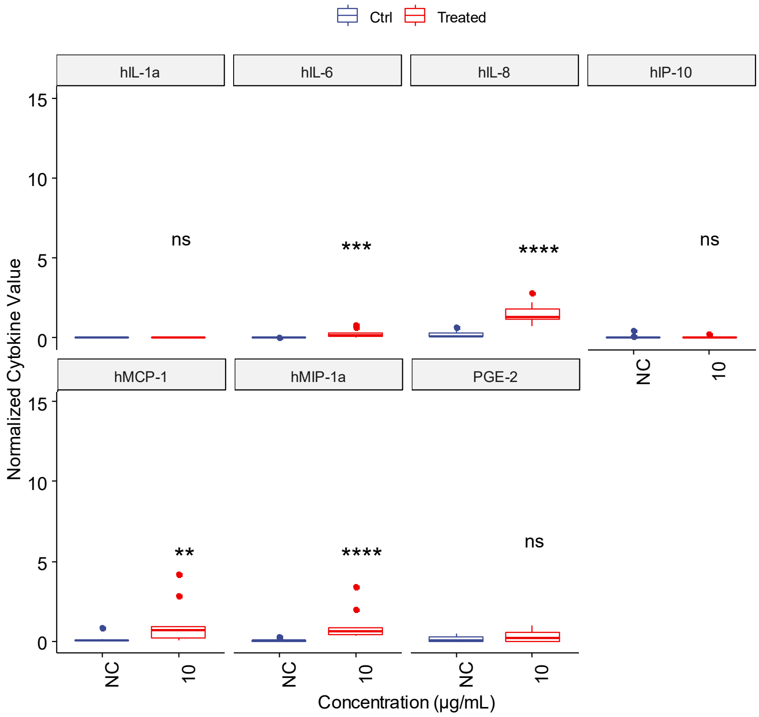

F. Zymosan – 48hr blood storage

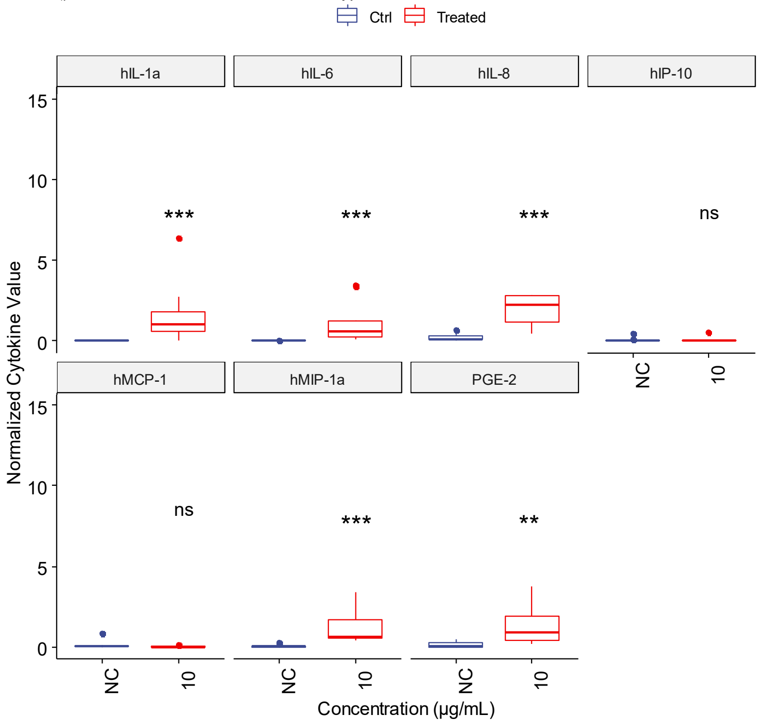

I. ODN2216 – 48hr blood storage

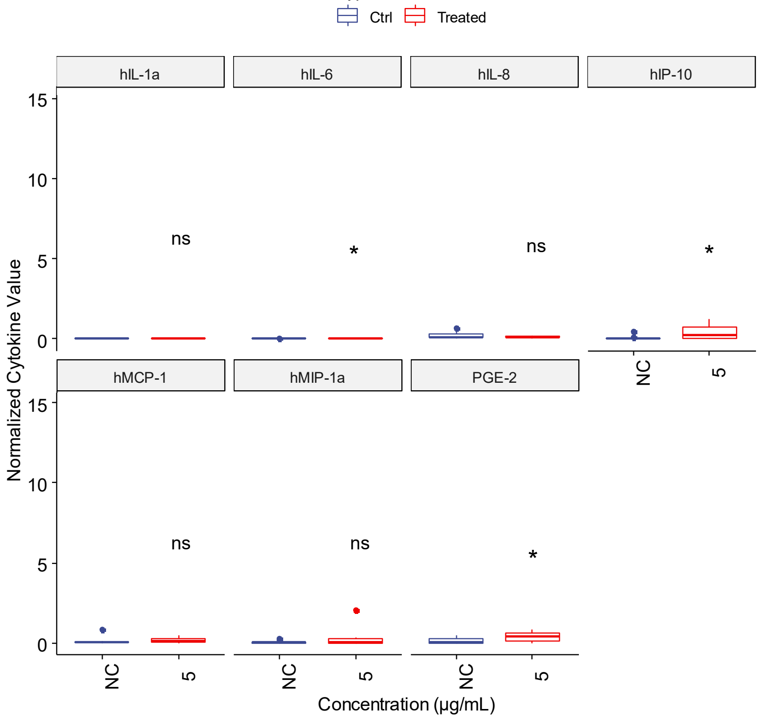

J. O111:B4 LPS – 48hr blood storage

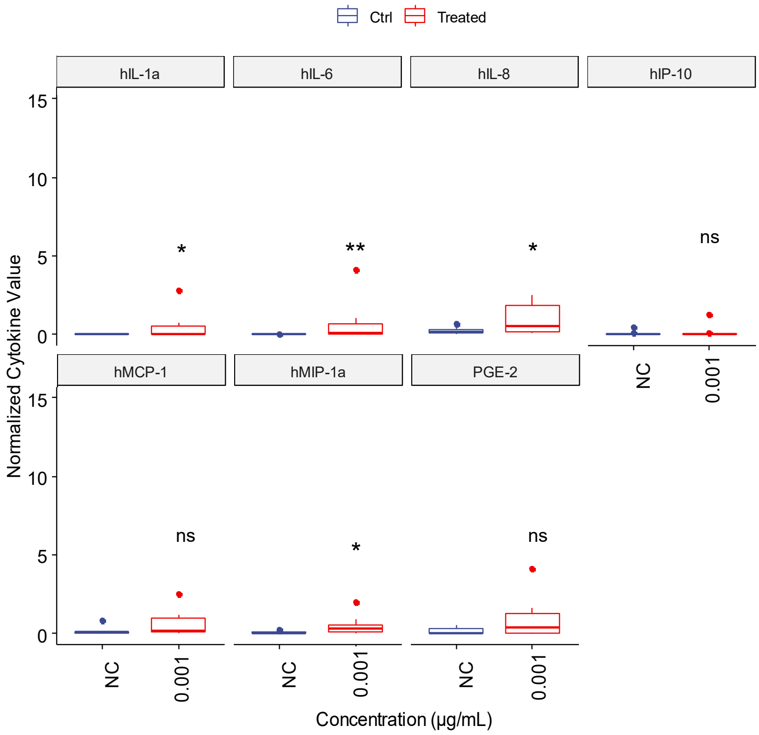

Figure S15D. Normalized Cytokine Responses to Innate Immune Response Modulating Impurities are Affected by PBMC and Blood Handling Conditions. PBMCs from 10 healthy human donors were exposed to various common laboratory handling conditions: A. cultured for 24 hours, B. cryopreserved, C. whole blood cultures, and D. isolated from blood stored for 24 hours or 48 hours (as compared to PBMCs isolated from fresh blood – see Figure 5) before being treated with IIRMIs alone for 24 hours. Supernatants were analyzed for the presence of cytokines by multiplex ELISA. Shown are the mean cytokine responses to individual IIRMI concentrations (red) of (A) flagellin, (B) FSL-1, (C) ODN2006, (D) poly(I:C) HMW, (E) poly(I:C) LMW, (F) zymosan, (G) CLO75, (H) MDP, (I) ODN2216, and (J) O111:B4 LPS as well as negative control (NC,

blue). The data for which statistical significance was not observed are marked with ns. Statistical significance is shown with an asterisk as follows: *p<0.05; **p<0.01; ***p<0.001; and ****p<0.0001.
